# Effect of *Bacillus velezensis* MT9 on Nile Tilapia (*Oreochromis Niloticus*) Intestinal Microbiota

**DOI:** 10.1007/s00248-025-02531-2

**Published:** 2025-05-01

**Authors:** Matteo Calcagnile, Elisa Quarta, Alessandro Sicuro, Laura Pecoraro, Roberta Schiavone, Salvatore Maurizio Tredici, Adelfia Talà, Angelo Corallo, Tiziano Verri, Loredana Stabili, Pietro Alifano

**Affiliations:** 1 Department of Experimental Medicine (DiMeS), University of Salento, Via Monteroni, 73100 Lecce, Italy; 2 Department of Biological and Environmental Sciences and Technologies (DiSTeBA), University of Salento, Via Monteroni, 73100 Lecce, Italy; 3 Institute of Water Research (IRSA), Istituto Talassografico “A. Cerruti”, National Research Council (CNR), Via Roma 3, 74123 Taranto, Italy; 4 National Biodiversity Future Center (NBFC), 90133 Palermo, Italy

**Keywords:** Nile tilapia, *B. velezensis* MT9, Intestinal microbiota, Probiotic, Aquaculture

## Abstract

In recent years, there has been a growing interest in the use of probiotics in aquaculture, due to their effectiveness on production, safety, and environmental friendliness. Probiotics, used as feed additives and as an alternative to antibiotics for disease prevention, have been shown to be active as growth promoters, improving survival and health of farmed fish. In this study, we have investigated the ability of the strain *Bacillus velezensis* MT9, as potential probiotic, to modulate the intestinal microbiota of the Nile tilapia (*Oreochromis niloticus*) fed with the *Bacillus velezensis*-supplemented feed in an experimental aquaculture plant. The analysis of the microbial community of the Nile tilapia by culture-based and 16S rRNA gene metabarcoding approaches demonstrated that *B. velezensis* MT9 reshapes the fish intestinal microbiota by reducing the amounts of opportunistic Gram-negative bacterial pathogens belonging to the phylum of Proteobacterium (Pseudomonadota) and increasing the amounts of beneficial bacteria belonging to the phyla Firmicutes (Bacillota) and Actinobacteria (Actinomycetota). Specifically, dietary supplementation of Nile tilapia with *B. velezensis* MT9 resulted in an increase in the relative abundance of bacteria of the genus *Romboutsia*, which has a well-documented probiotic activity, and a decrease in the relative abundance of Gammaproteobacteria of the genera *Aeromonas* and *Vibrio*, which include opportunistic pathogens for fish, and *Escherichia/Shigella*, which may pose a risk to consumers. The whole genome sequence of *B. velezensis* MT9 was then determined. Genome analysis revealed several peculiarities of *B. velezensis* MT9 compared to other *B. velezensis* reference strains including specific metabolic traits, differences in two-component and quorum sensing systems as well as the potential ability to produce a distinct array of secondary metabolites, which could explain the strong ability of this strain to modulate the intestinal microbiota of the Nile tilapia.

**Supplementary Information:**

The online version contains supplementary material available at 10.1007/s00248-025-02531-2.

## Introduction

The world’s population is expected to reach 9.7 billion by 2050 [1, 2], and global demand for animal proteins may increase by as much as 88% [3, 4]. Thus, one of the greatest challenges facing humanity today is how to feed a growing population a healthy (nutritious) and sustainable diet [5]. To meet this food demand, aquaculture is growing worldwide; it is expected to double by 2050 [6], and it currently represents one of the fastest growing industries globally [7]. An aquaculture system that, in the long term, improves environmental quality, reduces the impacts of overfishing on natural sources, and provides for human food needs, is economically sustainable and improves the quality of life of farmers and society as a whole [8–11]. Recent studies, with the advancement in fish farming technologies, have highlighted the need to derive more proteins from aquatic sources [5, 12, 13]. In recent years, global per capita seafood consumption has increased from 9.0 kg (live weight equivalent) in 1961 to 20.2 kg in 2020 [14], providing about 17% of the animal proteins consumed [14]. In this context, it is noteworthy that the Nile tilapia (*Oreochromis niloticus*) farming is becoming increasingly notable, moving towards replacing one or more carp species as the flagship farmed white-fleshed fish. Tilapia is a popular freshwater fish, prolific, easy to reproduce, and with high economic value. Furthermore, tilapia can live in both fresh and brackish water, can be reared at relatively low densities in fertilized ponds and in the effluents of fishponds or industrial sources. It can be raised in floating cages, earthen, and concrete ponds and is a palatable herbivorous fish [15]. The rapid growth of tilapia to a leading position worldwide is partly due to the large-scale availability of feed inputs in major producing countries. Feed is the most expensive component in the intensive aquaculture industry, where it accounts for more than 50% of operating costs. Producing sustainable feeds that support fish welfare and maximize growth potential while remaining cost-effective is a major challenge for the aquaculture industry [16]. Therefore, the task that tilapia farmers and fish nutritionists face together is to develop commercial and cost-effective tilapia feeds, while ensuring optimal fish growth and health, and also meeting market demands in terms of sustainability and wholesomeness of the product.

This study aimed to apply a sustainable technology/approach for the production of a live microorganism-enriched feed that delivers active ingredients beneficial to the health of the farmed species and consumers [4]. In recent years, probiotics have been widely utilized in aquaculture due to their effectiveness on production, safety, and environmental friendliness [17, 18]. These probiotics, employed as feed additives and an alternative to antibiotics for disease prevention, have been found to be active as growth promoters for the individual growth, survival, and health of farmed species, facilitating digestion, improving antioxidant capacity, regulating immunity, and suppressing the pathogens infection [17, 19–22]. However, probiotics also confer several benefits by modulating the gut microbiome and playing an important role in reducing the toxic effects of some pollutants in aquaculture [23, 24]. For example, recent research has proven that probiotic supplementation in zebrafish alleviated intestinal microbial dysbiosis, lipid metabolism disorders, developmental toxicity, and growth retardation due to perfluoro butane sulfonate (PFBS) [25–27]. Furthermore, it is well known that gut microbial-derived metabolites are associated with host health and disease, and probiotics, by virtue of their biochemical activities, could optimize the metabolic profiles of gut microbiota, thereby modulating intestinal metabolism [28–30]. For instance, *Bacillus cereus* added to the diet, promoted the growth, increased the immunity and antioxidant capacity of Pengze crucian carp (*Carassius auratus* var. Pengze) [31] as well as it improved the growth performance of rohu (*Labeo rohita*) through upgrading hematological parameters and the intestinal microbiota [29]. The literature also shows the effective use of bacterial strains of the genus *Bacillus*, including those isolated from soil, as probiotics in aquaculture [32, 33]. The ability to produce secondary metabolites, including several antibacterial compounds, is a typical property of many *Bacillus* spp. and is considered an advantageous feature for probiotics [34, 35].

Taking all above into consideration, the present study investigated the potential role of the new selected strain *Bacillus velezensis* MT9, as potential probiotic, in modifying the intestinal microbiota of Nile tilapia fed with this live microorganism-supplemented feed. This bacterium was previously isolated from soil samples and showed both bactericidal activity in vitro and the ability to enhance plant health in vivo [36]. This species was selected because it has been widely used as an experimental probiotic in aquaculture without posing safety concerns [32, 34, 37–43]. Furthermore, the European Food Safety Authority (EFSA) has included *B. velezensis* in its list of substances subject to Qualified Presumption of Safety (QPS) [44].

In the present study, we aimed at characterizing *B. velezensis* MT9 at the genomic level in order to identify any favorable characteristics, also compared to other strains of the same species, and to analyze its ability to positively modulate the microbiota of Nile tilapia, antagonizing groups of microorganisms of significant pathogenic interest in aquaculture. This information will be valuable in determining the suitability of *B. velezensis* MT9 as a novel probiotic for Nile tilapia. In particular, we provided a microbial inventory based on conventional cultural methods, integrated with the 16S rRNA gene metabarcoding approach for bacterial identification and diversity analyses of tilapia intestinal microbiota. We focused on the intestinal microbial community because, in addition to the digestion function, the intestine is a typical site for microbial colonization and also serves as the largest immunity organ performing as the first line of defense between the organisms and pathogenic microorganisms [45, 46]. Indeed, the gut microbiota, as a “superorganism” or “forgotten organ” may be a vital mediator for fish health since its perturbations may affect metabolic and physiological functions, thereby contributing partly to the establishment of several injuries and diseases in host [47–50]. From the results obtained, we provide further evidence for the potential application of probiotics in Nile tilapia aquaculture.

## Materials and Methods

### Fish Source, Experimental Procedures, and Feeding

A lot of 150 Apulian naturalized [51] Nile tilapia (*Oreochromis niloticus*) was obtained from Azienda Ittica Agricola Residence San Nazario srls (Lesina, Foggia, Italy). The initial size of 90 days old fish was measured before the experiment and on 96 selected fish of uniform-sized (mean body weight: 41.5 ± 8.3 g) (mean ± SD; *n* = 96) two groups were randomly composed and distributed in two glass tanks (48 fish per tank). In detail, fish were acclimatized under laboratory conditions (water temperature: 24 ± 1 °C; photoperiod: 12-h light/12-h dark) for 2 weeks in glass tanks (tank volume 360 L each) containing constantly aerated water (water volume 300 L each). During this period, fish were fed with a complete commercial diet (Veronesi CFW 4; extruded pellets, 4 mm diameter). Subsequently, fish were washed with sterile filtered water (0.22-µm pore size filters, Millipore), randomly distributed in 6 closed water circuit glass tanks filled with sterile water in a temperature-controlled room (22 °C) and kept without feeding for 48 h to ensure the reduction of pre-existing bacteria. At the end of this period, fish were fed with two different diets as detailed below (see: Experimental Diets section).

### Experimental Diets

Two different diets were used: a control feed and a novel feed (treatment) containing *B. velezensis* MT9 [36]. This bacterial strain was grown in a sterile flask containing GYM as a culture medium (composition per liter: D-glucose 4 g; yeast extract 4 g; malt extract 10 g; agar 15 g when needed). The bacterium was grown at 28 °C with shaking at 200 rpm. The suspension was collected, centrifuged at 3000 rpm for 20 min at 4 °C, washed with 0.9% NaCl solution and centrifuged again. The pellet obtained was resuspended in sterile distilled H_2_O and used to prepare the experimental fish feed. The final concentration of bacteria per gram of feed was 10^6^ CFU/g. The two fish feeds (control and treatment) were prepared from the complete commercial feed described above (i.e., Veronesi CFW 4; extruded pellets, diameter 4 mm). In particular, in order to obtain two comparable diets, the commercial feed was first reduced to a fine powder and then mixed with water (50 mL of distilled H_2_O per 100 g of feed powder) (control diet) or with the *B. velenzensis* MT9 suspension (treatment diet), respectively. Each mass obtained (control or treatment) was coarsely extruded with a metal grinder to obtain a new pellet with a diameter of 1–2 mm. After overnight drying in a desiccator (40 °C), the new feeds were considered ready for use and refrigerated at 6 °C until use. Both the bacterial and experimental feed were prepared weekly. Bacterial survival was assessed by diluting 1 g of feed in 9 mL of 0.9% NaCl and plating serial dilutions onto solid GYM medium. The two diets were isoproteic (35% crude protein), isolipidic (10% crude fat), and isoenergetic (crude fiber 4.80%, ash 5.95%, calcium 0.60%, phosphorus 0.80%, sodium 0.12%). CTL indicated the fishmeal-based control feed, while BV-MT9 indicated the fishmeal-based feed containing *B. velezensis* MT9.

### Fish Feed Trial

The new (BV-MT9) and control (CTL) feed were used for the trial. The study was carried out between June 2023 and September 2023 in experimental tanks at the University of Salento. Six glass tanks (16 fish each) were used as previously described (see: Fish Source, Experimental Procedures, and Feeding section). In particular, three tanks were employed as control, and the fish were nourished with the control feed (CTL), and three tanks were used as treatment, and the fish were nourished with the new feed (BV-MT9). The fish were fed to satiation by hand twice a day, 7 days a week. Temperature and dissolved oxygen were determined daily in the morning and in the afternoon with a digital oximeter (YSI 55 Hexis). At the beginning of the experiment (T0), and then at 30 (T1), 60 (T2), and 90 (T3) days from the beginning of the experiment, three individuals from each tank were singularly collected, anesthetized (0.3% MS- 222, 300 mg/L, Sigma-Aldrich), and weighted by an Analytical Balance Cubis® MSA SARTORIUS (readability 0.01 mg) for growth measurement and biomass gain evaluation. Then, the individuals were washed with sterile filtered water and employed for the subsequent microbiological analyses. At the end of the experimentation trial, survival rate (%), biomass growth, specific growth rate, and coefficient of variation for length were evaluated by using the following equations:

$$\mathrm{Survival}\;\mathrm{rate}\;\left(\%\right)=\left(\mathrm{number}\;\mathrm{of}\;\mathrm{fish}\;\mathrm{at}\;\mathrm{the}\;\mathrm{end}/\mathrm{number}\;\mathrm{of}\;\mathrm{fish}\;\mathrm{at}\;\mathrm{the}\;\mathrm{beginning}\right)\times100$$

$$\mathrm{Biomass}\;\mathrm{gain}\;\left(\mathrm g\right)=\mathrm{final}\;\mathrm{individual}\;\mathrm{weight}-\mathrm{initial}\;\mathrm{individual}\;\mathrm{weight}$$

$$\mathrm{Specific}\;\mathrm{growth}\;\mathrm{rate}\;\left(\mathrm{SGR}\right)\;\left(\%\right)=\left(\ln\;\mathrm{final}\;\mathrm{weight}-\ln\;\mathrm{initial}\;\mathrm{weight}\right)\times100/\mathrm{feeding}\;\mathrm{days}$$

$$\mathrm{Condition}\;\mathrm{factor}\;\left(\mathrm K\right)=\left(\mathrm{weight}\;\mathrm{of}\;\mathrm{the}\;\mathrm{fish}\;\mathrm{in}\;\mathrm{gram}/\left(\mathrm{length}\;\mathrm{of}\;\mathrm{the}\;\mathrm{fish}\;\mathrm{in}\;\mathrm{centimeters}\right)^3\times100\right)$$

Significant statistical differences between the parameters were analyzed using the Student’s *t* test.

### Microbiological Analyses: Culturable Bacteria

Cultural microbiological analyses were performed on both water and fish samples collected in the control and experimental tanks, respectively, at the end of the experimentation period (90 days = T3). Fish collected from each tank were washed with sterile filtered water, and then 70% ethanol was applied to their body surface prior to dissection. Fish were dissected under sterile conditions using individual-use scalpels and forceps. The digestive tract from the stomach to the hindgut was removed intact. Attached organs, such as the liver, were carefully removed. The intestinal tracts of fish from the same tank were merged thus obtaining three different pools (one from each tank) for control and treatment respectively. The pools were aseptically transferred into a sterile stomacher bag, diluted 1:10 with sterile peptone water (1.0 g bacteriological peptone and 8.5 g/L NaCl) and vigorously homogenized for 120 s in a Stomacher 400 Lab Blender (Seward Medical Ltd., 145 UK). The prepared intestine samples were partly used for the evaluation of culturable bacteria and partly frozen at − 80 °C for subsequent DNA extraction. Water samples from each tank were collected aseptically in triplicate by using sterile 1-L bottles. For culture analyses, the water or fish samples serially diluted ten times were immediately added to the appropriate cultural medium. The following microbiological parameters were considered: culturable bacteria at 37 °C, culturable vibrios, total coliforms, fecal coliforms, *Escherichia coli*, *Salmonella* spp., Enterobacteriaceae, *Pseudomonas* spp.

#### Enumeration of Culturable Bacteria at 37 °C

Total culturable bacteria at 37 °C (including human potential pathogens) in the fish (intestine homogenates) and water samples were determined by plating 0.1 mL of each sample and serial dilutions in triplicates on Bacto Plate Count Agar (Difco, Detroit, MI, USA). After incubation for 48 h at 37 °C, the growing CFU were counted.

#### Enumeration of Pollution Indicator Bacteria

To assess the microbial water quality in an easy and reproducible way, standard methods (i.e., ISO—the International Organization for Standardization) were followed. In particular, total coliforms, and fecal coliforms were evaluated by using the most probable number (MPN) method, and the standard five-tube method of tenfold dilutions for water samples [52]. The coliform bacteria concentration was determined by using the miniaturized MPN, in accordance with ISO 9308–3:1998 [53]. Results were referred as MPN/100 mL. The enumeration of *Escherichia coli* was carried out with a five-tube MPN method at three dilutions according to the APAT CNR IRSA 7030 procedures [54]. To count *Salmonella* bacteria, the APAT CNR IRSA 7080 procedure was used [55, 56].

The enumeration of *Escherichia coli* in fish samples was performed by using the MPN method in accordance with the EU reference methods [57, 58]. Briefly, aliquots from each fish diluted homogenate were transferred to tubes with Mineral Modified Glutamate Medium (MMGB) (Oxoid, Basingstoke, UK) [59] by using the standard five-tube method of tenfold dilution. The tubes were incubated aerobically at 37 ± 1 °C for 24 ± 2 h. Positive MMGB tubes changed color from purple to yellow, and subcultures from these tubes were plated on chromogenic Tryptone Bile X-Glucuronide Agar (TBX) plates (Oxoid, Basingstoke, UK) and incubated aerobically at 44 °C for 20 h. At the end of incubation, the grown blue-green colonies were recognized as presumptive *E. coli* [60]. The concentration of *E. coli*/100 g was estimated by counting the number of positive tubes giving the growth of blue-green colonies on TBX agar by using the MPN table reported in ISO 7218:2024 [58].

Coliform bacteria (total and fecal coliforms) concentrations were determined by using the MPN method and the three-tube MPN series following the EU reference methods [61] and Lauryl sulfate tryptose broth (Oxoid, Basingstoke, UK) in the presumptive test (incubation at 37 °C for 24–48 h). All presumptive positive (gas production) tubes were transferred to tubes containing brilliant green lactose bile broth and incubated for 24–48 h at 37 °C (confirmatory test). The number of test tubes giving positive results (gas production) was recorded, and a table for determination of MPN was used.

*Salmonella* spp. were determined following ISO 6579–1:2017/Amd 1:2020 [62]. Briefly, 25 g of each sample were homogenized in 225 mL of buffered peptone water (BPW) (Oxoid, Basingstoke, UK) and incubated for 18 ± 2 h at 37 ± 1 °C. Thereafter, an aliquot of the pre-enrichment was inoculated into two selective broths, Rappaport–Vassiliadis medium with Soya (RVS broth) (Oxoid, Basingstoke, UK) and Muller-Kauffmann Tetrathionate/novobiocin broth (MKTTn broth) (Oxoid, Basingstoke, UK), incubated at 41.5 ± 1 °C for 24 h ± 3 h and 37 ± 1 °C for 24 ± 3 h, respectively. Then, after incubation, sub-cultures from RVS and MKTTn broths were plated onto the surface of one Xylose-Lysine-Desoxycholate (XLD) (Oxoid, Basingstoke, UK) agar plates and incubated at 37 °C for 24 h. Suspected grown colonies were confirmed biochemically (Triple Sugar Iron [TSI] agar, urea agar, L-lysine decarboxylation medium, and indole reaction) and by serological tests.

#### Enumeration of Vibrio

Culturable vibrios were enumerated by filtering 1, 5, and 10 mL of each water or fish (intestine homogenate) sample on 0.45-µm pore size filters (Millipore). Aseptically, filters were placed onto Thiosulphate-Citrate-Bile-Salt-agar (TCBS) plus 2% NaCl, as already reported [63]. Incubation was performed at 22–25 °C for 2 days. After incubation, the colonies of presumptive vibrios (yellow or green), grown on TCBS agar, were counted according to the CFU method. Mean values from three replicates were calculated and expressed as CFU/mL.

#### Enumeration of *Pseudomonas*

For the enumeration of *Pseudomonas* in fish (intestine homogenates) samples, the procedure described in the ISO 13720:2010 [64] was followed. The diluted aliquots (1:10) of the homogenates of the fish samples were seeded for spread plates on Pseudomonas CN Selective Agar [Oxoid SR 102E, suppl. Pseudomonas agar base-(Oxoid CM 0559)] [64] and incubated for 24–48 h at 37 °C. Any colonies with a green–blue color considered *Pseudomonas* positive were subjected to a confirmatory test for oxidase. For the enumeration of *Pseudomonas* in the water samples, the procedure described in ISO 16266:2006 [65] was followed. Briefly, 100 mL of each sample (no dilution, 1:10, 1:100) were filtered through a membrane filter of 0.45 μm and incubated on *pseudomonas* CN selective agar plates, at 37 ± 1 °C for 44 ± 4 h. Green–blue colonies were considered confirmed for *P. aeruginosa* and reported as CFU/100 mL.

### DNA Extraction and 16S rRNA Gene Metabarcoding Analysis of Nile Tilapia Gut Microbiota

As a preliminary step, an initial gut microbiota analysis was performed on control (9 fish intestine homogenates) and treatment (9 fish intestine homogenates) samples, obtained as previously described, to assess the differences between the two groups at the beginning of the experiment (T0) before the start of the feeding. Then, at each sampling time (30 days = T1, 60 days = T2 and 90 days = T3) from the beginning of the experiment, the intestine samples, from control and treatment tanks respectively, were homogenized as previously described, and then, two groups of fish were analyzed and subjected to subsequent DNA extraction: a control group (9 fish at each sampling time) fed with a standard diet (CTL) and a group (9 fish at each sampling time) fed with the standard diet supplemented with BV-MT9. DNA extraction was performed by using the phenol/chloroform protocol, and DNA concentration was measured using UV spectrophotometry (NanoDrop®, ND- 1000 spectrophotometer) [66, 67]. After extraction, DNA was sent to Genomix4 life S.R.L. (Baronissi, Salerno, Italy) for sample quality control, next-generation sequencing, and preliminary bioinformatics analysis. The procedures of sequencing and bioinformatics analysis were previously described [68, 69].

PCR amplification of the V3-V4 hypervariable region of the 16S rRNA gene was performed using the primers: Forward: S-D-Bact- 0341-b-S- 17, 5′-CCTACGGGNGGCWGCAG- 3′, and Reverse: S-D-Bact- 0785-a-A- 21, 5′-GACTACHVGGGTATCTAATCC- 3′ [70]. The assembly of each PCR reaction followed the 16S Metagenomic Sequencing Library Preparation protocol (Illumina, San Diego, CA). Libraries were quantified using a Qubit® 4.0 fluorometer (ThermoFischer Scientific, Waltham, MA, USA) and pooled at an equimolar ratio for each index-tagged sample at a final concentration of 4 nM, including the Phix Control Library (Illumina; 25% expected). Pooled samples were subjected to cluster generation and sequenced on the MiSeq platform (Illumina, San Diego, CA) using a 2 × 250 paired-end format at a final concentration of 10 pM. Taxonomy assignment was performed using Illumina BaseSpace. Bioinformatic processing of the FASTA sequences obtained from sequencing was performed as previously described [68, 69]. Shannon index (α-diversity) was calculated using the pandas library of Python.

### *B. velezensis* MT9: Whole Genome Sequencing, Genome Assembly, and Genome Annotation

Total genomic DNA was extracted from *B. velezensis* MT9 by using the MagAttract® HMW DNA (Qiagen, Hilden, Germany), optimized for the extraction of high molecular weight DNA. The DNA library was prepared using the Ligation Sequencing Kit V14 (SQK-LSK114; Oxford Nanopore Technologies, ONT, Oxford, UK) following the manufacturer instructions. The library was validated using the Fragment Analyzer™ High Sensitivity Genomic DNA Analysis Kit (Agilent Technologies, Santa Clara, CA, USA) and Qubit® 4.0 Fluorometer (ThermoFischer Scientific). The library was finally sequenced onto the ONT MinION platform using the R10.4.1 flow cell for 65 h at the Polo d’Innovazione di Genomica, Genetica e Biologia SRL facility (Polo GGB, Siena, Italy).

Each sequenced sample was trimmed for adapters and barcodes during basecalling, and demultiplexing performed using the tool dorado v0.7.2 (Oxford Nanopore Technologies PLC) in super accurate mode. Quality control was done using the program NanoPlot v1.42.0, and filtering was performed using NanoFilt v2.8.0 and the following thresholds: read length > 200 bp and read quality > 10. The genome assembly step was based on the identification of overlapping regions among the sequencing reads to produce longer sequences representative of genomic regions (contigs). High-quality reads were assembled using the de novo assembler flye v2.9.2 [71]. The completeness and contiguity of the final assemblies were evaluated using BUSCO (v5.7.1, with database bacteria_odb10) [72] based on the evolutionarily informed expectations of gene content from near-universal single-copy orthologs. The quality of the assembly was assessed using the program QUAST 5.2.0 [73]. The annotation step allowed the identification of functional elements along the sequence of a genome. Prokka v1.12 [74] and DFAST v1.2.18 [75] tools were applied to identify features of the sample genomes, including architecture, composition, and functions. Taxonomic identification was performed using the program DFAST_QC [76].

### Bioinformatic Analysis of the Whole Genome of *B. velezensis* MT9

The complete genome sequence of *B. velezensis* MT9 was subjected to bioinformatic analyses by dedicated tools. In particular, the genomic sequence was analyzed using PathogenFinder [77, 78] and VirulenceFinder- 2.0 [79] to identify virulence factors. Genes-encoding proteins involved in antibiotic resistance were analyzed with three bioinformatic tools: Resistance Gene Identifier (CARD) [80], ResFinder 4.0 [81], and ResFinderFG v2.0 [82]. The presence of CRISPR/Cas elements was predicted using CRISPRDetect and CRISPRCasFinder [83, 84]. Mobile genetic elements were identified using PlasmidFinder and MobileElementFinder for plasmids [85, 86], and DBSCAN-SWA and PHASTEST for phages [87, 88]. antiSMASH 7.0 was used to identify potential gene clusters involved in the biosynthesis of secondary metabolites [89].

## Results

### Biometrics of the Nile Tilapia Fed a Diet with or Without *B. velezensis* MT9

**Fig. 1:**
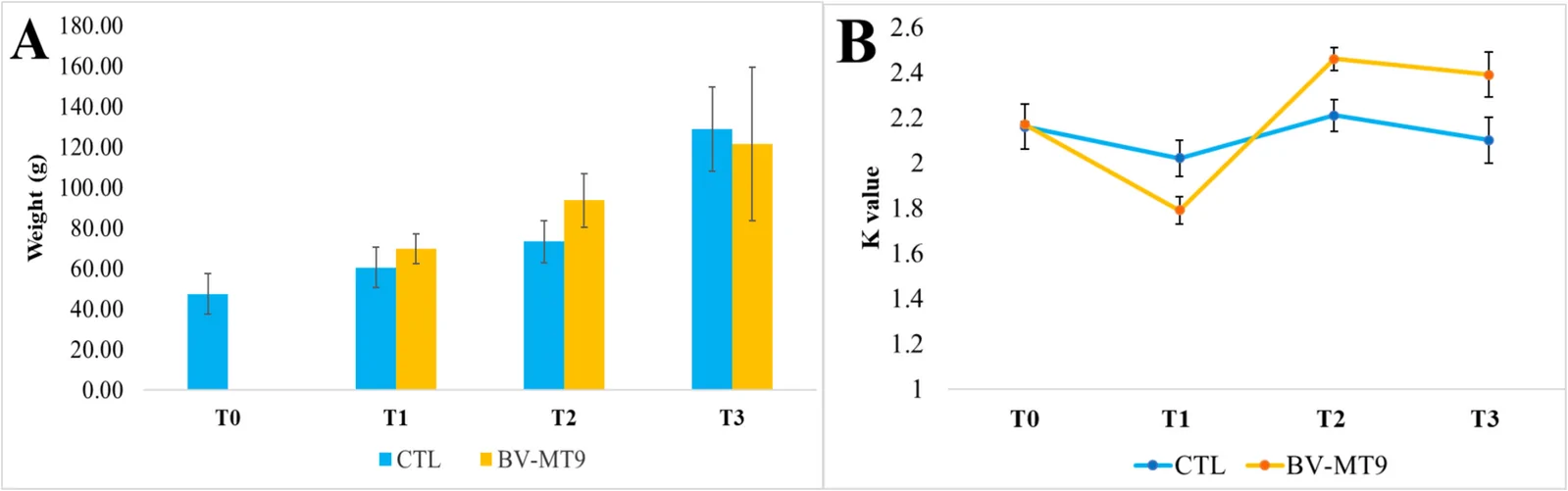
Fish weight (**A**) and condition factor (*K* value) (**B**) in the control (CTL) and treatment (BV-MT9) samples during the different sampling times (T0 = beginning of the experiment, T1 = 30 days, T2 = 60 days, and T3 = 90 days from the beginning of the experiment). Data represent mean values ± standard error

During the diet administration experiment, the following parameters were measured: survival rate (%), biomass gain, specific growth rate (SGR), and condition factor (*K*). Fish fed with either the control diet (CTL) or the *Bacillus velezensis* MT9-enriched feed (BV-MT9) showed 100% survival in both groups at each sampling time. Data concerning fish weight at the different sample times examined are reported in Fig. 1A. At the end of the experiment, the biomass gain was 81.47 g for the CTL group and 74.27 g for the BV-MT9 group. The SGR was slightly higher in the CTL group (0.83%) compared to the BV-MT9 group (0.78%). Conversely, the *K* index was higher in the BV-MT9 group (2.39) than in the CTL group (2.10) (Fig. 1B). However, the observed differences in all analyzed parameters were statistically not significant (*p* value > 0.05).

### Microbiological Analyses: Culturable Bacteria

Several microbiological parameters were monitored in both water and fish samples collected in the control and experimental tanks, respectively, at the end of the experimentation period (90 days). Table 1 shows the results of microbiological analysis on the water samples as regards culturable vibrios, total coliforms, fecal coliforms, *Escherichia coli*, *Salmonella*, *Staphylococcus*, and *Pseudomonas* species in the control and experimental tanks collected at the end of the experimentation period.

**Table 1:** Total coliforms, fecal coliforms, *Escherichia coli* (MPN/100 mL), *Salmonella* spp. (presence/absence), Staphylococci, *Pseudomonas* spp., and culturable vibrios (CFU/mL) in water samples collected from tanks containing tilapia fed with conventional feed or tilapia fed with probiotic feed

	Total coliforms	Fecal coliforms	*E. coli*	*Salmonella* spp.	*Staphylococci Coag.-pos*	*Pseudomonas* spp.	Vibrios
	MPN/100 mL	MPN/100 mL	MPN/100 mL	Presence/absence	CFU/mL	CFU/mL	CFU/mL
Water control	2419.6 ± 62.0	0	< 1	Absent	0	0	0
Water + probiotic	260.3 ± 5.0	0	< 1	Absent	0	0	0

In particular, total coliforms reached the mean value of 2419.6 ± 62.0 MPN/100 mL in the control water (CTL) and 260.3 ± 5.0 MPN/100 mL in the water of the tanks in which the organisms were fed with BV-MT9. *E. coli* resulted in < 1 MPN/100 mL in both the control and the BV-MT9 treatment. *Salmonella*, *Staphylococcus*, *Pseudomonas* species, and culturable vibrios were absent in all the water samples from the six tanks analyzed. The results showed that the concentration of culturable bacteria at 37 °C was lower in the water in which fish fed with *B. velezensis*-enriched feed were raised than in the control water samples examined.

**Fig. 2:**
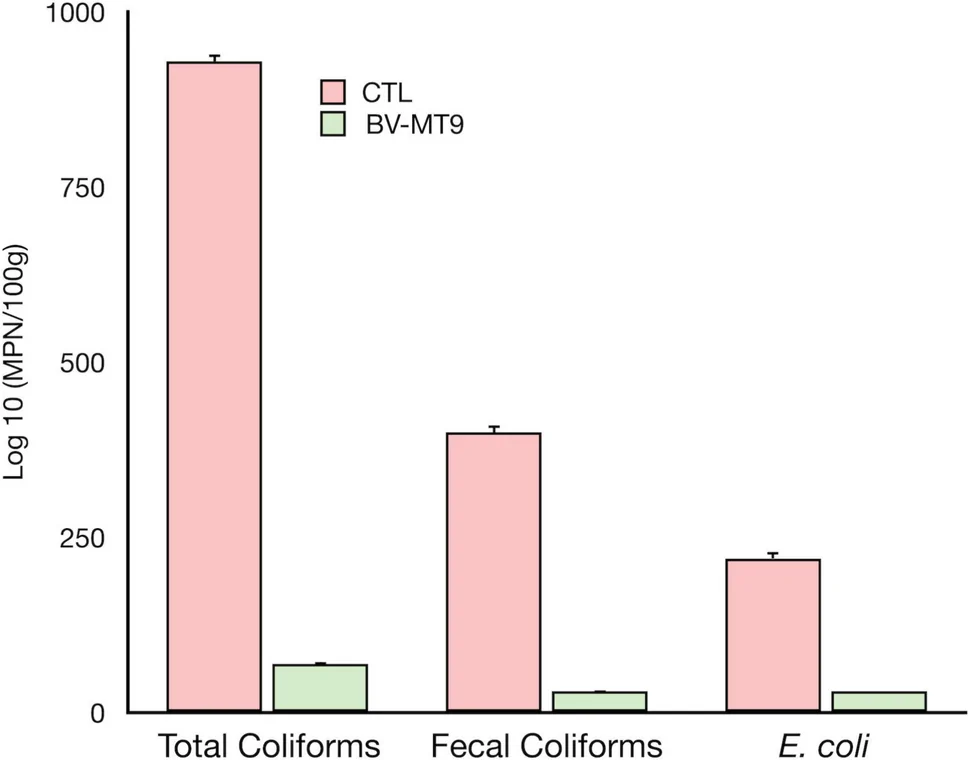
Concentrations of total coliforms, fecal coliforms, and *Escherichia coli* in the intestine of both tilapias fed with conventional feed (CTL = control) and fed with the probiotic *B. velezensis* MT9-supplemented feed (BV-MT9). Data represent mean values ± standard error

Figure 2 shows the results of total coliforms, fecal coliforms, and *E. coli* densities in the intestine of fish fed with conventional feed as well as in the intestine of fish fed with the *B. velezensis* supplemented feed.

In particular, total coliform mean density was 930 ± 9 MPN/100 g in the intestine of fish fed with conventional feed, while in fish nourished with *B. velezensis* MT9-enriched feed the mean density of these microorganisms was 70 ± 2 MPN/100 g. Fecal coliform concentration in the intestine of control tilapia was 400 ± 10 MPN/100 g, while these bacteria decreased to 30.0 ± 1.5 MPN/100 g in the *B. velezensis* nourished tilapia. *E. coli* reached the mean value of 220.0 ± 9.4 MPN/100 g and 30.0 ± 0.5 MPN/100 g in the intestine of control and treated tilapia, respectively.

**Fig. 3:**
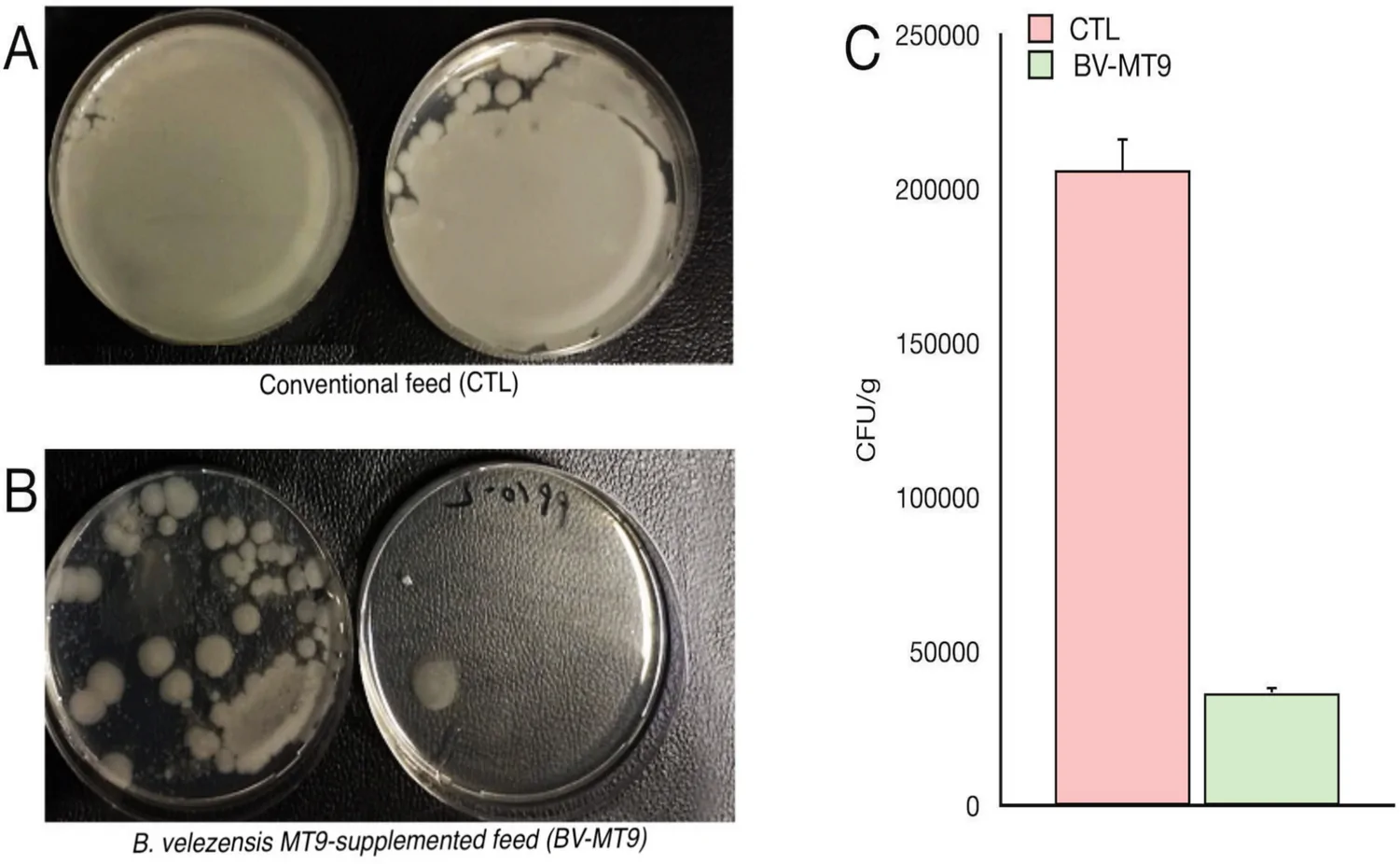
Petri dishes with plate count agar indicating the growth of mesophilic bacteria at 37 °C in the intestine of both tilapias fed with conventional feed (**A**) and fed with the probiotic *B. velezensis* MT9-supplemented feed (BV-MT9) (**B**). Concentrations of mesophilic bacteria in both control CTL and treatment = BV-MT9 samples (**C**). Data represents mean values ± standard error

As shown in Fig. 3, the microbiological concentration due to mesophilic bacteria at 37 °C was higher (2.06 ± 0.37 × 10^5^ CFU/g) in the intestine of the control farmed fish compared to the intestine of the fish fed with *B. velezensis*-based feed (3.65 ± 0.31 × 10^4^ CFU/g).

Finally, at the end of the experimental period (Table 2), *Salmonella* spp., Staphylococci, *Pseudomonas* spp., and culturable vibrio resulted absent in all the examined intestine samples.

**Table 2:** Microbiological parameters (*Salmonella* spp*.*, Staphylococci, *Pseudomonas* spp., and vibrios) in the intestine of both tilapias fed with conventional feed and fed with the probiotic-supplemented feed

	*Salmonella* spp*.*	Staphylococci	*Pseudomonas* spp.	Vibrios
	Presence/absence	CFU/g	Presence/absence	CFU/g
Intestine control	Absent	0	Absent	0
Intestine + probiotic	Absent	0	Absent	0

### *B. velezensis* MT9 Modulates the Intestinal Microbiota of Nile Tilapia: Overview

To analyze the effects of the dietary supplementation with *B. velezensis* MT9 on the modulation of the gut microbial community of Nile tilapia, a 16S rRNA gene metabarcoding approach was used (File S1 and File S2).

The diversity analysis of the Shannon index highlighted that both groups (CTL and BVMT9) presented a similar α-diversity at the beginning of the experiment (T0), analyzing both the genera and the families (Table 3).

**Table 3:** α-diversity: Shannon diversity index determined for families and genera at the different considered times (T0, T1 = 30 days, T2 = 60 days, and T3 = 90 days)

	Sample	T0	T1	T2	T3
Families	CTL	2.940	1.342	1.511	2.584
	BV-MT9	2.662	0.789	1.596	1.918
	Water CTL				2.857
	Water BV-MT9				2.932
Genera	CTL	3.137	1.451	1.609	3.354
	BV-MT9	2.925	0.829	1.669	2.038
	Water CTL				4.108
	Water BV-MT9				4.098

Analyzing the microbiota composition in the two groups at the beginning of the experiment, no significant differences were observed (File S1).

The analysis of the Shannon diversity index at the level of families and genera demonstrates an increase in the α-diversity during the time course of the experiment (T1, T2, T3) both in the control group of Nile tilapia (CTL) and in the group treated with BV-MT9 (Table 3). However, compared to CTL, dietary supplementation with BV-MT9 resulted in a decrease of the α-diversity at the time points T1 and T3. In contrast, Shannon diversity index at the family and genus levels did not change substantially in water samples from tanks with *B. velezensis* MT9-treated or untreated Nile tilapia (Table 3).

**Fig. 4:**
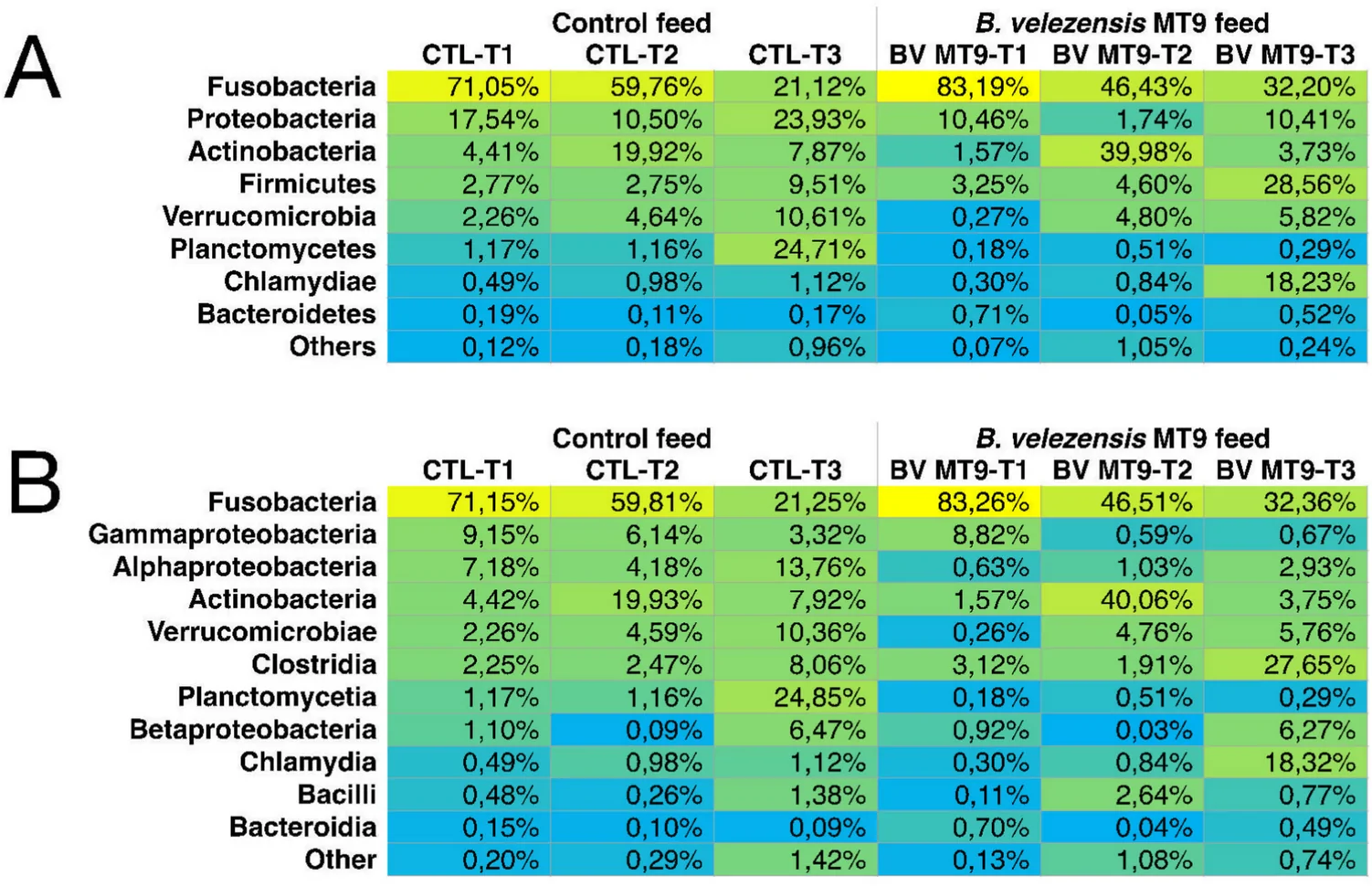
Analysis of the gut microbial community of Nile tilapia at phylum (**A**) and class levels (**B**) in the control (CTL) and treatment (BV MT9)

At the phylum level, the gut bacterial community of the CTL group of Nile tilapia was dominated by Fusobacteria (Fusobacteriota), whose relative abundance within each sample, however, decreased during the time course (T1 to T3) both in the CTL group (from 71.05 to 21.12%) and in the group treated with BV-MT9 (from 83.19 to 32.20%) (Fig. 4A).

Other major components of the gut bacterial community with relative within sample abundance > 0.1% in at least one sample were Proteobacteria (Pseudomonadota), Actinobacteria (Actinomycetota), Firmicutes (Bacillota), Verrucomicrobia (Verrucomicrobiota), Planctomycetes (Planctomycetota), Chlamydiae (Chlamydiota), Bacteroidetes (Bacteroidota), Acidobacteria (Acidobacteriota), and Cyanobacteria (Cyanobacteriota)/Chloroplast.

An opposite trend was observed between Proteobacteria and Actinobacteria. The relative abundance of Proteobacteria decreased at T2 increased at T3, while that of Actinobacteria increased at T2 and decreased at T3. The decrease in the relative abundance of Proteobacteria at T2 was much more pronounced in the BV-MT9 group (from 10.46 in T1 to 1.74% in T2) compared to the CTL group (from 17.54 in T1 to 10.50% in T2). In parallel, the increase in the relative abundance of Actinobacteria at T2 was much more pronounced in the BV-MT9 group (from 1.57 in T1 to 39.98% in T2) compared to the CTL group (from 4.41 in T1 to 19.92% in T2). It can be noted that Proteobacteria were less abundant in the BV-MT9 group than in the CTL group at the corresponding time points. In contrast, Firmicutes, whose abundance increased from T1 to T3, were more represented in the BV-MT9 group compared to the CTL group, and at T3 reached a relative abundance of 28.56% in the BV-MT9 group compared to 9.51% in the CTL group.

Regarding the other phyla: Verrucomicrobia, whose abundance also increased from T1 to T3, were less represented in the BV-MT9 group (0.27% at T1; 4.80% at T2; 5.82% at T3) compared to the CTL group (2.26% at T1; 4.64% at T2; 10.61% at T3); the abundance of Planctomycetes, which tended to remain low and almost stable over time, showed a dramatic increase in the CTL group at T3 reaching a relative abundance of 24.71%; the abundance of Chlamydiae, which was also low and almost stable over time, substantially increased in the BV-MT9 group at T3 reaching a relative abundance of 18.23%; Bacteroidetes, Acidobacteria, and Cyanobacteria/Chloroplast did not show clear distinctive trends in the two groups of fish.

### *B. Velezensis* MT9 Reduces the Amount of Opportunistic Pathogens and Increases the Amount of Beneficial Bacteria

**Fig. 5:**
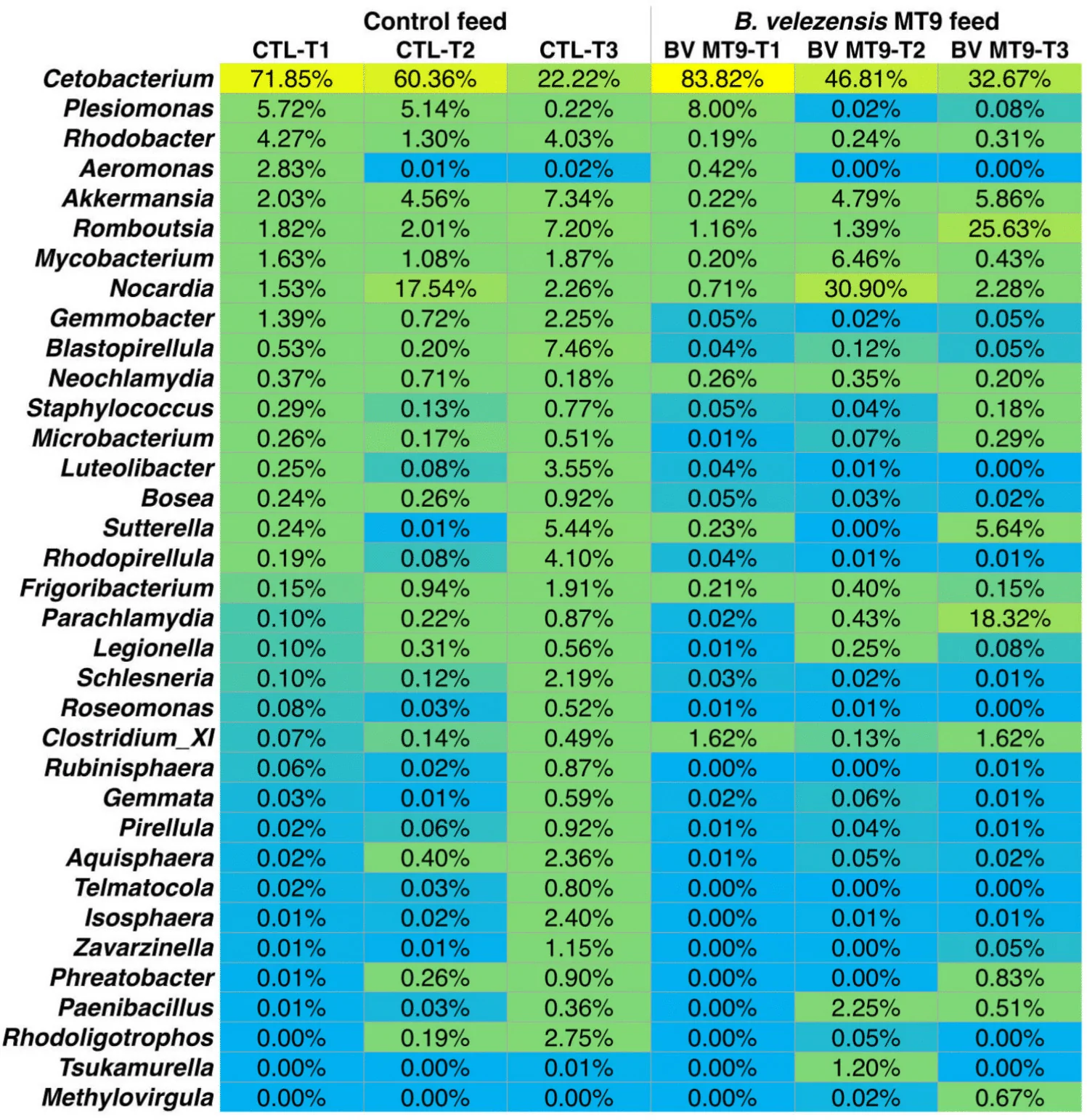
Analysis of the gut microbial community of Nile tilapia at genus level in the control (CTL) and treatment (BV MT9)

The differences found at the phyla level between the two groups of fish were reflected in the differences found at the level of class (Fig. 4B), family (Fig. S1), and genus (Fig. 5).

At the genus level, the gut bacterial community of the Nile tilapia was dominated by *Cetobacterium* (Fusobacteriota; Fusobacteriia; Fusobacteriaceae), whose relative abundance within each sample, however, decreased during the time course (T1 to T3) both in the CTL group of Nile tilapia (from 71.85 to 22.22%) and in the BV-MT9 group (from 83.82 to 32.67%) (Fig. 5). It may be noted the greater relative abundance of the genus *Cetobacterium* in the BV-MT9 group compared to the CTL group (Fig. 5).

In the BV-MT9 group compared to the CTL group, it may be also noted the greater relative abundance of the genus *Romboutsia* (Bacillota; Clostridia; Peptostreptococcales; Peptostreptococcaceae) at T3 (25.63% in BV-MT9; 7.20% in CTL) (Fig. 5).

The analysis of the gut bacterial community of the Nile tilapia at the genus level also revealed that treatment with *B. velezensis* MT9 resulted in a notable decrease in the relative abundance of bacteria belonging to the genus *Aeromonas* (Pseudomonadota; Gammaproteobacteria; Aeromonadales; Aeromonadaceae). In particular, at T3, this genus was no longer detectable in the BV-MT9 group (Fig. 5). In addition to the *Aeromonas*, treatment with *B. velezensis* MT9 resulted in a decrease in the relative abundance of bacteria belonging to the *Vibrio* (Pseudomonadota; Gammaproteobacteria; Vibrionales; Vibrionaceae) and *Escherichia/Shigella* (Pseudomonadota; Gammaproteobacteria; Enterobacterales; Enterobacteriaceae) genera, which also include members pathogenic to fish (Fig. 5). Although these genera had low relative abundance values, their decrease was also experimentally demonstrated as discussed above.

At T3, we also noticed an increase in the relative amount of *Parachlamydia* (Chlamydiota; Chlamidiia; Parachlamydiales) in the BV-MT9 group (Fig. 5). This result could be indicative for a weakness of the dietary supplementation with *B. velezensis* MT9, because a member of this genus, *Parachlamydia acanthamoebae*, is an amoeba-resistant microorganism that has evolved to survive and multiply within these protozoa, and its pathogenic role towards humans or animals is suspected [90].

The analysis of the intestinal bacterial community at the genus level also revealed that the increase in the relative abundance of Actinobacteria at T2, which was much more pronounced in the BV-MT9 group compared to the CTL group, was mostly due to members of the genera *Nocardia* (Actinomycetota; Actinomycetes; Mycobacteriales; Nocardiaceae) (17.54% in CTL; 30.90% in BV-MT9 at T2), and *Mycobacterium* (Actinomycetota; Actinomycetes; Mycobacteriales; Mycobacteriaceae) (1.08% in CTL; 6.46% in BV-MT9 at T2). Regarding genera with *B. velezensis* activity, the analysis of the gut bacterial community revealed the notable increase in the relative amount of *Paenibacillus* (Bacillota; Bacilli, Bacillales; Paenibacillaceae) in the Nile tilapia with dietary supplementation with *B. velezensis* MT9, particularly at T2 (0.03% in CTL; 2.25% in BV-MT9 at T2).

Overall, the analysis of the microbial community of the Nile tilapia demonstrates that *B. velezensis* MT9 reshapes the intestinal microbiota of Nile tilapia by reducing the amount of several opportunistic Gram-negative bacterial pathogens belonging to the phylum of Proteobacterium (Pseudomonadota) and increasing the amount of several beneficial bacteria belonging to the phyla Firmicutes (Bacillota) and Actinobacteria (Actinomycetota). The analysis of the gut bacterial community at the genus level also revealed that the diet supplementation of *B. velezensis* MT9 did not result in a substantial increase in the relative amounts of bacteria of the genus *Bacillus*, except a transient increase at T2 (0.06% in CTL; 0.31% in BV-MT9 at T2).

### *B. Velezensis* MT9 Modulates the Water Microbiota

The analysis of the microbial community at the phyla level shows an effect of the dietary supplementation of *B. velezensis* MT9 on the modulation of the water microbiota (Fig. S2A), with a reduction in the relative abundance of Planctomycetes (Planctomycetota) (25.95% in BV-MT9; 35.80% in CTL), Actinobacteria (Actinomycetota) (2.96% in BV-MT9; 4.45% in CTL), and Chloroflexi (Chloroflexota) (1.84% in BV-MT9; 3.19% in CTL). In contrast, in the water from tanks of Nile tilapia supplemented with *B. velezensis* MT9, the relative abundance of the following phyla was increased: Bacteroidetes (Bacteroidota) (13.90% in BV-MT9; 9.22% in CTL), Candidatus Parcubacteria (0.93% in BV-MT9; 0.26% in CTL), and Candidate Division WPS- 1 (3.50% in BV-MT9; 0.02% in CTL). From these data, we can see the increase in the relative abundance of bacteria belonging to unclassified candidate phyla in the water from Nile tilapia tanks enriched with *B. velezensis* MT9.

The differences found at the phyla level between the two groups of fish were reflected in the differences found at the level of class (Fig. S2B), family (Fig. S3), and genus (Fig. S4). At the genus level, the water community of the Nile tilapia tanks was dominated by *Rubinisphaera* (Planctomycetota; Planctomycetia; Planctomycetales; Plancomycetaceae), whose relative abundance was decreased in the water of the BV-MT9 group compared to the CTL group (9.21% in BV-MT9; 17.35% in CTL). Furthermore, the relative abundance of the following genera was decreased in the water of the BV-MT9 group: *Parachlamydia* (Chlamydiota; Chlamydiia; Parachlamydiales; Parachlamydiaceae) (1.13% in BV-MT9; 8.28% in CTL), *Planctomicrobium* (Planctomycetota; Planctomycetia; Planctomycetales; Plancomycetaceae) (2.83% in BV-MT9; 5.20% in CTL), *Schlesneria* (Planctomycetota; Planctomycetia; Planctomycetales; Plancomycetaceae) (0.26% in BV-MT9; 4.05% in CTL), *Telmatocola* (Planctomycetota; Planctomycetia; Gemmatales; Gemmataceae) (0.03% in BV-MT9; 1.78% in CTL), *Chryseolinea* (Bacteroidota; Cytophagia; Cytophagales; Fulvivirgaceae) (0.08% in BV-MT9; 1.05% in CTL), *Tepidisphaera* (Planctomycetota; Phycisphaerae; Tepidisphaerales; Tepidisphaeraceae) (0.07% in BV-MT9; 1.03% in CTL). Other genera, including *Rheinheimera*, *Caldilinea*, *Haliea*, and *Labilithrix*, with relative abundance values below 1% were significantly more abundant in the CTL sample. By contrast, the relative abundance of the following genera was increased in the water of the BV-MT9 group: *Flavobacterium* (Bacteroidota; Flavobacteriia; Flavobnacteriales; Flavobacteriaceae) (8.83% in BV-MT9; 3.50% in CTL), *Spartobacteria*_gen._incertae_sedis (Verrucomicrobiota) (2.25% in BV-MT9; 1.47% in CTL), *Rhodoligotrophos* (Pseudomonadota; Alphaproteobacteria; Hyphomicrobiales; Rhodoligotrophaceae) (2.23% in BV-MT9; 0.82% in CTL), *Frigoribacterium* (Actinomycetota; Actinomycetes; Micrococcales; Microbacteriaceae) (2.03% in BV-MT9; 0.58% in CTL), *Novosphingobium* (Pseudomonadota; Alphaproteobacteria; Sphingomonadales; Sphingomonadaceae) (3.61% in BV-MT9; 0.55% in CTL), *Planctopirus* (Planctomycetota; Planctomycetia; Planctomycetales; Plancomycetaceae) (8.22% in BV-MT9; 0.43% in CTL), *Neochlamydia* (Chlamydiota; Chlamydiia; Parachlamydiales; Parachlamydiaceae) (6.30% in BV-MT9; 0.11% in CTL), Candidate Division WPS- 1 genus (3.90% in BV-MT9; 0.02% in CTL). In summary, from these data we can see a different distribution of genera within the Planctomycetota and Chlamydiota phylum between the BV-MT9 and the CTL groups.

### The Beneficial Effects of *B. velezensis* MT9 are Revealed by Comparative Whole Genome Sequence Analysis

The genome of *B. velezensis* MT9 was determined by MinIon Oxford Nanopore Technologies platform. The whole genome sequencing yielded a total of 3,689,740 reads with a read length N50 of 2,870. Mean and median read length were, respectively, 2,332.90 and 2,275.00; mean and median read qualities were, respectively, 19.3 and 23.4. The assembled genome resulted in a single contig with a total length of 4,139,342 bp and GC% of 46.5 (Table S1). The benchmarking universal single-copy orthologs (BUSCO) confirmed the completeness of the assembly (Table S2). Regarding coding sequences (CDS), RNAs and tRNAs, both Prokka [74] and DFAST [62] tools predicted similar numbers of entries (Table 4).

**Table 4:** Genome annotation metrics obtained using Prokka and DFAST for the single-contig genome of *B. velezensis* MT9. Key features (total sequence length, number of coding sequences (CDSs), rRNAs, tRNAs, and additional annotations like CRISPRs number and GC content) are highlighted

**Prokka**	
Contigs	1
Total sequence length (bp)	4,139,342
Number of CDSs	3995
Number of rRNAs	27
Repeat_region	NA
Number of tRNAs	86
Number of tmRNAs	1
**DFAST**	
Contigs	1
Total sequence length (bp)	4,139,342
Number of CDSs	3997
Average protein length	305.1
Number of rRNAs	27
Number of tRNAs	86
Number of CRISPRs	0
Longest sequences	4,139,342
N50 (bp)	4,139,342
Coding ratio (%)	88.4%
Gap ratio (%)	0.00%
GCcontent (%)	46.1%

Whole genome sequencing (WGS)-based taxonomic identification was performed using DFAST_QC algorithm which are based on: NCBI assembly database plus the ANI report and on the Genome Taxonomy Database (GTDB). Alignment with the public databases confirmed that the strain MT9 belongs to the species to *B. velezensis* with an average nucleotide identity (ANI) of 99.5% with *B. velezensis* reference strains KCTC 13012 and NRRL B- 41580 (Table S3 and Table S4). PlasmidFinder 2.0 [85] and MobileElementFinder [86] did not find any plasmid sequence.

The phage-associated proteins were analyzed using DBSCAN-SWA [87], and PHASTEST [88] tools. PHASTEST identified three regions that contained phage proteins. Region 1 (coordinates: 1,626,138–1,702,700) and Region 3 (coordinates: 2,095,095–2147716) showed a score > 90 and were annotated as “intact”. DBSCAN-SWA confirmed that these two regions contained proteins belonging to the phage *Bacillus* SPBc2. Region 2 annotated by PHASTEST (coordinates: 1,698,139–1,779,328) had a score of 70 and was annotated as questionable. Again, DBSCAN-SWA confirmed the presence of phage sequences derived from bacteriophage phi- 105 in this region. Finally, DBSCAN-SWA predicted that an additional genomic region (coordinates 3,235,093–3240999) contained the complete sequence of an entomopoxvirus described in the insect *Anomala cuprea*.

Regarding resistance genes, the tool resistance gene identifier (RGI) [80] found eight strict matches dealing with glycopeptide antibiotic resistance and efflux pumps (Fig. S8), while ResFinder- 4.6.0 [81], and ResFinderFG- 2.0 [82] did not find any match. Virulence and pathogenicity were assessed using the tools VirulenceFinder- 2.0 [79] and PathogenFinder [77, 78]. VirulenceFinder was run using various reference organisms (*Staphylococcus aureus*, *Enterococcus*, *E. coli*, *Listeria*) but did not identify any virulence factors. PathogenFinder predicted a very low probability that the bacterium is a pathogen (score 0.112) and did not identify any pathogenicity factors. The presence of CRISPR/Cas elements in the genome was assessed using two different tools: CRISPRDetect [83] and CRISPRCasFinder [84]. CRISPRDetect did not detect any of these elements. CRISPRCasFinder using the “alternative detection of truncated repeats results level 1” option identified short elements that with high probability do not correspond to CRISPRs (evidence level 1).

The Kyoto Encyclopedia of Genes and Genomes (KEGG) pathway database was used for genome-based metabolic reconstruction of *B. velezensis* MT9 and comparative analysis of pathways in *B. velezensis* MT9 and ten *B. velezensis* reference strains (B19, 12Y, XRD006, Pilsner 1–2, CGMCC, AP46, 160, A03, PEBA20, GJJK74) (File S3). The analysis predicted a total of 599 metabolic pathways in *B. velezensis* MT9 and a similar number ranging from 598 and 602 in the other strains (File S3). Considering the KEGG pathways with variations > 1 between strains in number of entries, the following are those with greater diversity in *B. velezensis* MT9 compared to the reference strains: glycolysis/gluconeogenesis; starch and sucrose metabolism; amino sugar and nucleotide sugar metabolism; sulfur metabolism; pyrimidine metabolism; cysteine and methionine metabolism; O-antigen nucleotide sugar biosynthesis; teichoic acid biosynthesis; one carbon pool by folate; benzoate degradation; nitrotoluene degradation; ribosome; sulfur relay system; homologous recombination; ABC transporters; phosphotransferase systems; two-component systems; quorum sensing; biofilm formation (Table 5). For each of these KEGG pathway, comparative KEGG maps were generated to highlight specific traits of *B. velezensis* MT9 (Fig. S5-S15). Comparative analysis demonstrated that the different strains of *B. velezensis* exhibit differences in some pathways, which could be relevant for their use as probiotics. *B. velezensis* MT9 is one of the strains with greater metabolic capabilities for the pathways: starch and sucrose metabolism (Fig. S6); amino sugar and nucleotide sugar metabolism (Fig. S7); ABC transporters (Fig. S8). The analysis, however, revealed the lack of the ProV component of the proline/glycine/betaine transporter (ProX/ProW/ProV) only in *B. velezensis* MT9. In *B. velezensis* MT9 and also in other strains, the analysis showed the absence of the following: (i) BglF, a phosphotransferase system (PTS) involved in α-glucoside transport (Fig. S9); (ii) the thymidylate synthase (EC 2.1.1.148) involved in one carbon pool by folate metabolism (Fig. S10); and pyrimidine metabolism (Fig. S11); RuvC of the RuvA-RuvB-RuvC complex that processes Holliday junction DNA during genetic recombination and DNA repair (Fig. S12).

**Table 5:** Comparative analysis of KEGG pathways in *B. velezensis* MT9 and reference *B. velezensis* strains

**Strain**	MT9	B19	12Y	XRD006	Pilsner 1–2	CGMCC	AP46	160	A03	PEBA20	GJJK74
Metabolism											
*Carbohydrate metabolism*											
Glycolysis/gluconeogenesis	30	30	29	29	29	29	30	30	30	30	30
Starch and sucrose metabolism	27	27	26	26	26	26	24	24	27	27	24
Amino sugar and nucleotide sugar metabolism	29	29	28	28	28	28	32	31	29	29	31
*Energy metabolism*											
Sulfur metabolism	15	16	16	16	16	16	15	15	16	15	15
*Nucleotide metabolism*											
Pyrimidine metabolism	6	6	6	6	6	6	6	5	6	6	6
*Amino acid metabolism*											
Cysteine and methionine metabolism	39	41	39	39	39	39	40	40	41	39	39
*Glycan biosynthesis and metabolism*											
O-Antigen nucleotide sugar biosynthesis	13	13	13	13	13	13	16	15	13	13	15
Teichoic acid biosynthesis	16	16	16	16	16	16	16	15	15	16	15
*Metabolism of cofactors and vitamins*											
One carbon pool by folate	11	11	12	12	12	12	11	11	11	12	11
*Xenobiotics biodegradation and metabolism*											
Benzoate degradation	7	7	7	7	7	7	8	8	7	7	8
Nitrotoluene degradation	0	1	0	0	0	0	0	0	1	0	0
Genetic information processing											
*Translation*											
Ribosome	54	55	55	55	55	55	55	55	55	55	55
*Folding*, *sorting*, *and degradation*											
Sulfur relay system	13	13	13	13	13	13	12	13	13	13	12
*Replication and repair*											
Homologous recombination	18	18	19	19	19	19	18	17	18	19	18
Environmental information processing											
*Membrane transport*											
ABC transporters	101	96	97	97	95	97	90	89	99	101	90
Phosphotransferase systems	18	18	17	17	17	17	19	18	18	18	19
*Signal transduction*											
Two-component systems	105	104	107	107	105	107	106	105	107	105	105
Cellular processes											
*Cellular community—prokaryotes*											
Quorum sensing	52	52	54	54	54	54	55	51	54	52	54
Biofilm formation – Vibrio cholerae	8	8	7	7	7	7	8	8	8	8	8
Biofilm formation – *Escherichia coli*	5	5	4	4	4	4	5	5	5	5	5

**Fig. 6:**
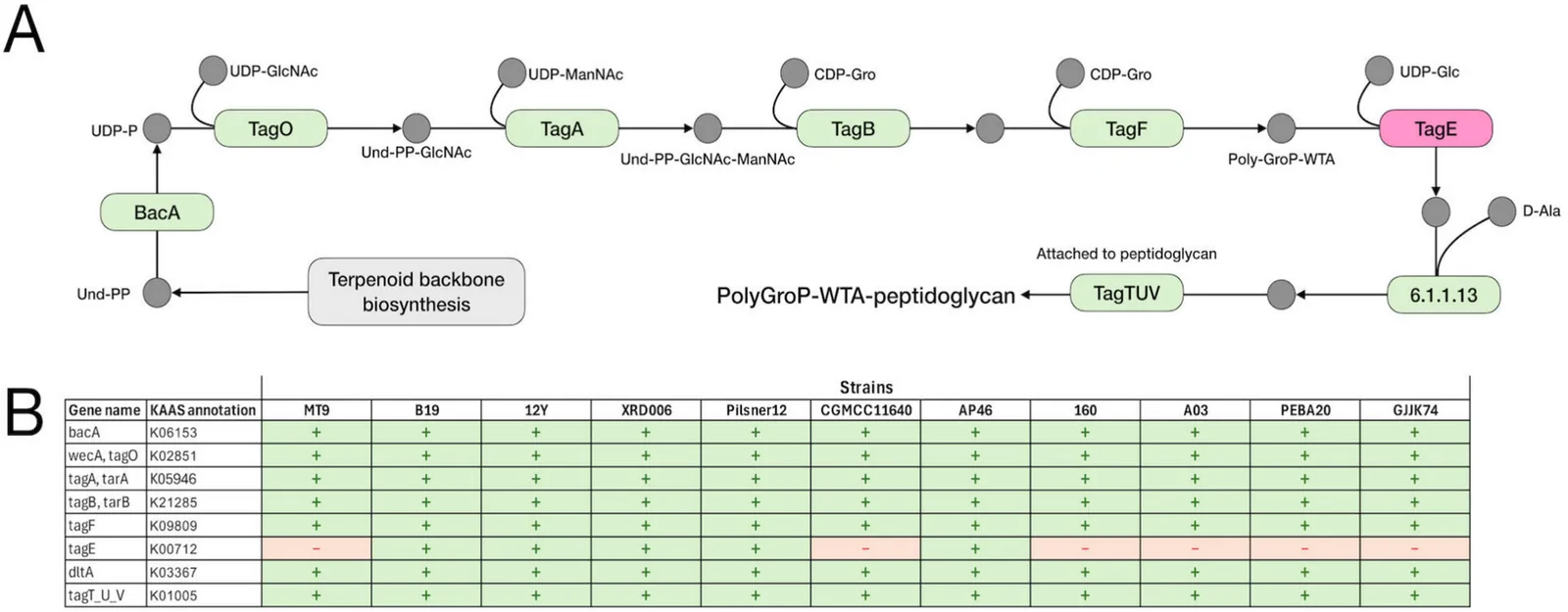
Differences between *B. velezensis* MT9 and ten *B. velezensis* reference strains in wall teichoic acid biosynthesis as predicted by comparative KEGG analysis. Reconstruction of KEGG pathway (map00552) (**A**). Genes involved in wall teichoic acid biosynthesis in *B. velezensis* MT9 and ten *B. velezensis* reference strains (**B**)

Intriguingly, the comparative genomic analysis showed the absence in *B. velezensis* MT9 of *rpmH*, the gene encoding the L34 (Fig. S13). L34 is a large ribosomal subunit protein that is nonessential for survival of bacteria under definite conditions [91, 92]. The KEGG analysis also predicted differences in wall teichoic acid (WTA) biosynthesis among the different *B. velezensis* strains. Specifically, the analysis demonstrated that *tagE*, coding for the WTA glycosyltransferase *TagE*, is absent in MT9, CGMCC11640, 160, A03, PEBA20, and GJJK74 (Fig. 6).

*TagE* catalyzes the transfer of alpha-glucose from UDP-glucose onto position 2 of the poly(glycerol phosphate) polymer, and is responsible for WTA glycosylation in *B. subtilis* 168 [93].

**Fig. 7:**
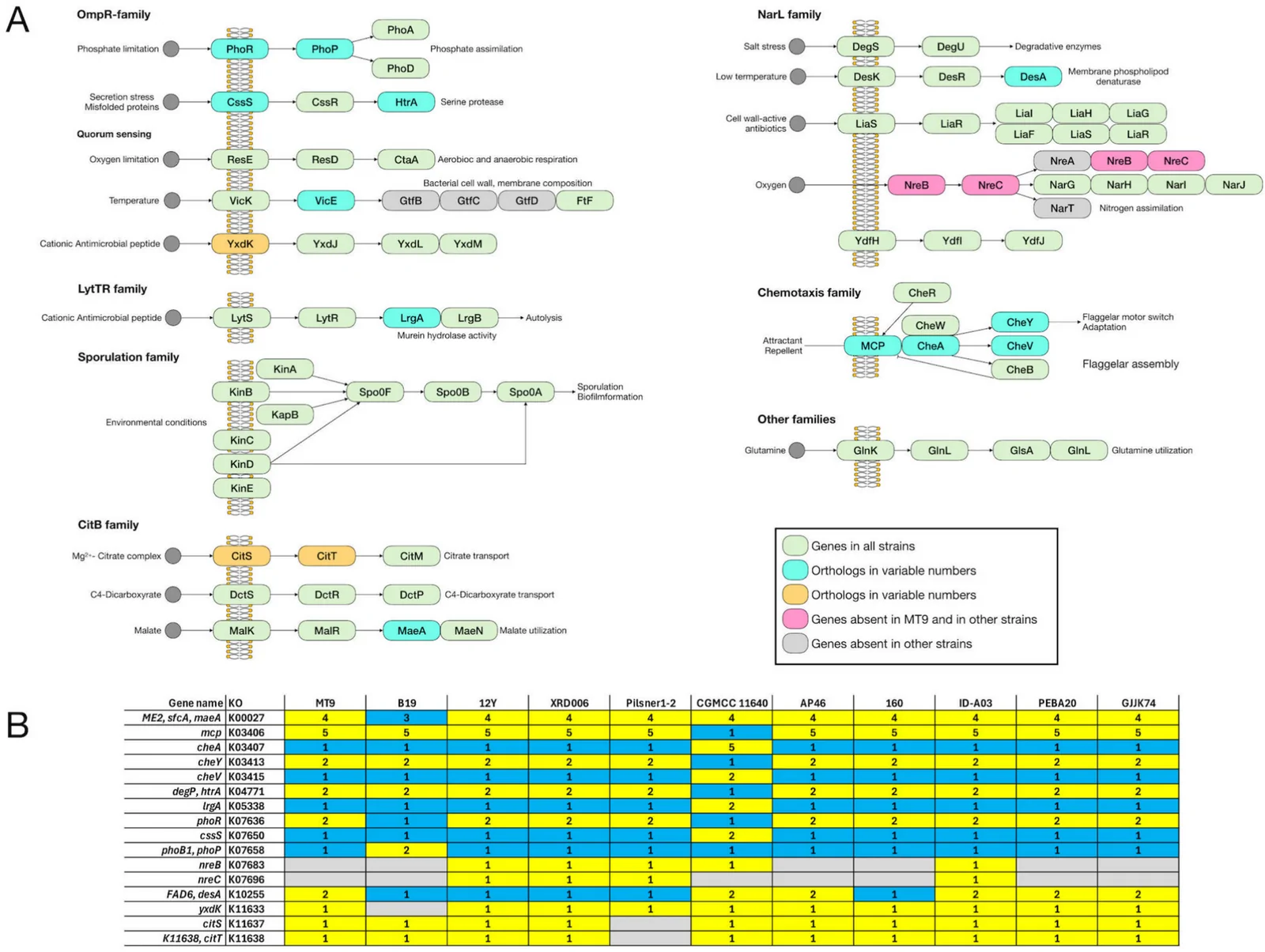
Differences between *B. velezensis* MT9 and ten *B. velezensis* reference strains in two-component systems as predicted by comparative KEGG analysis (map02020). Reconstruction of KEGG pathway (**A**). Two-component systems and corresponding genes in *B. velezensis* MT9 and ten *B. velezensis* reference strains (**B**)

The comparative genomic analysis also revealed that the different strains of *B. velezensis* exhibit differences in two-component systems and quorum sensing. As regards the two-component systems, the genes coding for the peptide pheromone ComX, which is required for the development of genetic competence in *B. subtilis* [94], and NreB and NreC, which are involved in O_2_ and nitrate sensing [95], are absent in *B. velezensis* MT9 and in several *B. velezensis* strains (Fig. 7 and Fig. S13).

**Fig. 8:**
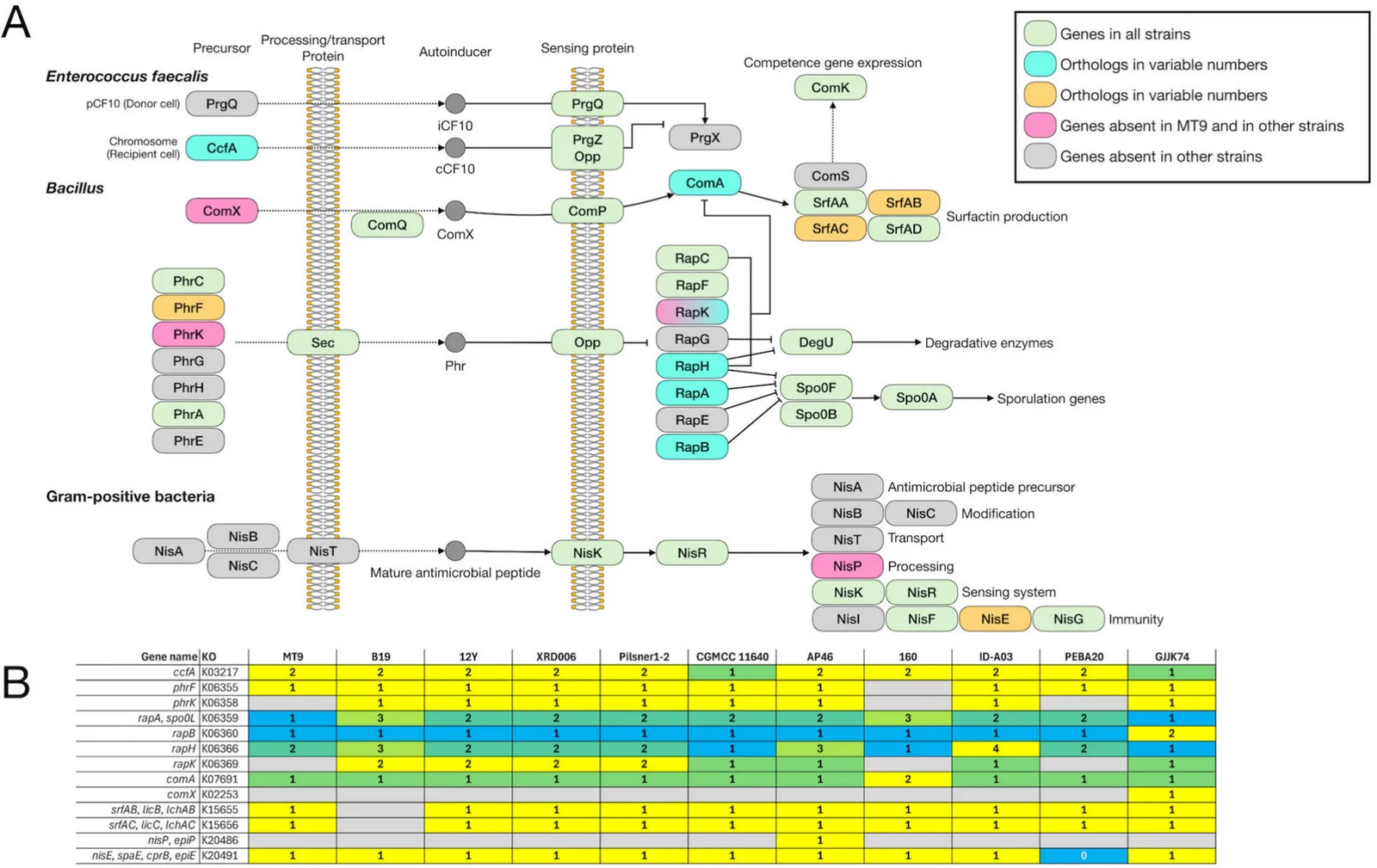
Differences between *B. velezensis* MT9 and ten *B. velezensis* reference strains in quorum-sensing genes as predicted by comparative KEGG analysis. Reconstruction of KEGG pathway (map02024) (**A**). Quorum-sensing circuits and corresponding genes in *B. velezensis* MT9 and ten *B. velezensis* reference strains (**B**)

As regards quorum sensing, the genes coding for PhrK and RapK, which are involved in the initiation of sporulation and competence development [96], and NisP, which is involved in processing of antimicrobial peptides [97], are absent in *B. velezensis* MT9 and in several *B. velezensis* strains (Fig. 8 and Fig. S15). The presence of gene clusters responsible for secondary metabolites biosynthesis in the *B. velezensis* MT9 genome was assessed using AntiSMASH 7.0 [89]. For comparison, this prediction was also performed on the genomes of ten other *B. velezensis* reference strains (B19, 12Y, XRD006, Pilsner 1–2, CGMCC, AP46, 160, A03, PEBA20, GJJK74).

**Fig. 9:**
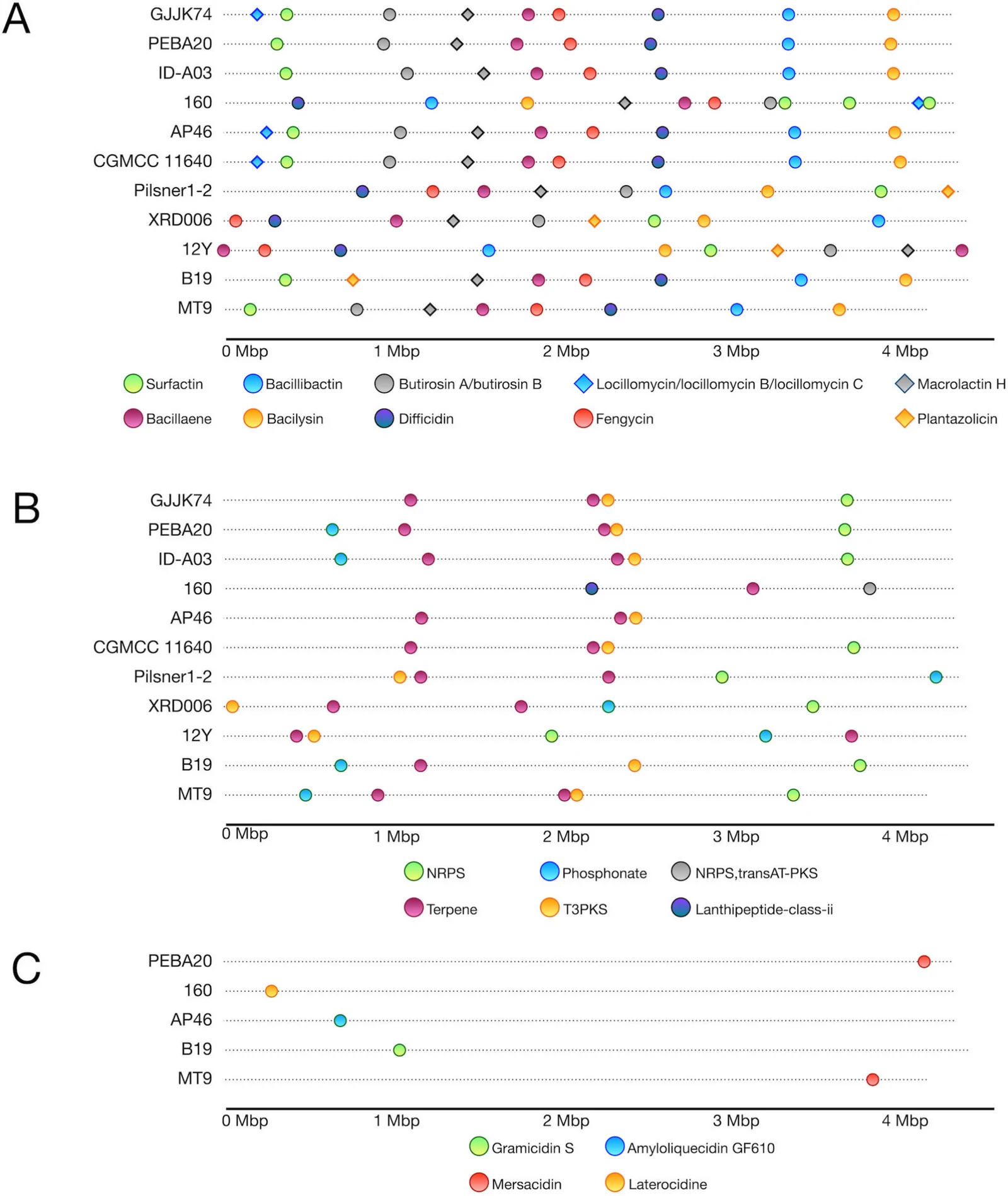
Gene clusters for secondary metabolites in the genome of *B. velezensis* MT9 as predicted by antiSMASH analysis. Gene clusters for secondary metabolites present in at least 4 of the 11 *B. velezensis* strains analyzed (**A**). Gene clusters for secondary metabolites classified at a general level by antiSMASH (**B**). Gene clusters for secondary metabolites present in up to 2 of the 11 *B. velezensis* strains analyzed (**C**)

The results are shown in Fig. 9. Ten clusters were present in at least 4 out of the 11 strains analyzed with AntiSMASH (Fig. 9A). These clusters were identified as bacillaene, bacillibactin, bacilysin, butyrosine A/butyrosine B, difficidin, fengycin, locillomycin/locillomycin B/locillomycin C, macrolactin H, plantazolicin, and surfactin. Clusters associated with bacillaene production were detected as a single copy in all strains analyzed, except *B. velezensis* 12Y, which has two copies of this cluster. Similarly, clusters associated with surfactin production were detected as a single copy in all strains, except *B. velezensis* 160, which has three copies. Biosynthetic clusters for bacillibactin, bacilysin, difficidin, fengycin, and macrolactin H were present as single copies in all strains.

The cluster responsible for butyrosine A/butyrosine B biosynthesis was found in all strains except *B. velezensis* B19. Two other clusters were found only in four strains: locillomycin/locillomycin B/locillomycin C (in strains CGMCC 11640, AP46, 160, GJJK74) and plantazolicin (in strains B19, 12Y, XRD006, Pilsner1 - 2). In some cases, AntiSMASH predicted unidentified clusters responsible for synthesizing compounds belonging to the NRPS, phosphonate, T3PKS, lanthipeptide, and terpene classes (Fig. 9B). Only in *B. velezensis* 160, an unknown cluster annotated as NRPS, transAT-PKS was predicted (Fig. 9B). Additionally, some annotated biosynthetic clusters were found only in several strains (Fig. 9C). In particular, *B. velezensis* MT9 showed a cluster annotated as mersacidin, which was present in only one other strain (PEBA20) (Fig. 9C).

Furthermore, *B. velezensis* MT9, namely bacillaene, bacillibactin, bacilysin, and difficidin have broad spectrum antimicrobial activity (Table 6) and are active against Proteobacteria (Pseudomonadota) whose relative abundance in the intestine of the Nile tilapia was reduced by *B. velezensis* MT9.

**Table 6:** Antimicrobial activities of predicted secondary metabolites of *B. velezensis* MT9

Secondary metabolite	Target microorganism	Reference
Bacillaene	*Campylobacter jejuni* *Streptomyces avermitilis* *Escherichia coli* SC10909 (hyperpermeable) *Escherichia coli* BAS847 (hyperpermeable) *Escherichia coli* K10 *Klebsiella pneumoniae* SC10440 *Klebsiella pneumoniae* SC9527 *Proteus vulgaris* SC9416 *Serratia marcescens* SC9783 *Bacillus thuringiensis* SC2928 *Staphylococcus aureus* SC2400 *Staphylococcus aureus* 209P *Staphylococcus epidermidis* SC9087 *Pseudomonas aeruginosa* SC8723 *Saccharomyces cerevisiae* SC1600 *Candida albicans* SC5314	[98] [99] [100]
Bacillibactin	*Pseudomonas syringae* pv. Tomato *Staphylococcus aureus* MRSA ATCC 33,592 *Enterococcus faecalis* VREfs ATCC 51,299 *Pseudomonas aeruginosa* ATCC 27,853 *Klebsiella pneumoniae* ATCC 13,883	[101] [102]
Bacilysin	*Staphylococcus. aureus* *Escherichia coli* *Candida albicans*	[103] [104]
Surfactin	*Staphylococcus. aureus* Adhesion of food pathogenic bacteria (*Listeria monocytogenes*, *Enterobacter sakazakii, Salmonella enterica* sv. Enteritidis) *Brachyspira hyodysenteriae* and *Clostridium perfringens*	[105] [106] [107]
Difficidin	*Staphylococcus aureus* Gm^R^ Meth^R^ *Staphylococcus aureus* *Escherichia coli* TEM 2 + *Escherichia coli* TEM 2 + DC2 *Escherichia coli* BC2 *Escherichia coli* *Salmonella enterica* sv. Typhimurium *Enterobacter cloacae* P99 + and P99- *Enterobacter aerogenes* *Klebsiella pneumoniae* K l + *Klebsiella pneumoniae* *Morganella morganii* Sm^R^ *Proteus vulgaris* *Proteus mirabilis* Gm^R^ *Pseudomonas aeruginosa* R PL 1 1 + 3350 8. 0 *Pseudomonas aeruginosa* *Serratia marcescens*	[108]
Fengycin	*Magnaporthe grisea* Zygomycota Ascomycota Dothideales and related Deuteromycota Basidiomycota and related Deuteromycota	[109] [110]
Macrolactin H	*Staphylococcus aureus* *Bacillus subtilis*	[111]
Plantazolicin	*Bacillus anthracis*, but not other members of the *Bacillus cereus* group (ultra-narrow)	[112]
Mersacidin	*Staphylococcus aureus* (MRSA) *Micrococcus luteus* *Staphylococcus* spp. *Streptococcus* spp. *Clostridium* spp. Other Gram-positive bacteria	[113] [114] [115]

**Fig. 10:**
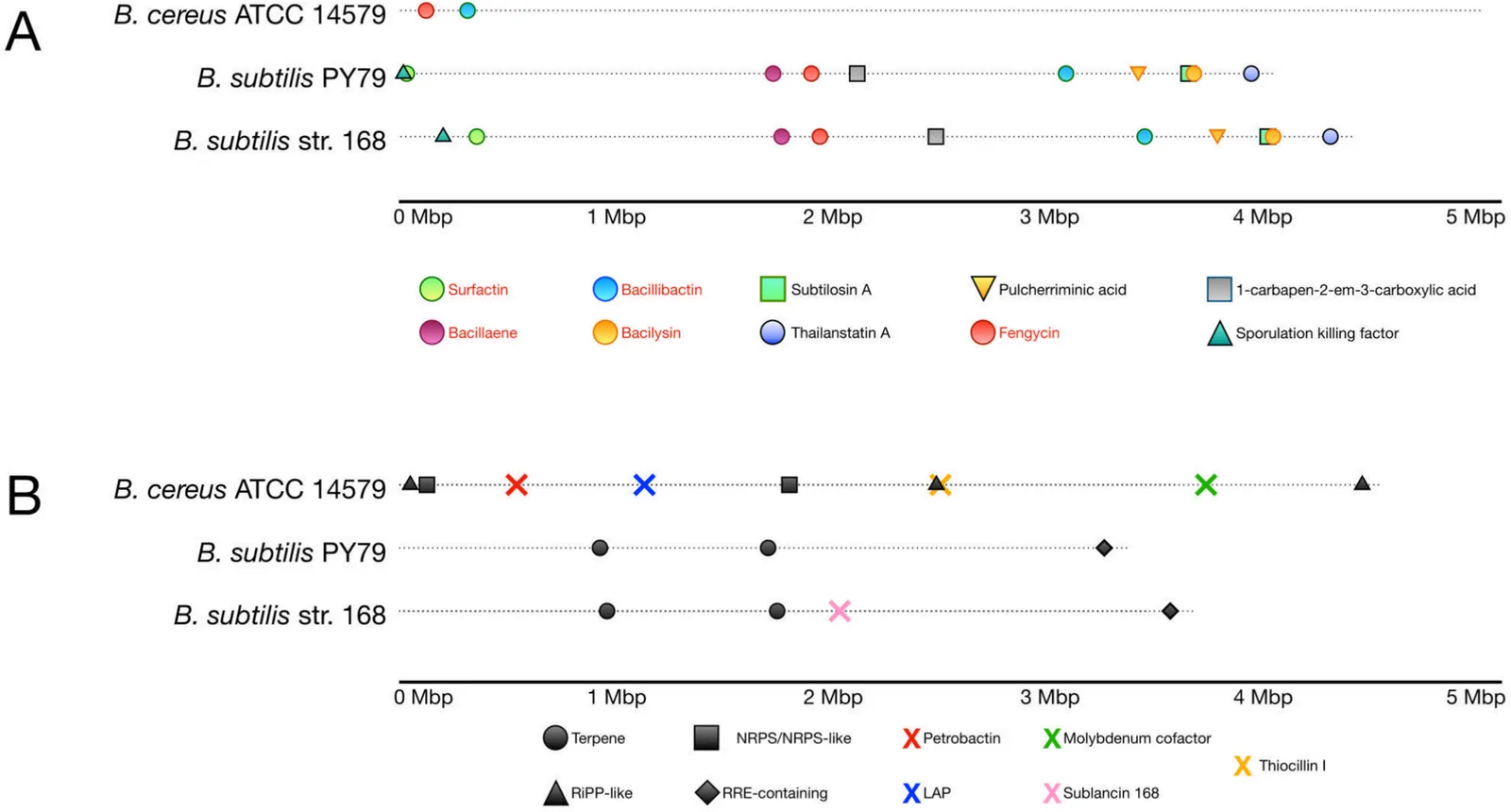
Gene clusters for secondary metabolites in the genome of *B. subtilis* and *B. cereus* reference strains as predicted by antiSMASH analysis. Gene clusters for secondary metabolites present in at least two of the three *Bacillus* species analyzed. Red text highlights clusters common between *B. subtilis* and *B. cereus* reference strains and the 11 *B. velezensis* strains analyzed (**A**). Gene clusters for secondary metabolites classified at a general level by antiSMASH and gene clusters for secondary metabolites present in up to 1 *B. subtilis* and *B. cereus* reference strains (**B**)

Finally, surfactin inhibits the adhesion of these bacteria and antiSMASH predicted that bacillaene, bacillibactin, bacilysin, and surfactin are produced also by *B. subtilis* and *B. cereus* (Fig. 10 A–B). *Bacillus velezensis*, *B. cereus*, and *B. subtilis* also share the ability to produce fengycin, an antifungal compound (Fig. 10 A–B).

## Discussion

With the development of aquaculture technologies, it has been commonly observed that the dysfunction of the intestine leads to inefficient digestion and absorption of nutrients and even becomes one important cause of diseases [116]. In this scenario, in the present study, the capability of the strain *Bacillus velezensis* MT9, as potential probiotic, to modulate the intestinal microbiota of Nile tilapia *(Oreochromis niloticus)* fed with the *B. velezensis*-supplemented feed in an experimental aquaculture plant was investigated. From the results obtained, the following interesting issues can be inferred:

- The concentration of culturable bacteria at 37 °C was lower in the water in which the fish were fed with *B. velezensis*-enriched feed were raised compared to the control water. This type of analysis has the characteristic of being able to evaluate the microbiological quality of the water, detecting the presence of microorganisms of fecal origin, which can alter the organoleptic characteristics of the water or give rise to opportunistic infections. A high value of the bacterial colony counts at 37 °C can represent an early sign of pollution. On the basis of our results, it is possible to conclude that the changes in the fish intestinal microbiota due to the action of *B. velezensis* are also reflected in the improvement of the breeding water quality with a decrease in the bacteria culturable at 37 °C of an order of magnitude. This is also confirmed by the results regarding the microbiological concentration of culturable bacteria at 37 °C in the intestine of treated and control fish, which showed a higher concentration of these bacteria in the intestine of control farmed fish than in the intestine of fish fed with *B. velezensis*-based feed. This trend was also evidenced in the case of the monitored microbial pollution indicators. As regards the water samples, indeed, total coliforms reached the mean value of 2419.6 ± 62.0 MPN/100 mL in the control water and a value one-order of magnitude lower in the water of the tanks in which the organisms were fed with *B. velezensis* MT9-enriched feed. In the case of the fish intestine, total coliforms, fecal coliforms, and *E. coli* mean densities resulted higher in the intestine of fish fed with conventional feed when compared to the values recorded in fish nourished with *B. velezensis* MT9-enriched feed. Such results are in accordance with those obtained from the analyses obtained from the 16S rRNA gene metabarcoding approach showing that the treatment with *B. velezensis* MT9 resulted in a decrease in the relative abundance of bacteria belonging *Escherichia/Shigella* genera, which include members pathogenic to fish thus leading to suggest a reduction of potential fish or human diseases [117, 118]. Furthermore, the beneficial effect of *B. velezensis* MT9 on reared fish was also highlighted in the modulation of the water microbiota, with a reduction in the relative abundance of the phyla Planctomycetes, Actinobacteria, Chloroflexi, Nitrospirae, and Fusobacteria as well as an increase of the several phyla including Bacteroidetes and bacteria belonging to unclassified candidate phyla. Such differences were reflected in the differences found at the level of class, family, and genus. Modulation of water microbiota could be caused by the production of secondary metabolites by *B. velezensis* or by its ability to degrade organic matter. However, further studies are needed to confirm these hypotheses.
  Treatment with *B. velezensis* MT9 also resulted in a decrease in the relative abundance of bacteria belonging to the *Vibrio* and *Aeromonas* in the intestine microbiota. This finding is of particular relevance because the *Aeromonas* genus consists of Gram-negative microbes reported as opportunistic fish pathogens and often isolated from injured fish [116]. Several strategies have been proposed to prevent the colonization of fish by these opportunistic pathogens including the use of probiotics [119] and phages [120]. Noteworthy, dietary supplementation of *Bacillus* *cereus* and *Bacillus subtilis* was shown to reshape the intestinal bacterial community of Pengze crucian carp with a notable reduction in *Aeromonas* and an increase in *Romboutsia* [121]. This finding suggests that *B. velezensis *MT9 may share with other members of the *Bacillus *genus the same positive/negative interactions with specific genera of the intestinal microbiota. Regarding culturable *Vibrio*, several studies have shown that bacteria belonging to this genus are of particular concern as they are often associated with vibriosis, diseases that can affect farmed organisms with important economic consequences. Vibriosis is one of the major constraints on aquaculture production and one of the main bacterial diseases observed in farmed organisms worldwide; therefore, the absence of culturable vibrios in the water tanks gives us comfort regarding the healthiness of the farming conditions and the welfare of the tilapia as these microorganisms were also absent in the intestinal microbiota of all farmed fish.
- In the BV-MT9 group compared to the CTL group, the greater relative abundance of the genus *Romboutsia* at T3 was noteworthy. This finding may reflect additional beneficial effects of dietary supplementation of Nile tilapia with *B. velezensis* MT9 because some members of the genus *Romboutsia* have documented probiotic activity [122–124]. *Romboutsia* are obligate anaerobes that ferment carbohydrates and metabolize aromatic amino acids to produce phenolic and indolic compounds that play important roles in regulating biological processes such as intestinal epithelial cell homeostasis, immune cell responses, and neuronal excitability [123, 124]. Furthermore, they produce short chain fatty acids, which play a crucial role in maintenance of intestinal epithelial barrier function [125].
- The benefical effect of *B. velezensis* MT9 on Nile tilapia was also highlighted by the greater relative abundance of the genus *Cetobacterium* in the BV-MT9 group compared to the CTL group. *Cetobacterium*, which has been identified as a major component of the gut microbiota of freshwater fish including *Oreochromis niloticus* [126], *Cyprinus carpio* [127], *Arapaima gigas* [128], *Lepomis macrochirus*, *Micropterus salmoides*, and *Ictalurus punctatus* [129], is indeed characterized by high vitamin B12-producing ability [126]. It was reported that Japanese eel, ayu, carp, tilapia, goldfish, and catfish—specifically, carp and tilapia with high levels of *Cetobacterium* (previously classified as *Bacteroides* type A [126]—did not require dietary vitamin B12 and showed high levels of vitamin B12 in their intestinal content [130].
- From the analysis of the intestinal bacterial community, an increase in the relative abundance of the genera belonging to the Actinomycetota phylum at T2, especially in the BV-MT9 group, was evidenced. The increase in relative abundance observed is attributable mainly to the genera *Nocardia, Mycobacterium,* and other less abundant genera (*Streptomyces*, *Rhodococcus*, and *Propionibacterium*)*.* The increase in Actinomycetota has different implications, positive or negative, depending on the genera and species. On one hand, the increase in the relative amount of *Nocardia* in the Nile tilapia with dietary supplementation with *B. velezensis* MT9 is potentially negative for fish, because several members of this genus are responsible for nocardiosis, one of the most common fish diseases whose symptoms may include granuloma formation, epidermis abscesses, tubercles in the gills, kidneys, and spleen [131–133]. Moreover, infection by *Nocardia* induces dysbiosis [134], and biocontrol strategy with *Bacillus subtilis* has been proposed against pathogenic *Nocardia seriolae* [135]. The increase in the relative amount of *Mycobacterium* in the Nile tilapia with dietary supplementation with *B. velezensis* MT9 may also be potentially negative, because several mycobacteria may be responsible for mycobacteriosis in the Nile tilapia [136].

On the other hand, the increase in the relative amounts of other genera belonging to the Actinomycetota phylum, including *Streptomyces* and *Rhodococcus*, is potentially positive for fish. *Rhodococcus* have a documented probiotic activity. Furthermore, only few species are pathogenic [137]. Several strains of *Rhodococcus* improve the immune response in fish, enhance their resistance to pathogens such as *Vibrio anguillarum* [138], modulate the water microbiota limiting the growth of pathogens such as *Flavobacterium psycrophilum* [139], and degrade mycotoxins [140]. *Streptomyces* are prolific producers of secondary metabolites that can antagonize the growth of pathogens and biofilm formation by producing secondary metabolites including antibiotics, anticancer agents, antiparasitic agents, antifungal agents, and enzymes (protease and amylase) [141]. Several strains of this genus have antagonistic and antimicrobial activity against aquaculture pathogens by producing bacteriocins, siderophores, hydrogen peroxide, and organic acids, and their use as probiotic agents in aquaculture is very promising [141, 142]. As regards genera exerting probiotic activity, gut bacterial community of Nile tilapia with dietary supplementation with *B. velezensis* MT9 showed an important increase in the relative amount of *Paenibacillus*. Several strains belonging to this genus produce bacteriocins and hydrolytic enzymes, which play a role in pathogen defense [143–146], and there is evidence that bacteriocin-producing *Paenibacillus* strains can improve feed efficiency, enhance growth, and promote innate immunity against pathogen infection in Nile tilapia [147].

- In the BV-MT9 group compared to the CTL group, we observed an increase in the relative abundance of *Parachlamydia* at T3. We do not know whether the increase in the relative amount of these bacteria is due to infection by *Acanthamoeba* or other free-living amoebae, which may infect the intestine of the Nile tilapia even with limited inflammatory response [148]. Future investigations are needed to clarify this aspect.

Overall, these results support the hypothesis of employing *B. velezensis* MT9 as a probiotic for Nile tilapia. Indeed, although the addition of *B. velenzensis* MT9 did not affect the survival rate or growth performance of fish in our limited set of experiments, this bacterium meets a number of criteria for the definition of a probiotic. The lack of a growth-promoting effect is not a determining factor, as probiotics are defined by FAO/WHO as “live microorganisms which, when consumed in adequate amounts as part of food, confer a health benefit on the host” [149]. This definition implies that any observed growth effects are mostly secondary to improvements in fish health, increasing disease resistance, enhancing immune response, and improving the microbiological quality of fish and water for better survival. Furthermore, it may be noted that the European Food Safety Authority (EFSA) has included *B. velezensis* in its list of substances subject to Qualified Presumption of Safety (QPS) [44]. In the current study, we provide a first evidence that *B. velezensis* MT9 has a strong ability to modulate the intestinal microbiota of Nile tilapia by reducing the relative abundance of opportunistic fish pathogens belonging to the Gammaproteobacteria class. This finding indicates that *B. velezensis* MT9 has the potential of improving the microbiological quality of fish, enhancing fish health by preventing infections, and promoting sustainable aquaculture practices. However, further studies are required to confirm the finding of this study to complete the characterization of *B. velezensis* MT9 and to establish its status as a probiotic rather than a “potential probiotic” for Nile tilapia. Further studies are needed also examining a greater number of samples since another limitation of the present study is that only one test (using aggregated samples) was conducted for each sampling time, in order to analyze the evolution of the system. Therefore, further tests will serve to consolidate the results obtained. Moreover, to further analyze the potential probiotic activity of *B. velezensis* MT9, we used WGS tools. Indeed, WGS is considered a mandatory method for the characterization of probiotics [150]. The complete genome sequencing of *B. velezensis* MT9 revealed that this bacterium lacks proteins involved in virulence or pathogenicity and thus poses no risk to animal or human health, as previously reported in literature for *B. velezensis* [32, 151]. Similarly, the bacterium exhibited few antibiotic resistance genes, no plasmids, and only a few genomic regions containing phage proteins. These findings underscore that the bacterium is genetically stable and does not contribute to the dissemination of antibiotic resistance genes.

Comparative genomic analysis of *B. velezensis* MT9 and ten other strains highlighted several differences, such as the absence of genes involved in amino acids transport, β-glucoside transport, and DNA recombination and repair. In addition, the comparative genomic analysis revealed the absence of the *rpmH* gene, which encodes the L34 ribosomal protein, in *B. velezensis* MT9 [79, 80]. This protein is involved in the normal assembly of the 50S ribosomal subunit and in efficient formation of the 70S ribosome. Lack of this protein in *B. subtilis* results in an abnormal accumulation of both 30S and 50S subunits and slow growth [152]. These defects can be complemented partially by Mg^2+^ which is known to stabilize the secondary structure of rRNA, the binding of the ribosomal proteins to the rRNA, and the interaction between the 30S and 50S subunits [152, 153]. This genetic feature (absence of the *rpmH* gene) and its possible compensation by Mg^2+^ could reflect specific characteristics of the ecological niche from which *B. velezensis* MT9 was isolated. Mg is an important element for tilapia aquaculture [154]. Therefore, the effect of Mg on this *B. velezensis* should be considered for future development of industrial feeding plans.

The KEGG analysis also highlighted other differences, such as the absence of the *tagE*, which encodes an enzyme involved in the biosynthesis of teichoic acids in some *B. velezensis* strains. Mutations in *tagE* are associated with resistance to several bacteriophages that recognize glucose residues on teichoic acid as a receptor [155–158]. Resistance to bacteriophages is a recognized positive trait for probiotics, as it ensures the survival and efficacy of the probiotic during colonization of the gut [159, 160] and because they favor the genetic stability. Further studies are needed to confirm the resistance of *B. velezensis* MT9 to bacteriophages.

Additionally, the analysis revealed that several genes associated with competence are absent in *B. velezensis* MT9, including the ComX protein, which is involved in competence in *B. subtilis* [94], as well as the PhrK and RapK proteins [96]. These genetic traits could constitute additional favorable elements for the use of *B. velezensis* MT9 as a potential probiotic, since they could ensure greater genetic stability.

AntiSMASH 7.0 [89] was used to predict gene clusters responsible for the biosynthesis of secondary metabolites in the genome of *B. velezensis* MT9 and ten other strains. Several clusters, including genes for the synthesis of bacillaene, bacillibactin, bacilysin, butyrosine A/butyrrhosine B, difficidin, fengycin, locillomycin/locillomycin B/locillomycin C, macrolactin H, plantazolicin, and surfactin, were present in the genome of *B. velezensis* MT9, as well as in many other strains analyzed. These clusters, therefore, appear to be highly common. In contrast, *B. velezensis* MT9 possessed a cluster annotated as mersacidin, which is not widespread among these bacteria, suggesting that this strain could be a source of novel molecules of interest for industrial and pharmaceutical applications. Mersacidin has garnered significant attention for its unique mechanism of action and potential therapeutic applications, particularly against Gram-positive pathogens, including methicillin-resistant *S. aureus* (MRSA) [161–163].

It may be also interesting to note that several predicted secondary metabolites of *B. velezensis* MT9, namely bacillaene, bacillibactin, bacilysin, and difficidin have broad spectrum antimicrobial activity, as stated below, and are active against Proteobacteria (Pseudomonadota) whose relative abundance in the intestine of the Nile tilapia was reduced by *B. velezensis* MT9. Furthermore, surfactin is capable to inhibit the adhesion of these bacteria. Noteworthy, *B. subtilis* and *B. cereus* produce antibacterial compounds such as bacillaene, bacillibactin, bacilysin, and surfactin which, similarly to *B. velezensis* MT9, were shown to inhibit the growth of Proteobacteria (Pseudomonadota) in the gut of farmed Pengze crucian carp [121]. In addition*, B. velezensis, B. cereus*, and *B. subtilis* are also capable to produce antifungal compounds. The production of bacillaene, bacillibactin, bacilysin, and surfactin by these species of *Bacillus* could explain their ability to antagonize the growth of Proteobacteria (Pseudomonadota) in the intestinal lumen of fish.

## Conclusions

This study demonstrates that the use of probiotics can be particularly successful in the fields of animal husbandry and aquaculture. In fact, if on the one hand, probiotics have very interesting prospects in the field of human medicine in the prevention and control of many diseases, on the other, their efficacy is difficult to standardize due to a plethora of confounding factors such as the host’s genetics, geography, environment, diet, lifestyle, and, above all, nutrition, which have, in themselves, a strong impact on the modulation of the intestinal microbiota. In aquaculture, in particular in closed systems (integrated recycling systems), most of these factors are under control, so the actual beneficial effect of probiotics is well standardizable. This explains why the main results that were obtained with the use of *B. velezensis* MT9 in the Nile tilapia (namely, the increase in the relative abundance of bacteria of the genus *Romboutsia*, which has well-documented probiotic activity, and the decrease in the relative abundance of Gammaproteobacteria of the genera *Aeromonas* and *Vibrio*, which include opportunistic pathogens for fish) are quite consistent with the results of another study using other bacteria of the same genus Bacillus (*B. cereus* and *B. subtilis*) in the Pengze crucian carp [121]. This finding suggests that *B. velezensis* MT9 may share with *B. cereus* and *B. subtilis* (and possibly other members of the *Bacillus* genus) the same positive/negative interactions with specific genera of the intestinal microbiota. In particular, comparative genomic analysis suggests that *B. velezensis*, *B. cereus*, and *B. subtilis* share the ability to produce an armamentarium of secondary metabolites, which may be responsible for antagonistic interactions with Gammaproteobacteria. Although these data are preliminary, the present study contributes to further investigate the complexity of tilapia intestinal microbiota considering that improving intestinal health would greatly promote the healthy and sustainable development of aquaculture. Understanding the prevalence, ecology, concentration, and dynamics of pathogenic and spoilage microorganisms present along the entire fish production chain would also contribute to the development and application of novel intervention strategies.

However, further studies need to be conducted before using *B. velezensis* MT9 as a probiotic on fish. According to the regulation (EC) No. 429/2008 [164], live microorganisms administered as feed additives must comply with specific requirements. Some requirements were met in this study, such as the absence of toxins, virulence factors, and antibiotic resistance genes, and the ability to modulate the intestinal microbiota of Nile Tilapia by reducing the relative abundance of potential pathogenic microorganisms to fish or humans. However, this last evidence is preliminary and requires further confirmation. The regulation calls for an in vivo tolerance test, in which the potential probiotic must be administered to the target species for a fixed period of 90 days for fish such as salmon and trout. The present study adheres to that timeline. However, the regulation also requires that the potential probiotic be administered to an experimental group at ten times the maximum recommended dose. Further studies are therefore necessary to clarify the tolerability of *B. velezensis* MT9. Other aspects defined by this regulation remain to be fully addressed, including environmental toxicity and environmental safety. Additionally, the stability of feed supplemented with *B. velezensis* needs to be evaluated over longer periods, and the number of viable bacteria proliferating in the fish intestine must be fully demonstrated. For these reasons, this study serves as a preliminary test, providing partial feedback on the requirements outlined in Regulation (EC) No. 429/2008. Further research will also go into evaluating the effect of *B. velezensis* MT9 administration on stress response and exposure to Nile tilapia pathogens, as well as on fish product quality.

## Supplementary Information

Below is the link to the electronic supplementary material.

ESM 1 (PNG 1.35 MB)

Supplementary file1 Fig. S1. Analysis of the gut microbial community of Nile tilapia at family level in the control (CTL) and treatment (BV MT9) (TIFF 958 KB)

ESM 2 (PNG 965 KB)

Supplementary file2 Fig. S2. Analysis of the water from the Nile tilapia plant at phylum (A) and class levels (B) in the control (CTL) and treatment (BV MT9) (TIFF 774 KB)

ESM 3 (PNG 2.07 MB)

Supplementary file3 Fig. S3. Analysis of the water from the Nile tilapia plant at family level in the control (CTL) and treatment (BV MT9) (TIFF 1425 KB)

ESM 4 (PNG 1.61 MB)

Supplementary file4 Fig. S4. Analysis of the water from the Nile tilapia plant at genus level in the control (CTL) and treatment (BV MT9) (TIFF 1275 KB)

ESM 5 (PNG 528 KB)

Supplementary file5 Fig. S5. Metabolic reconstruction of Nucleotide metabolism (map01232) using KEGG Mapper (TIFF 816 KB)

ESM 6 (PNG 784 KB)

Supplementary file6 Fig. S6. Metabolic reconstruction of Starch and sucrose metabolism (map00500) using KEGG Mapper (TIFF 1159 KB)

ESM 7 (PNG 741 KB)

Supplementary file7 Fig. S7. Metabolic reconstruction of Amino sugar and nucleotide sugar metabolism (map00520) using KEGG Mapper. Genes involved in sugar metabolism (A). Reconstruction of KEGG pathway (B) (TIFF 943 KB)

ESM 8 (PNG 900 KB)

Supplementary file8 Fig. S8. Metabolic reconstruction of ABC transporters (map02010) using KEGG Mapper. Genes encoding ABC transporters (A). Reconstruction of KEGG pathway (B) (TIFF 1045 KB)

ESM 9 (PNG 487 KB)

Supplementary file9 Fig. S9. Metabolic reconstruction of Phosphotransferase system (map02060) using KEGG Mapper. Genes encoding proteins involved in phosphotransferase systems (A). Reconstruction of KEGG pathway (B) (TIFF 624 KB)

ESM 10 (PNG 328 KB)

Supplementary file10 Fig. S10. Metabolic reconstruction of One carbon pool by folate (map00670) using KEGG Mapper. Genes involved in one carbon pool by folate metabolism (A). Reconstruction of KEGG pathway (B) (TIFF 460 KB)

ESM 11 (PNG 523 KB)

Supplementary file11 Fig. S11. Metabolic reconstruction of Pyrimidine metabolism (map00240) using KEGG Mapper. Genes involved in pyrimidine metabolism (A). Reconstruction of KEGG pathway (B) (TIFF 657 KB)

ESM 12 (PNG 463 KB)

Supplementary file12 Fig. S12. Metabolic reconstruction of Homologous recombination (map03440) using KEGG Mapper. Genes encoding proteins involved in homologous recombination systems (A). Reconstruction of KEGG pathway (B) (TIFF 619 KB)

ESM 13 (PNG 755 KB)

Supplementary file13 Fig. S13. Mapping of Ribosomal proteins (map03010) using KEGG Mapper (TIFF 862 KB)

ESM 14 (PNG 879 KB)

Supplementary file14 Fig. S14. Metabolic reconstruction of Two-component system (map02020) using KEGG Mapper. Genes encoding proteins involved in two-component systems (A). Reconstruction of KEGG pathway (B) (TIFF 1213 KB)

ESM 15 (PNG 747 KB)

Supplementary file15 Fig. S15. Metabolic reconstruction of Quorum sensing (map02024) using KEGG Mapper. Genes involved in quorum sensing (A). Reconstruction of KEGG pathway (B) (TIFF 1245 KB)

Supplementary file16 Table S1. Number of reads and quality control (DOCX 14 KB)

Supplementary file17 Table S2. BUSCO statistics (DOCX 14 KB)

Supplementary file18 Table S3. Results of the taxonomic identification using the NCBI Assembly and ANI report (XLSX 11 KB)

Supplementary file19 Table S4. Results of the taxonomic identification using the Genome Taxonomy database (XLSX 10 KB)

Supplementary file20 File S1. 16S gene metabarcoding analysis of samples collected in T0 time-point (before the start of the experiment) in the control (CTL) and treatment (BV MT9). Complete set of data (XLSX 50 KB)

Supplementary file21 File S2. 16S gene metabarcoding analysis of samples collected in T1, T2 and T3 time-points in the control (CTL) and treatment (BV MT9). Complete set of data (XLSX 293 KB)

Supplementary file22 File S3. KEGG mapper analysis (XLSX 26 KB)

## Data Availability

No datasets were generated or analysed during the current study.

## References

[1] UN General Assembly (2015) Resolution adopted by the general assembly on September 25, 2015. Transforming our world: the 2030 agenda for sustainable development.

[2] United Nations (2019) World population prospects

[3] Cottrell RS, Blanchard JL, Halpern BS et al (2020) Global adoption of novel aquaculture feeds could substantially reduce forage fish demand by 2030. Nat Food 1:301–308. 10.1038/s43016-020-0078-x

[4] Searchinger T, Waite R, Hanson C, et al. (2018) Creating a sustainable food future: a menu of solutions to feed nearly 10 billion people by 2050‐synthesis report

[5] Willett W, Rockström J, Loken B et al (2019) Food in the anthropocene: the EAT–Lancet Commission on healthy diets from sustainable food systems. The Lancet 393:447–492. 10.1016/S0140-6736(18)31788-4 10.1016/S0140-6736(18)31788-4 30660336

[6] Naylor RL, Kishore A, Sumaila UR et al (2021) Blue food demand across geographic and temporal scales. Nat Commun 12:5413. 10.1038/s41467-021-25516-4 34526495 10.1038/s41467-021-25516-4 PMC8443621

[7] Sarkar B, Mahanty A, Gupta SK et al (2022) Nanotechnology: a next-generation tool for sustainable aquaculture. Aquaculture 546:737330. 10.1016/j.aquaculture.2021.737330

[8] Cottrell RS, Ferraro DM, Blasco GD et al (2021) The search for blue transitions in aquaculture-dominant countries. Fish Fish 22:1006–1023. 10.1111/faf.12566

[9] Longo SB, Clark B, York R, Jorgenson AK (2019) Aquaculture and the displacement of fisheries captures. Conserv Biol 33:832–841. 10.1111/cobi.13295 30719749 10.1111/cobi.13295

[10] Yuan J, Xiang J, Liu D et al (2019) Rapid growth in greenhouse gas emissions from the adoption of industrial-scale aquaculture. Nat Clim Chang 9:318–322. 10.1038/s41558-019-0425-9

[11] Tacon AGJ, Metian M, McNevin AA (2022) Future feeds: suggested guidelines for sustainable development. Reviews in Fisheries Science & Aquaculture 30:271–279. 10.1080/23308249.2021.1898539

[12] Costello C, Cao L, Gelcich S et al (2020) The future of food from the sea. Nature 588:95–100. 10.1038/s41586-020-2616-y 32814903 10.1038/s41586-020-2616-y

[13] Hicks CC, Cohen PJ, Graham NAJ et al (2019) Harnessing global fisheries to tackle micronutrient deficiencies. Nature 574:95–98. 10.1038/s41586-019-1592-6 31554969 10.1038/s41586-019-1592-6

[14] FAO (2022) The state of world fisheries and aquaculture 2022. Towards blue transformation

[15] El-Sayed AM, Fitzsimmons K (2023) From Africa to the world—the journey of Nile tilapia. Rev Aquac 15:6–21. 10.1111/raq.12738

[16] El-Sayed A-FM (2002) Effects of stocking density and feeding levels on growth and feed efficiency of Nile tilapia (*Oreochromis niloticus* L.) fry: stocking density and feeding levels of Nile tilapia fry. Aquac Res 33:621–626. 10.1046/j.1365-2109.2002.00700.x

[17] Hai NV (2015) The use of probiotics in aquaculture. J Appl Microbiol 119:917–935. 10.1111/jam.12886 26119489 10.1111/jam.12886

[18] López Nadal A, Ikeda-Ohtsubo W, Sipkema D et al (2020) Feed, microbiota, and gut immunity: using the Zebrafish model to understand fish health. Front Immunol 11:114. 10.3389/fimmu.2020.00114 32117265 10.3389/fimmu.2020.00114 PMC7014991

[19] Hoseinifar SH, Sun Y-Z, Wang A, Zhou Z (2018) Probiotics as means of diseases control in aquaculture, a review of current knowledge and future perspectives. Front Microbiol 9:2429. 10.3389/fmicb.2018.02429 30369918 10.3389/fmicb.2018.02429 PMC6194580

[20] Ringø E, Van Doan H, Lee SH et al (2020) Probiotics, lactic acid bacteria and bacilli: interesting supplementation for aquaculture. J Appl Microbiol 129:116–136. 10.1111/jam.14628 32141152 10.1111/jam.14628

[21] Sadat Hoseini Madani N, Adorian TJ, Ghafari Farsani H, Hoseinifar SH (2018) The effects of dietary probiotic Bacilli (*Bacillus subtilis* and *Bacillus licheniformis*) on growth performance, feed efficiency, body composition and immune parameters of whiteleg shrimp (*Litopenaeus vannamei*) postlarvae. Aquac Res 49:1926–1933. 10.1111/are.13648

[22] Wang A, Ran C, Wang Y et al (2019) Use of probiotics in aquaculture of China — a review of the past decade. Fish Shellfish Immunol 86:734–755. 10.1016/j.fsi.2018.12.026 30553887 10.1016/j.fsi.2018.12.026

[23] El-Saadony MT, Alagawany M, Patra AK et al (2021) The functionality of probiotics in aquaculture: an overview. Fish Shellfish Immunol 117:36–52. 10.1016/j.fsi.2021.07.007 34274422 10.1016/j.fsi.2021.07.007

[24] Feng P, Ye Z, Kakade A et al (2018) A review on gut remediation of selected environmental contaminants: possible roles of probiotics and gut microbiota. Nutrients 11:22. 10.3390/nu11010022 30577661 10.3390/nu11010022 PMC6357009

[25] Chen L, Lam JCW, Tang L et al (2020) Probiotic modulation of lipid metabolism disorders caused by perfluorobutanesulfonate pollution in zebrafish. Environ Sci Technol 54:7494–7503. 10.1021/acs.est.0c02345 32459962 10.1021/acs.est.0c02345

[26] Sun B, Liu M, Tang L et al (2021) Probiotic supplementation mitigates the developmental toxicity of perfluorobutanesulfonate in zebrafish larvae. Sci Total Environ 799:149458. 10.1016/j.scitotenv.2021.149458 34365260 10.1016/j.scitotenv.2021.149458

[27] Sun B, Liu M, Tang L et al (2021) Probiotics inhibit the stunted growth defect of perfluorobutanesulfonate via stress and thyroid axes in zebrafish larvae. Environ Pollut 290:118013. 10.1016/j.envpol.2021.118013 34428700 10.1016/j.envpol.2021.118013

[28] Islam SMM, Rohani MF, Shahjahan M (2021) Probiotic yeast enhances growth performance of Nile tilapia (*Oreochromis niloticus*) through morphological modifications of intestine. Aquaculture Reports 21:100800. 10.1016/j.aqrep.2021.100800

[29] Jahan N, Islam SMM, Rohani MF et al (2021) Probiotic yeast enhances growth performance of rohu (*Labeo rohita*) through upgrading hematology, and intestinal microbiota and morphology. Aquaculture 545:737243. 10.1016/j.aquaculture.2021.737243

[30] Kitada Y, Muramatsu K, Toju H, et al. (2018) Bioactive polyamine production by a novel hybrid system comprising multiple indigenous gut bacterial strategies. Sci Adv 4:eaat0062. 10.1126/sciadv.aat0062 10.1126/sciadv.aat0062 PMC6021145 29963630

[31] Yang G, Cao H, Jiang W et al (2019) Dietary supplementation of *Bacillus cereus* as probiotics in Pengze crucian carp (*Carassius auratus* var. Pengze): effects on growth performance, fillet quality, serum biochemical parameters and intestinal histology. Aquac Res 50:2207–2217. 10.1111/are.14102

[32] Calcagnile M, Tredici SM, Alifano P (2024) A comprehensive review on probiotics and their use in aquaculture: biological control, efficacy, and safety through the genomics and wet methods. Heliyon 10(24):e40892. 10.1016/j.heliyon.2024.e40892 39735631 10.1016/j.heliyon.2024.e40892 PMC11681891

[33] Zhou P, Chen W, Zhu Z, Zhou K, Luo S, Hu S, Xia L, Ding X (2022) Comparative study of *Bacillus amyloliquefaciens* X030 on the intestinal flora and antibacterial activity against *Aeromonas* of Grass Carp. Front Cell Infect Microbiol 12:815436. 10.3389/fcimb.2022.815436 35145928 10.3389/fcimb.2022.815436 PMC8821659

[34] Sam-On MFS, Mustafa S, Mohd Hashim A, Yusof MT, Zulkifly S, Malek AZA, Roslan MAH, Mohd Asrore MS (2023) Mining the genome of Bacillus velezensis FS26 for probiotic markers and secondary metabolites with antimicrobial properties against aquaculture pathogens. Microb Pathog 181:106161. 10.1016/j.micpath.2023.106161 37207784 10.1016/j.micpath.2023.106161

[35] Nasreen S, Ali S, Andleeb S, Summer M, Hussain T, Imdad K, Ara C, Tahir HM (2024) Mechanisms of medicinal, pharmaceutical, and immunomodulatory action of probiotics bacteria and their secondary metabolites against disease management: an overview. Folia Microbiol (Praha) 69(3):549–565. 10.1007/s12223-024-01155-2. (Epub 2024 Mar 27 PMID: 38532057) 38532057 10.1007/s12223-024-01155-2

[36] Calcagnile M, Tredici MS, Pennetta A et al (2022) *Bacillus velezensis* MT9 and Pseudomonas chlororaphis MT5 as biocontrol agents against citrus sooty mold and associated insect pests. Biol Control 176:105091. 10.1016/j.biocontrol.2022.105091

[37] Chen B, Zhou Y, Duan L, Gong X, Liu X, Pan K, Zeng D, Ni X, Zeng Y (2023) Complete genome analysis of Bacillus velezensis TS5 and its potential as a probiotic strain in mice. Front Microbiol 14:1322910. 10.3389/fmicb.2023.1322910 38125573 10.3389/fmicb.2023.1322910 PMC10731255

[38] Gao X, Chen A, Yifan Zhou Y, Qian Q, Qin L, Tang X, Jiang Q, Zhang X (2025) Genomic characterization and probiotic potency of Bacillus velezensis CPA1-1 reveals its potential for aquaculture applications. Aquaculture 596:741852. 10.1016/j.aquaculture.2024.741852

[39] Ghosh, T. (2024). Recent advances in the probiotic application of the Bacillus as a potential candidate in the sustainable development of aquaculture. *Aquaculture*, 741432.

[40] Kuebutornye FKA, Abarike ED, Lu Y (2019) A review on the application of Bacillus as probiotics in aquaculture. Fish Shellfish Immunol 87:820–828. 10.1016/j.fsi.2019.02.010 30779995 10.1016/j.fsi.2019.02.010

[41] Liu L, Jiang D, Ren Y, Shi C, Wang Y, Yin J, Wang Q, Zhang D (2024) The Bacillus velezensis CYS06 strain exhibits promising applications in fighting grass carp bacterial diseases. Fishes 9:7. 10.3390/fishes9010007

[42] Monzón-Atienza L, Bravo J, Torrecillas S, Montero D, Canales AFG, de la Banda IG, Galindo-Villegas J, Ramos-Vivas J, Acosta F (2021) Isolation and characterization of a Bacillus velezensis D-18 Strain, as a potential probiotic in European seabass aquaculture. Probiotics Antimicrob Proteins 13(5):1404–1412. 10.1007/s12602-021-09782-8. (Epub 2021 Apr 3) 33811608 10.1007/s12602-021-09782-8

[43] Yi Y, Zhang Z, Zhao F, Liu H, Yu L, Zha J, Wang G (2018) Probiotic potential of *Bacillus velezensis* JW: antimicrobial activity against fish pathogenic bacteria and immune enhancement effects on Carassius auratus. Fish Shellfish Immunol 78:322–330. 10.1016/j.fsi.2018.04.055. (Epub 2018 Apr 25 PMID: 29702236) 29702236 10.1016/j.fsi.2018.04.055

[44] EFSA Panel on Biological Hazards (BIOHAZ), Koutsoumanis K, Allende A, Alvarez-Ordóñez A et al (2022) Update of the list of QPS-recommended biological agents intentionally added to food or feed as notified to EFSA 15: suitability of taxonomic units notified to EFSA until September 2021. EFSA J 20(1):e07045 35126735 10.2903/j.efsa.2022.7045 PMC8792879

[45] Ahluwalia B, Magnusson MK, Öhman L (2017) Mucosal immune system of the gastrointestinal tract: maintaining balance between the good and the bad. Scand J Gastroenterol 52:1185–1193. 10.1080/00365521.2017.1349173 28697651 10.1080/00365521.2017.1349173

[46] Wershil B, Furuta G (2008) 4. Gastrointestinal mucosal immunity. Journal of Allergy and Clinical Immunology 121:S380–S383. 10.1016/j.jaci.2007.10.023 18241686 10.1016/j.jaci.2007.10.023

[47] Eggers S, Safdar N, Sethi AK et al (2019) Urinary lead concentration and composition of the adult gut microbiota in a cross-sectional population-based sample. Environ Int 133:105122. 10.1016/j.envint.2019.105122 31518933 10.1016/j.envint.2019.105122 PMC7230144

[48] He C, Shan Y, Song W (2015) Targeting gut microbiota as a possible therapy for diabetes. Nutr Res 35:361–367. 10.1016/j.nutres.2015.03.002 25818484 10.1016/j.nutres.2015.03.002

[49] Khan MF, Wang H (2020) Environmental exposures and autoimmune diseases: contribution of gut microbiome. Front Immunol 10:3094. 10.3389/fimmu.2019.03094 31998327 10.3389/fimmu.2019.03094 PMC6970196

[50] Lu K, Knutson CG, Wishnok JS et al (2012) Serum metabolomics in a *Helicobacter hepaticus* mouse model of inflammatory bowel disease reveal important changes in the microbiome, Serum Peptides, and Intermediary Metabolism. J Proteome Res 11:4916–4926. 10.1021/pr300429x 22957933 10.1021/pr300429x PMC4026094

[51] Scordella G, Lumare F, Conides A, Papaconstantinou C. (2003) First occurrence of the Tilapia *Oreochromis niloticus* (Linnaeus, 1758) In Lesina Lagoon (Eastern Italian Coast). Medit Mar Sci 4:41. 10.12681/mms.239

[52] APHA (2005) Standard methods for the examination of water and wastewater, 21st edn. American Public Health Association/American Water Works Association/Water Environment Federation, Washington DC

[53] ISO 9308–3:1998 Water quality - detection and enumeration of Escherichia coli and coliform bacteria - Part 3: Miniaturized method (Most Probable Number) for the detection and enumeration of *E. coli* in surface and wastewater.

[54] APAT, IRSA-CNR (2003) Metodi analitici per le acque. Volume terzo. Sezione 7000 - Metodi per la determinazione di microorganismi indicatoridi inquinamento e di patogeni- 7080 *Salmonella* spp. 29. APAT, Manuali e Linee Guida, pp. 927–934.

[55] APAT, IRSA-CNR (2003) Metodi analitici per le acque. Volumeterzo. Sezione 7000 - Metodi per la determinazione di microorganismi indicatori di inquinamento e di patogeni- 7030 *Escherichia coli*. 29. APAT, Manuali e Linee Guida, pp. 883–894.

[56] ISO (2008) Microbiology of Food and Animal Feeding Stuffs – Horizontal method for the detection of *Salmonella* spp.(UNI EN ISO 6579: 2008). International Organization for Standardization, Geneva

[57] ISO 16649–3:2015 Microbiology of the food chain — horizontal method for the enumeration of beta-glucuronidase-positive *Escherichia coli* Part 3: Detection and most probable number technique using 5-bromo-4-chloro-3-indolyl-ß-D-glucuronide

[58] ISO 7218:2024 Microbiology of the food chain — general requirements and guidance for microbiological examinations.

[59] Oblinger JL, Koburger JA (1975) Understanding and teaching the most probable number technique 1. Journal of Milk and Food Technology 38:540–545. 10.4315/0022-2747-38.9.540

[60] Donovan TJ, Gallacher S, Andrews NJ et al (1998) Modification of the standard method used in the United Kingdom for counting *Escherichia coli* in live bivalve molluscs. Commun Dis Public Health 1:188–196 9782634

[61] ISO 4831:2006 Microbiology of food and animal feeding stuffs — horizontal method for the detection and enumeration of coliforms — most probable number technique.

[62] ISO 6579–1:2017/Amd 1:2020 Microbiology of the food chain — horizontal method for the detection, enumeration and serotyping of Salmonella — Part 1: Detection of *Salmonella* spp. 10.1016/j.ijfoodmicro.2018.03.022 29803313

[63] Stabili L, Licciano M, Giangrande A et al (2006) *Sabella spallanzanii* filter-feeding on bacterial community: ecological implications and applications. Mar Environ Res 61:74–92. 10.1016/j.marenvres.2005.06.001 16246412 10.1016/j.marenvres.2005.06.001

[64] ISO13720:2010 (2010) Meat and meat products — enumeration of presumptive *Pseudomonas* spp.

[65] ISO 16266:2006 (2006) Water quality — detection and enumeration of *Pseudomonas aeruginosa* — Method by membrane filtration.

[66] Marmur J. (1961). A procedure for the isolation of deoxyribonucleic acid from micro-organisms. Journal of molecular biology , 3 (2), 208-IN201.

[67] Raji AI, Möller C, Litthauer D, van Heerden E, Piater LA. (2008). Bacterial diversity of biofilm samples from deep mines in South Africa. Biokemistri , 20 (2).

[68] Guido A, Calcagnile M, Talà A et al (2024) Microbial consortium involved in ferromanganese and francolite biomineralization in an anchialine environment (Zinzulùsa Cave, Castro, Italy). Sci Total Environ 936:173423. 10.1016/j.scitotenv.2024.173423 38797412 10.1016/j.scitotenv.2024.173423

[69] Talà A, Buccolieri A, Calcagnile M et al (2021) Chemotrophic profiling of prokaryotic communities thriving on organic and mineral nutrients in a submerged coastal cave. Sci Total Environ 755:142514. 10.1016/j.scitotenv.2020.142514 33038840 10.1016/j.scitotenv.2020.142514

[70] Klindworth A, Pruesse E, Schweer T et al (2013) Evaluation of general 16S ribosomal RNA gene PCR primers for classical and next-generation sequencing-based diversity studies. Nucleic Acids Res 41:e1–e1. 10.1093/nar/gks808 22933715 10.1093/nar/gks808 PMC3592464

[71] Kolmogorov M, Yuan J, Lin Y, Pevzner PA (2019) Assembly of long, error-prone reads using repeat graphs. Nat Biotechnol 37:540–546. 10.1038/s41587-019-0072-8 30936562 10.1038/s41587-019-0072-8

[72] Simão FA, Waterhouse RM, Ioannidis P et al (2015) BUSCO: assessing genome assembly and annotation completeness with single copy orthologs. Bioinformatics 31:3210–3212. 10.1093/bioinformatics/btv351 26059717 10.1093/bioinformatics/btv351

[73] Gurevich A, Saveliev V, Vyahhi N, Tesler G (2013) QUAST: quality assessment tool for genome assemblies. Bioinformatics 29:1072–1075. 10.1093/bioinformatics/btt086 23422339 10.1093/bioinformatics/btt086 PMC3624806

[74] Seemann T (2014) Prokka: rapid prokaryotic genome annotation. Bioinformatics 30:2068–2069. 10.1093/bioinformatics/btu153 24642063 10.1093/bioinformatics/btu153

[75] Tanizawa Y, Fujisawa T, Nakamura Y (2018) DFAST: a flexible prokaryotic genome annotation pipeline for faster genome publication. Bioinformatics 34:1037–1039. 10.1093/bioinformatics/btx713 29106469 10.1093/bioinformatics/btx713 PMC5860143

[76] Jain M, Koren S, Miga KH et al (2018) Nanopore sequencing and assembly of a human genome with ultra-long reads. Nat Biotechnol 36:338–345. 10.1038/nbt.4060 29431738 10.1038/nbt.4060 PMC5889714

[77] Cosentino S, Voldby Larsen M, MÃ¸ller Aarestrup F, Lund O. (2013) Correction: pathogen finder - distinguishing friend from foe using bacterial whole genome sequence data. PLoS ONE 8:. 10.1371/annotation/b84e1af7-c127-45c3-be22-76abd977600f 10.1371/journal.pone.0077302 PMC3810466 24204795

[78] Cosentino S, Voldby Larsen M, Møller Aarestrup F, Lund O (2013) Pathogen finder - distinguishing friend from foe using bacterial whole genome sequence data. PLoS ONE 8:e77302. 10.1371/journal.pone.0077302 24204795 10.1371/journal.pone.0077302 PMC3810466

[79] Malberg Tetzschner AM, Johnson JR, Johnston BD et al (2020) *In Silico* genotyping of *Escherichia coli* isolates for extraintestinal virulence genes by use of whole-genome sequencing data. J Clin Microbiol 58:e01269-e1320. 10.1128/JCM.01269-20 32669379 10.1128/JCM.01269-20 PMC7512150

[80] Alcock BP, Huynh W, Chalil R et al (2023) CARD 2023: expanded curation, support for machine learning, and resistome prediction at the comprehensive antibiotic resistance database. Nucleic Acids Res 51:D690–D699. 10.1093/nar/gkac920 36263822 10.1093/nar/gkac920 PMC9825576

[81] Bortolaia V, Kaas RS, Ruppe E et al (2020) ResFinder 4.0 for predictions of phenotypes from genotypes. J Antimicrob Chemother 75:3491–3500. 10.1093/jac/dkaa345 32780112 10.1093/jac/dkaa345 PMC7662176

[82] Gschwind R, Ugarcina Perovic S, Weiss M et al (2023) ResFinderFG v2.0: a database of antibiotic resistance genes obtained by functional metagenomics. Nucleic Acids Res 51:W493–W500. 10.1093/nar/gkad384 37207327 10.1093/nar/gkad384 PMC10320180

[83] Biswas A, Staals RHJ, Morales SE et al (2016) CRISPRDetect: a flexible algorithm to define CRISPR arrays. BMC Genomics 17:356. 10.1186/s12864-016-2627-0 27184979 10.1186/s12864-016-2627-0 PMC4869251

[84] Couvin D, Bernheim A, Toffano-Nioche C et al (2018) CRISPRCasFinder, an update of CRISRFinder, includes a portable version, enhanced performance and integrates search for Cas proteins. Nucleic Acids Res 46:W246–W251. 10.1093/nar/gky425 29790974 10.1093/nar/gky425 PMC6030898

[85] Carattoli A, Zankari E, García-Fernández A et al (2014) *In silico* detection and typing of plasmids using plasmid finder and plasmid multilocus sequence typing. Antimicrob Agents Chemother 58:3895–3903. 10.1128/AAC.02412-14 24777092 10.1128/AAC.02412-14 PMC4068535

[86] Johansson MHK, Bortolaia V, Tansirichaiya S et al (2021) Detection of mobile genetic elements associated with antibiotic resistance in *Salmonella enterica* using a newly developed web tool: MobileElementFinder. J Antimicrob Chemother 76:101–109. 10.1093/jac/dkaa390 33009809 10.1093/jac/dkaa390 PMC7729385

[87] Gan R, Zhou F, Si Y et al (2022) DBSCAN-SWA: An integrated tool for rapid prophage detection and annotation. Front Genet 13:885048. 10.3389/fgene.2022.885048 35518360 10.3389/fgene.2022.885048 PMC9061938

[88] Wishart DS, Han S, Saha S et al (2023) PHASTEST: faster than PHASTER, better than PHAST. Nucleic Acids Res 51:W443–W450. 10.1093/nar/gkad382 37194694 10.1093/nar/gkad382 PMC10320120

[89] Blin K, Shaw S, Augustijn HE et al (2023) antiSMASH 7.0: new and improved predictions for detection, regulation, chemical structures and visualisation. Nucleic Acids Res 51:W46–W50. 10.1093/nar/gkad344 37140036 10.1093/nar/gkad344 PMC10320115

[90] Kebbi-Beghdadi C, Greub G (2014) Importance of amoebae as a tool to isolate amoeba-resisting microorganisms and for their ecology and evolution: the *C.** hlamydia* paradigm. Environ Microbiol Rep 6:309–324. 10.1111/1758-2229.12155 24992529 10.1111/1758-2229.12155

[91] Akanuma G, Nanamiya H, Natori Y et al (2012) Inactivation of ribosomal protein genes in *Bacillus subtilis* reveals importance of each ribosomal protein for cell proliferation and cell differentiation. J Bacteriol 194:6282–6291. 10.1128/JB.01544-12 23002217 10.1128/JB.01544-12 PMC3486396

[92] Shoji S, Dambacher CM, Shajani Z et al (2011) Systematic chromosomal deletion of bacterial ribosomal protein genes. J Mol Biol 413:751–761. 10.1016/j.jmb.2011.09.004 21945294 10.1016/j.jmb.2011.09.004 PMC3694390

[93] Allison SE, D’Elia MA, Arar S et al (2011) Studies of the genetics, function, and kinetic mechanism of *tagE,* the wall teichoic acid glycosyltransferase in *Bacillus subtilis* 168. J Biol Chem 286:23708–23716. 10.1074/jbc.M111.241265 21558268 10.1074/jbc.M111.241265 PMC3129151

[94] Magnuson R, Solomon J, Grossman AD (1994) Biochemical and genetic characterization of a competence pheromone from *B. subtilis*. Cell 77:207–216. 10.1016/0092-8674(94)90313-1 8168130 10.1016/0092-8674(94)90313-1

[95] Unden G, Klein R (2021) Sensing of O_2_ and nitrate by bacteria: alternative strategies for transcriptional regulation of nitrate respiration by O_2_ and nitrate. Environ Microbiol 23:5–14. 10.1111/1462-2920.15293 33089915 10.1111/1462-2920.15293

[96] Stephenson S, Mueller C, Jiang M, Perego M (2003) Molecular analysis of Phr peptide processing in *Bacillus subtilis*. J Bacteriol 185:4861–4871. 10.1128/JB.185.16.4861-4871.2003 12897006 10.1128/JB.185.16.4861-4871.2003 PMC166482

[97] Siegers K, Entian KD (1995) Genes involved in immunity to the lantibiotic nisin produced by *Lactococcus lactis* 6F3. Appl Environ Microbiol 61:1082–1089. 10.1128/aem.61.3.1082-1089.1995 7793910 10.1128/aem.61.3.1082-1089.1995 PMC167363

[98] Erega A, Stefanic P, Dogsa I et al (2021) Bacillaene mediates the inhibitory effect of *Bacillus subtilis* on *Campylobacter jejuni* biofilms. Appl Environ Microbiol 87:e02955-e3020. 10.1128/AEM.02955-20 33837012 10.1128/AEM.02955-20 PMC8174767

[99] Butcher RA, Schroeder FC, Fischbach MA et al (2007) The identification of bacillaene, the product of the PksX megacomplex in *Bacillus subtilis*. Proc Natl Acad Sci USA 104:1506–1509. 10.1073/pnas.0610503104 17234808 10.1073/pnas.0610503104 PMC1785240

[100] Patel PS, Huang S, Fisher S et al (1995) Bacillaene, a novel inhibitor of procaryotic protein synthesis produced by *Bacillus subtilis*: production, taxonomy, isolation, physico-chemical characterization and biological activity. J Antibiot 48:997–1003. 10.7164/antibiotics.48.997 10.7164/antibiotics.48.997 7592068

[101] Dimopoulou A, Theologidis I, Benaki D, et al. (2021) Direct antibiotic activity of Bacillibactin broadens the biocontrol range of *Bacillus amyloliquefaciens* MBI600. mSphere 6:e00376–21. 10.1128/mSphere.00376-21 10.1128/mSphere.00376-21 PMC8386435 34378986

[102] Chakraborty K, Kizhakkekalam VK, Joy M, Chakraborty RD (2022) Bacillibactin class of siderophore antibiotics from a marine symbiotic Bacillus as promising antibacterial agents. Appl Microbiol Biotechnol 106:329–340. 10.1007/s00253-021-11632-0 34913995 10.1007/s00253-021-11632-0

[103] Kenig M, Abraham EP (1976) Antimicrobial activities and antagonists of bacilysin and anticapsin. J Gen Microbiol 94:37–45. 10.1099/00221287-94-1-37 819623 10.1099/00221287-94-1-37

[104] Wu L, Wu H, Chen L et al (2014) Bacilysin from *Bacillus amyloliquefaciens* FZB42 has specific bactericidal activity against harmful algal bloom species. Appl Environ Microbiol 80:7512–7520. 10.1128/AEM.02605-14 25261512 10.1128/AEM.02605-14 PMC4249228

[105] Barale SS, Ghane SG, Sonawane KD (2022) Purification and characterization of antibacterial surfactin isoforms produced by *Bacillus velezensis* SK. AMB Expr 12:7. 10.1186/s13568-022-01348-3 10.1186/s13568-022-01348-3 PMC8795249 35084596

[106] Nitschke M, Araújo LV, Costa SGVAO et al (2009) Surfactin reduces the adhesion of food-borne pathogenic bacteria to solid surfaces. Lett Appl Microbiol 49:241–247. 10.1111/j.1472-765X.2009.02646.x 19486287 10.1111/j.1472-765X.2009.02646.x

[107] Horng Y-B, Yu Y-H, Dybus A et al (2019) Antibacterial activity of Bacillus species-derived surfactin on *Brachyspira hyodysenteriae* and *Clostridium perfringens*. AMB Expr 9:188. 10.1186/s13568-019-0914-2 10.1186/s13568-019-0914-2 PMC6872690 31754906

[108] Wilson KE, Flor JE, Schwartz RE et al (1987) Difficidin and oxydifficidin: novel broad spectrum antibacterial antibiotics produced by *Bacillus subtilis*. II. Isolation and physico-chemical characterization J Antibiot 40:1682–1691. 10.7164/antibiotics.40.1682 10.7164/antibiotics.40.1682 3429336

[109] Zhang L, Sun C (2018) Fengycins, cyclic lipopeptides from marine *Bacillus subtilis* strains, kill the plant-pathogenic fungus *Magnaporthe grisea* by inducing reactive oxygen species production and chromatin condensation. Appl Environ Microbiol 84:e00445-e518. 10.1128/AEM.00445-18 29980550 10.1128/AEM.00445-18 PMC6122000

[110] Vanittanakom N, Loeffler W, Koch U, Jung G (1986) Fengycin - a novel antifungal lipopeptide antibiotic produced by *Bacillus subtilis* F-29-3. J Antibiot 39:888–901. 10.7164/antibiotics.39.888 10.7164/antibiotics.39.888 3093430

[111] Karpiński TM (2019) Marine macrolides with antibacterial and/or antifungal activity. Mar Drugs 17:241. 10.3390/md17040241 31018512 10.3390/md17040241 PMC6520931

[112] Molohon KJ, Saint-Vincent PMB, Park S et al (2016) Plantazolicin is an ultranarrow-spectrum antibiotic that targets the *Bacillus anthracis* membrane. ACS Infect Dis 2:207–220. 10.1021/acsinfecdis.5b00115 27152321 10.1021/acsinfecdis.5b00115 PMC4852872

[113] Kruszewska D, Sahl HG, Bierbaum G et al (2004) Mersacidin eradicates methicillin-resistant *Staphylococcus aureus* (MRSA) in a mouse rhinitis model. J Antimicrob Chemother 54:648–653. 10.1093/jac/dkh387 15282239 10.1093/jac/dkh387

[114] Brötz H, Bierbaum G, Leopold K et al (1998) The lantibiotic mersacidin inhibits peptidoglycan synthesis by targeting lipid II. Antimicrob Agents Chemother 42:154–160. 10.1128/AAC.42.1.154 9449277 10.1128/aac.42.1.154 PMC105472

[115] Niu WW, Neu HC (1991) Activity of mersacidin, a novel peptide, compared with that of vancomycin, teicoplanin, and daptomycin. Antimicrob Agents Chemother 35:998–1000. 10.1128/AAC.35.5.998 1649577 10.1128/aac.35.5.998 PMC245145

[116] Tran NT, Zhang J, Xiong F et al (2018) Altered gut microbiota associated with intestinal disease in grass carp (*Ctenopharyngodon idellus*). World J Microbiol Biotechnol 34:71. 10.1007/s11274-018-2447-2 29777414 10.1007/s11274-018-2447-2

[117] Dias SDC, Costa LRM, Buiatte ABG, et al. (2024) Escherichia coli as a sentinel in the assessment of antimicrobial resistance in the tilapia production chain: from production environment to the final product. Front Antibiot. 3:1461662. Published 10.3389/frabi.2024.1461662 10.3389/frabi.2024.1461662 PMC11731651 39816247

[118] Cohen D, Treygerman O, Ken-Dror S et al (2024) Twenty-five years of sentinel laboratory-based surveillance of shigellosis in a high-income country endemic for the disease, Israel, 1998 to 2022. Euro Surveill 29(31):2400022. 10.2807/1560-7917.ES.2024.29.31.2400022 39092530 10.2807/1560-7917.ES.2024.29.31.2400022 PMC11295440

[119] Fečkaninová A, Koščová J, Mudroňová D et al (2017) The use of probiotic bacteria against Aeromonas infections in salmonid aquaculture. Aquaculture 469:1–8. 10.1016/j.aquaculture.2016.11.042

[120] Park SY, Han JE, Kwon H et al (2020) Recent insights into *Aeromonas salmonicida* and its Bacteriophages in aquaculture: a comprehensive review. J Microbiol Biotechnol 30:1443–1457. 10.4014/jmb.2005.05040 32807762 10.4014/jmb.2005.05040 PMC9728264

[121] Li J, Fang P, Yi X, et al. (2022) Probiotics *Bacillus cereus* and *B. subtilis* reshape the intestinal microbiota of Pengze crucian carp (*Carassius auratus* var. Pengze) fed with high plant protein diets. Front Nutr 9:1027641. 10.3389/fnut.2022.1027641 10.3389/fnut.2022.1027641 PMC9627213 36337612

[122] Butt RL, Volkoff H (2019) Gut microbiota and energy homeostasis in fish. Front Endocrinol 10:9. 10.3389/fendo.2019.00009 10.3389/fendo.2019.00009 PMC6353785 30733706

[123] Gerritsen J, Hornung B, Renckens B et al (2017) Genomic and functional analysis of *Romboutsia ilealis* CRIB^T^ reveals adaptation to the small intestine. PeerJ 5:e3698. 10.7717/peerj.3698 28924494 10.7717/peerj.3698 PMC5598433

[124] Han Z, Sun J, Jiang B et al (2024) Fecal microbiota transplantation accelerates restoration of florfenicol-disturbed intestinal microbiota in a fish model. Commun Biol 7:1006. 10.1038/s42003-024-06727-z 39152200 10.1038/s42003-024-06727-z PMC11329668

[125] Wang Q, Wang K, Wu W et al (2020) Administration of Bifidobacterium bifidum CGMCC 15068 modulates gut microbiota and metabolome in azoxymethane (AOM)/dextran sulphate sodium (DSS)-induced colitis-associated colon cancer (CAC) in mice. Appl Microbiol Biotechnol 104:5915–5928. 10.1007/s00253-020-10621-z 32367312 10.1007/s00253-020-10621-z

[126] Tsuchiya C, Sakata T, Sugita H. (2007) Novel ecological niche of *Cetobacterium somerae*, an anaerobic bacterium in the intestinal tracts of freshwater fish. Lett Appl Microbiol 0:071018031740002-??? 10.1111/j.1472-765X.2007.02258.x 10.1111/j.1472-765X.2007.02258.x 17944860

[127] Van Kessel MA, Dutilh BE, Neveling K, et al. (2011) Pyrosequencing of 16S rRNA gene amplicons to study the microbiota in the gastrointestinal tract of carp (*Cyprinus carpio* L.). AMB Expr 1:41. 10.1186/2191-0855-1-41 10.1186/2191-0855-1-41 PMC3226434 22093413

[128] Ramírez C, Coronado J, Silva A, Romero J (2018) Cetobacterium is a major component of the microbiome of giant Amazonian fish (*Arapaima gigas*) in Ecuador. Animals 8:189. 10.3390/ani8110189 30352962 10.3390/ani8110189 PMC6262583

[129] Larsen AM, Mohammed HH, Arias CR (2014) Characterization of the gut microbiota of three commercially valuable warmwater fish species. J Appl Microbiol 116:1396–1404. 10.1111/jam.12475 24529218 10.1111/jam.12475

[130] Sugita H, Miyajima C, Deguchi Y (1991) The vitamin B12-producing ability of the intestinal microflora of freshwater fish. Aquaculture 92:267–276. 10.1016/0044-8486(91)90028-6

[131] Kudo T, Hatai K, Seino A (1988) *Nocardia seriolae* sp. nov. causing nocardiosis of cultured fish. Int J Syst Bacteriol 38:173–178. 10.1099/00207713-38-2-173

[132] Maekawa S, Yoshida T, Wang P, Chen S (2018) Current knowledge of nocardiosis in teleost fish. J Fish Dis 41:413–419. 10.1111/jfd.12782 29341219 10.1111/jfd.12782

[133] Labrie L, Ng J, Tan Z, et al. (2008) Nocardial infections in fish: an emerging problem in both freshwater and marine aquaculture systems in Asia. In: Diseases in Asian Aquaculture VI. pp 297–312

[134] Medina-Felix D, Vargas-Albores F, Garibay-Valdez E, et al. (2024) Gastrointestinal dysbiosis induced by Nocardia sp. infection in tilapia. Comparative Biochemistry and Physiology Part D: Genomics and Proteomics 49:101154. 10.1016/j.cbd.2023.101154 10.1016/j.cbd.2023.101154 37976964

[135] Wang X, Onchari MM, Yang X et al (2022) Genome analysis of *Bacillus subtilis* JCL16 and the synergistic relationship among its metabolites reveal its potential for biocontrol of Nocardia seriolae. Biol Control 167:104855. 10.1016/j.biocontrol.2022.104855

[136] Ishikawa C. (2001) Fish mycobacteriosis. BOLETIM DO INSTITUTO DE PESCA 27:

[137] Ringø E, Li X, Doan HV, Ghosh K (2022) Interesting probiotic Bacteria other than the more widely used lactic acid Bacteria and Bacilli in Finfish. Front Mar Sci 9:848037. 10.3389/fmars.2022.848037

[138] Sharifuzzaman SM, Abbass A, Tinsley JW, Austin B (2011) Subcellular components of probiotics Kocuria SM1 and Rhodococcus SM2 induce protective immunity in rainbow trout (Oncorhynchus mykiss, Walbaum) against *Vibrio anguillarum*. Fish Shellfish Immunol 30:347–353. 10.1016/j.fsi.2010.11.005 21078398 10.1016/j.fsi.2010.11.005

[139] Boutin S, Audet C, Derome N (2013) Probiotic treatment by indigenous bacteria decreases mortality without disturbing the natural microbiota of *Salvelinus fontinalis*. Can J Microbiol 59:662–670. 10.1139/cjm-2013-0443 24102219 10.1139/cjm-2013-0443

[140] Garai E, Risa A, Varga E et al (2021) Evaluation of the multimycotoxin-degrading Efficiency of *Rhodococcus erythropolis* NI1 strain with the three-Step Zebrafish Microinjection Method. Int J Mol Sci 22:724. 10.3390/ijms22020724 33450918 10.3390/ijms22020724 PMC7828439

[141] Butt UD, Khan S, Liu X et al (2024) Present status, limitations, and prospects of using Streptomyces bacteria as a potential probiotic agent in aquaculture. Probiotics & Antimicro Prot 16:426–442. 10.1007/s12602-023-10053-x 10.1007/s12602-023-10053-x PMC10024021 36933159

[142] James G, Prasannan Geetha P, Thavarool Puthiyedathu S, Vattringal Jayadradhan R. (2023) Applications of actinobacteria in aquaculture: prospects and challenges. 3 Biotech 13:42. 10.1007/s13205-023-03465-7 10.1007/s13205-023-03465-7 PMC9834454 36643400

[143] Aktuganov G, Melentjev A, Galimzianova N et al (2008) Wide-range antifungal antagonism of *Paenibacillus ehimensis* IB-X-b and its dependence on chitinase and β-1,3-glucanase production. Can J Microbiol 54:577–587. 10.1139/W08-043 18641704 10.1139/w08-043

[144] Lin PH, Chen SW, Wen ZH, Hu SY (2022) Administration of the potential probiotic *Paenibacillus ehimensis* NPUST1 enhances expression of indicator genes associated with nutrient metabolism, growth and innate immunity against *Aeromonas hydrophila* and *Streptococcus indie* infections in Zebrafish (*Danio rerio*). Fishes 7:386. 10.3390/fishes7060386

[145] Midhun SJ, Neethu S, Vysakh A et al (2017) Antibacterial activity and probiotic characterization of autochthonous *Paenibacillus polymyxa* isolated from Anabas testudineus (Bloch, 1792). Microb Pathog 113:403–411. 10.1016/j.micpath.2017.11.019 29146501 10.1016/j.micpath.2017.11.019

[146] Olishevska S, Nickzad A, Déziel E (2019) Bacillus and Paenibacillus secreted polyketides and peptides involved in controlling human and plant pathogens. Appl Microbiol Biotechnol 103:1189–1215. 10.1007/s00253-018-9541-0 30603850 10.1007/s00253-018-9541-0

[147] Chen SW, Liu CH, Hu SY (2019) Dietary administration of probiotic *Paenibacillus ehimensis* NPUST1 with bacteriocin-like activity improves growth performance and immunity against *Aeromonas hydrophila* and *Streptococcus iniae* in Nile tilapia (*Oreochromis niloticus*). Fish Shellfish Immunol 84:695–703. 10.1016/j.fsi.2018.10.059 30368025 10.1016/j.fsi.2018.10.059

[148] Shinn AP, Avenant-Oldewage A, Bondad-Reantaso MG et al (2023) A global review of problematic and pathogenic parasites of farmed tilapia. Rev Aquac 15:92–153. 10.1111/raq.12742

[149] FAO/WHO. Probiotics in food: health and nutritional properties and guidelines for evaluation food and agriculture organization of the United Nations, Rome, Italy (2006)

[150] EFSA panel on additives and products or substances used in animal feed (FEEDAP) Rychen G, Aquilina G, Azimonti G, Bampidis V, Bastos MdL, Bories G, Chesson A, Cocconcelli PS, Flachowsky G, et al. Guidance on the characterisation of microorganisms used as feed additives or as production organisms. EFSA J. 2018;16:e05206. 10.2903/j.efsa.2018.5206. 10.2903/j.efsa.2018.5206 PMC7009341 32625840

[151] Peng X, Ed-Dra A, Yue M. (2023) Whole genome sequencing for the risk assessment of probiotic lactic acid bacteria. Crit Rev Food Sci Nutr. 63(32):11244–11262. 10.1080/10408398.2022.2087174. Epub 2022 Jun 13. PMID: 35694810. 10.1080/10408398.2022.2087174 35694810

[152] Akanuma G, Kobayashi A, Suzuki S et al (2014) Defect in the formation of 70S ribosomes caused by lack of ribosomal protein L34 can be suppressed by magnesium. J Bacteriol 196:3820–3830. 10.1128/JB.01896-14 25182490 10.1128/JB.01896-14 PMC4248831

[153] Akanuma G, Yamazaki K, Yagishi Y, et al. (2018) Magnesium suppresses defects in the formation of 70S ribosomes as well as in sporulation caused by lack of several individual ribosomal proteins. J Bacteriol 200:. 10.1128/JB.00212-18 10.1128/JB.00212-18 PMC6112008 29967120

[154] Lin YH, Ku CY, Shiau SY (2013) Estimation of dietary magnesium requirements of juvenile tilapia, Oreochromis niloticus× Oreochromis aureus, reared in freshwater and seawater. Aquaculture 380:47–51

[155] Honeyman AL, Stewart GC (1989) The nucleotide sequence of the *rodC* operon of *Bacillus subtilis*. Mol Microbiol 3:1257–1268. 10.1111/j.1365-2958.1989.tb00276.x 2507871 10.1111/j.1365-2958.1989.tb00276.x

[156] Young FE (1967) Requirement of glucosylated teichoic acid for adsorption of phage in *Bacillus subtilis* 168. Proc Natl Acad Sci USA 58:2377–2384. 10.1073/pnas.58.6.2377 4969329 10.1073/pnas.58.6.2377 PMC223846

[157] Young FE, Smith C, Reilly BE (1969) Chromosomal location of genes regulating resistance to Bacteriophage in *Bacillus subtilis*. J Bacteriol 98:1087–1097. 10.1128/jb.98.3.1087-1097.1969 4977981 10.1128/jb.98.3.1087-1097.1969 PMC315300

[158] Zhang Z, Liang L, Li D et al (2023) *Bacillus subtilis* phage phi18: genomic analysis and receptor identification. Arch Virol 168:17. 10.1007/s00705-022-05686-2 36593367 10.1007/s00705-022-05686-2

[159] Soundararajan M, von Bünau R, Oelschlaeger TA. (2019) K5 capsule and lipopolysaccharide are important in resistance to T4 phage attack in probiotic *E. coli* Strain Nissle 1917. Front Microbiol. 10:2783. 10.3389/fmicb.2019.02783. 10.3389/fmicb.2019.02783 PMC6895014 31849915

[160] Nagarajan V, Peng M, Tabashsum Z, Salaheen S, Padilla J, Biswas D (2019) Antimicrobial effect and probiotic potential of phage resistant *Lactobacillus plantarum* and its interactions with zoonotic bacterial pathogens. Foods 8(6):194. 10.3390/foods8060194 31195676 10.3390/foods8060194 PMC6616511

[161] Sass P, Jansen A, Szekat C, Sass V, Sahl HG, Bierbaum G (2008) The lantibiotic mersacidin is a strong inducer of the cell wall stress response of Staphylococcus aureus. BMC Microbiol 8:186. 10.1186/1471-2180-8-186 18947397 10.1186/1471-2180-8-186 PMC2592248

[162] Chatterjee S, Chatterjee DK, Jani RH, Blumbach J, Ganguli BN, Klesel N, Limbert M, Seibert G. (1992) Mersacidin, a new antibiotic from Bacillus. In vitro and in vivo antibacterial activity. J Antibiot (Tokyo). 45(6):839–45. 10.7164/antibiotics.45.839. 10.7164/antibiotics.45.839 1500348

[163] Brötz H, Bierbaum G, Markus A, Molitor E, Sahl HG (1995) Mode of action of the lantibiotic mersacidin: inhibition of peptidoglycan biosynthesis via a novel mechanism? Antimicrob Agents Chemother 39(3):714–719. 10.1128/AAC.39.3.714 7793878 10.1128/AAC.39.3.714 PMC162610

[164] Commission Regulation (EC) No 429/2008 of 25 April 2008 on detailed rules for the implementation of Regulation (EC) No 1831/2003 of the European Parliament and of the Council as regards the preparation and the presentation of applications and the assessment and the authorisation of feed additives.

