# Supplementary material for: Effect of Bacillus velezensis MT9 on Nile Tilapia (Oreochromis Niloticus) Intestinal Microbiota
Source: Microb Ecol. 2025 May 1;88:37. doi: 10.1007/s00248-025-02531-2 (PMC12045831; doi:10.1007/s00248-025-02531-2)
Supplement: Supplementary file 1 [file 248_2025_2531_MOESM16_ESM.docx]

**Table S1.** Number of reads and quality control.

| **Mean read length** | **Mean read quality** | **Median read length** | **Median read quality** | **Number of reads** | **Read length N50** |
| --- | --- | --- | --- | --- | --- |
| 2,332.90 | 19.3 | 2,275.00 | 23.4 | 3,689,740.00 | 2,870.00 |
