# Supplementary material for: Effect of Bacillus velezensis MT9 on Nile Tilapia (Oreochromis Niloticus) Intestinal Microbiota
Source: Microb Ecol. 2025 May 1;88:37. doi: 10.1007/s00248-025-02531-2 (PMC12045831; doi:10.1007/s00248-025-02531-2)
Supplement: Supplementary file 2 [file 248_2025_2531_MOESM17_ESM.docx]

**Table S2.** BUSCO statistics.

| Complete BUSCOs (C) | 124 |
| --- | --- |
| Complete and single-copy BUSCOs (S) | 124 |
| Complete and duplicated BUSCOs (D) | 0 |
| Fragmented BUSCOs (F) | 0 |
| Missing BUSCOs (M) | 0 |
| Total BUSCO groups searched | 124 |
| Number of scaffolds | 1 |
| Number of contigs | 1 |
| Total length | 4,139,342 |
| Percent gaps | 0.00% |
| Scaffold N50 | 4 MB |
| Contigs N50 | 4 MB |
