# Supplementary figures and images for: Effect of Bacillus velezensis MT9 on Nile Tilapia (Oreochromis Niloticus) Intestinal Microbiota
Source: Microb Ecol. 2025 May 1;88(1):37. doi: 10.1007/s00248-025-02531-2 (PMC12045831; doi:10.1007/s00248-025-02531-2)

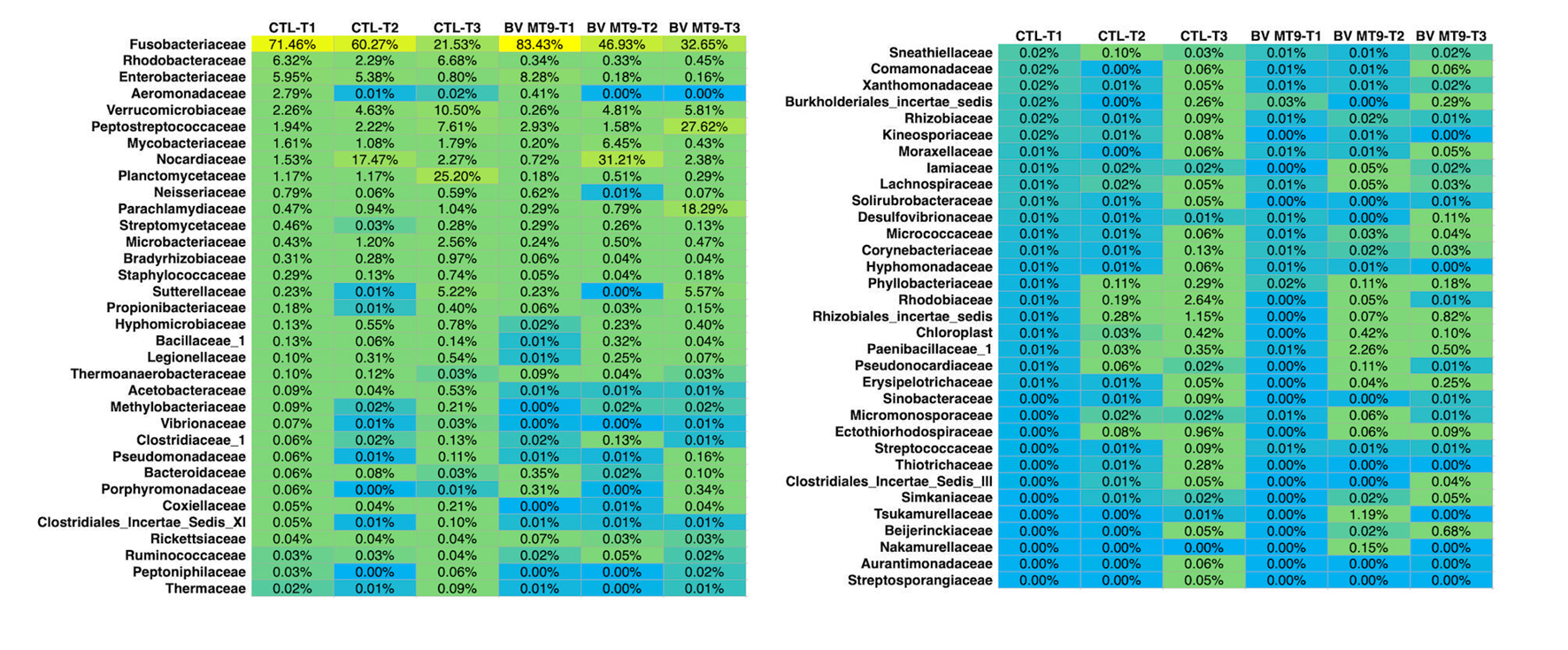

Supplement: Supplementary file 1 — (PNG 1.35 MB) [file 248_2025_2531_Fig11_ESM.png]

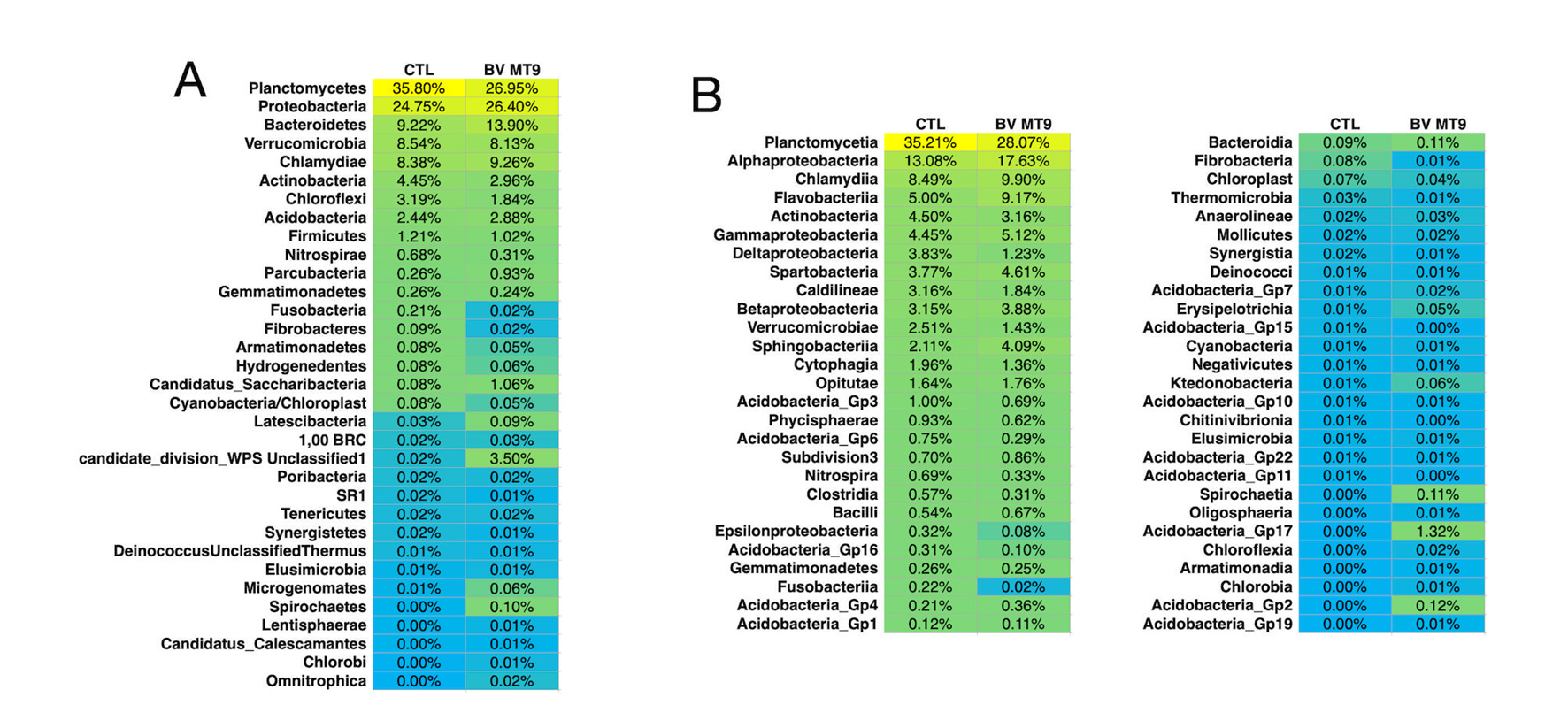

Supplement: Supplementary file 3 — (PNG 965 KB) [file 248_2025_2531_Fig12_ESM.png]

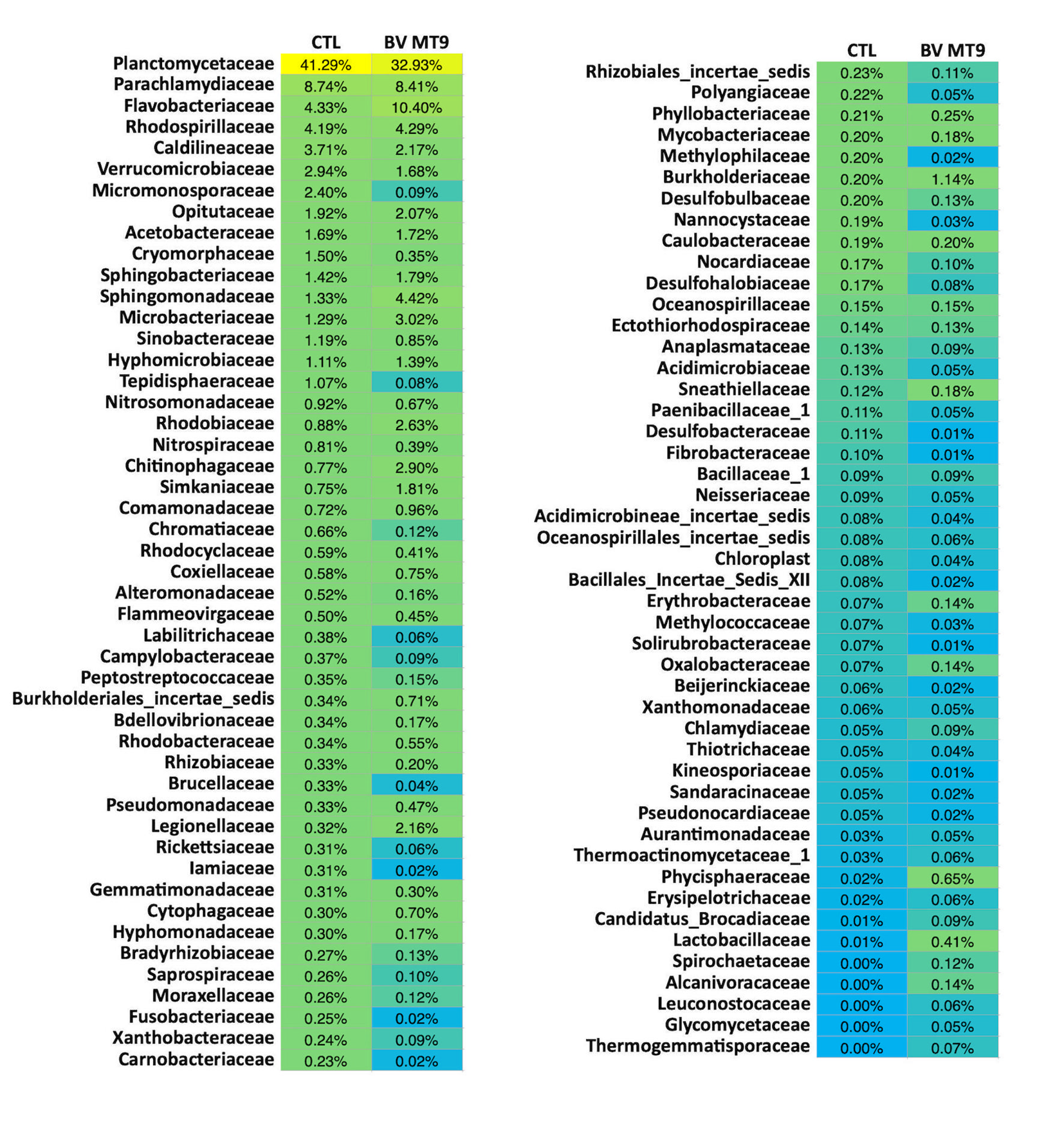

Supplement: Supplementary file 5 — (PNG 2.07 MB) [file 248_2025_2531_Fig13_ESM.png]

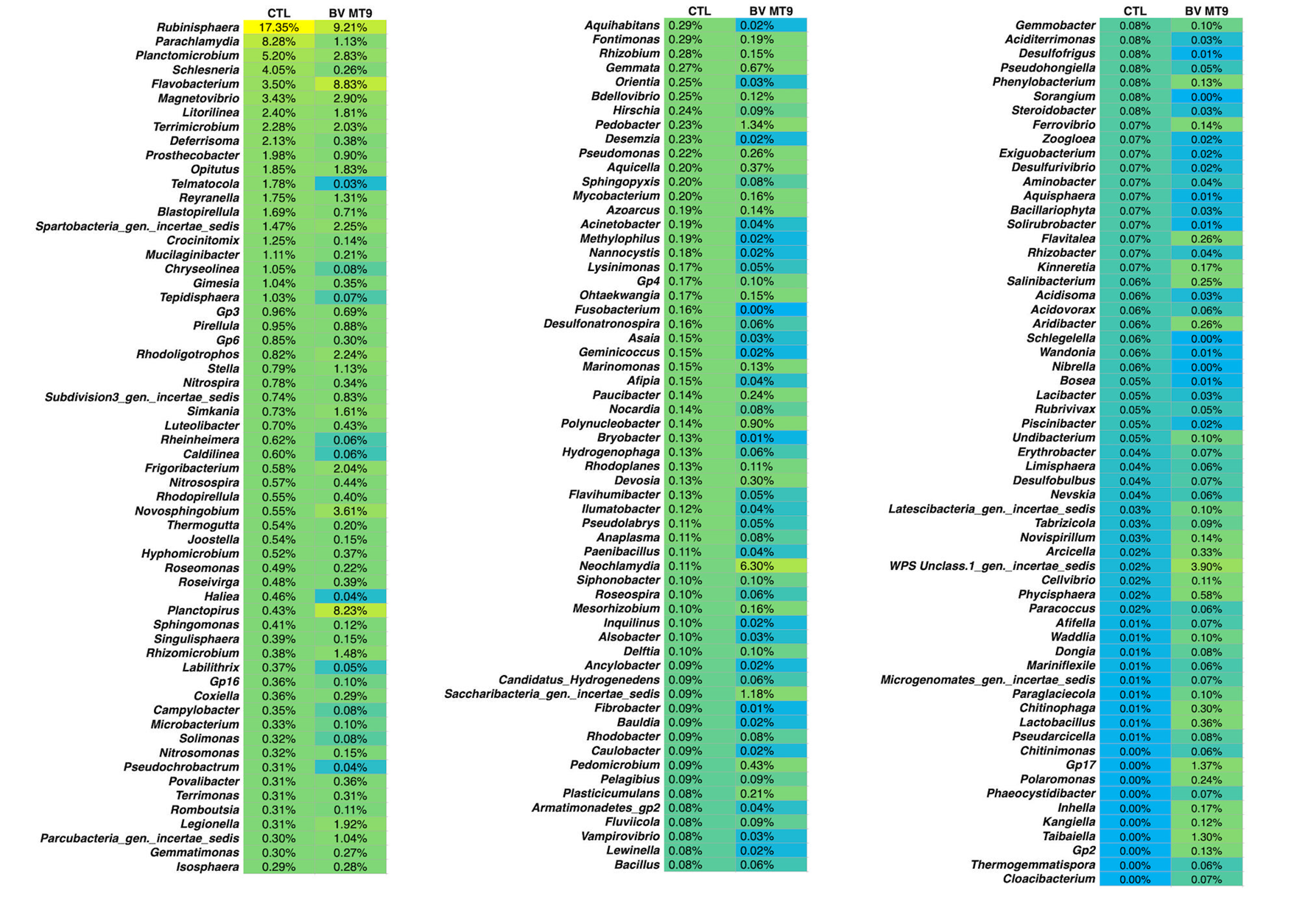

Supplement: Supplementary file 7 — (PNG 1.61 MB) [file 248_2025_2531_Fig14_ESM.png]

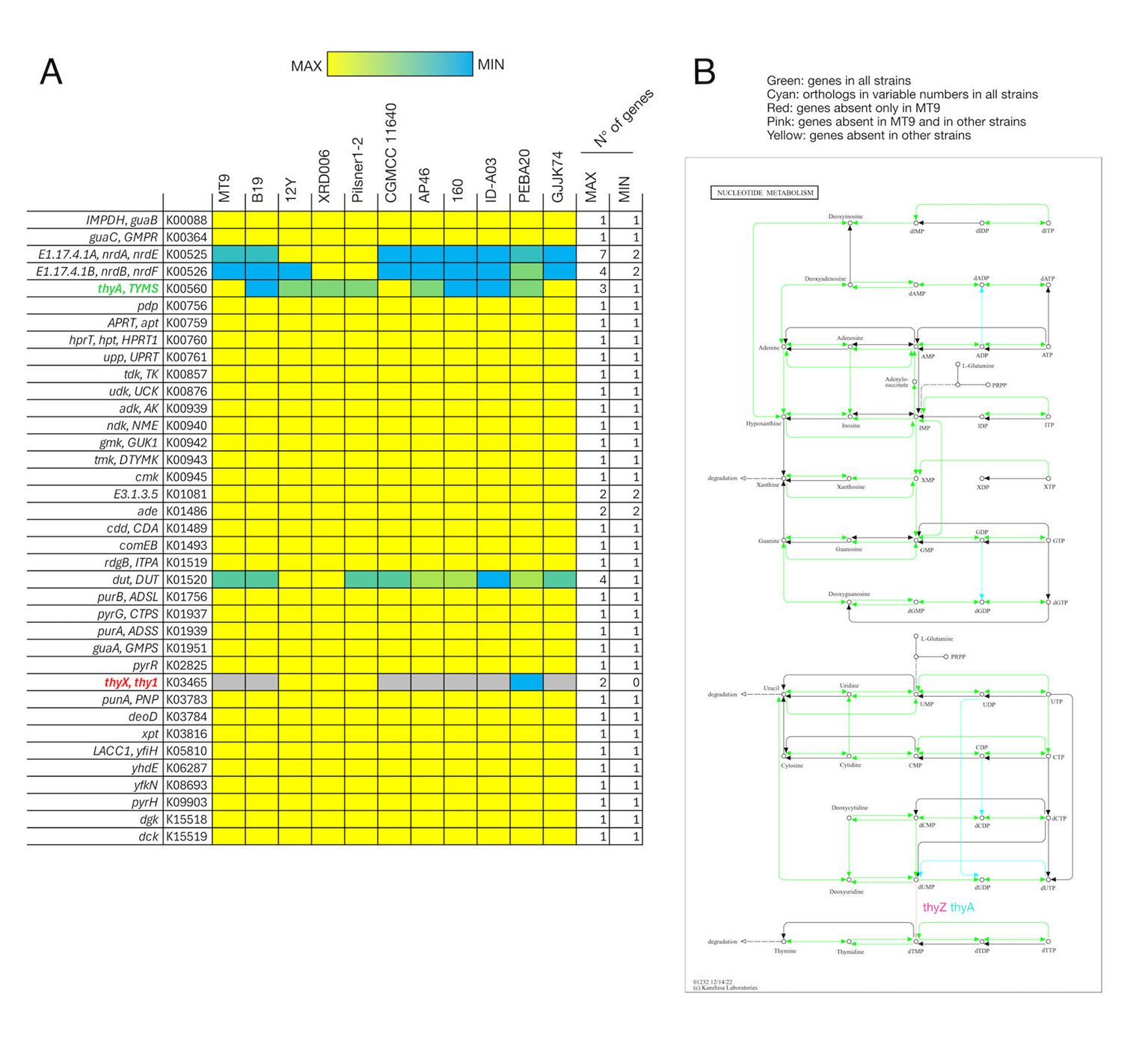

Supplement: Supplementary file 9 — (PNG 528 KB) [file 248_2025_2531_Fig15_ESM.png]

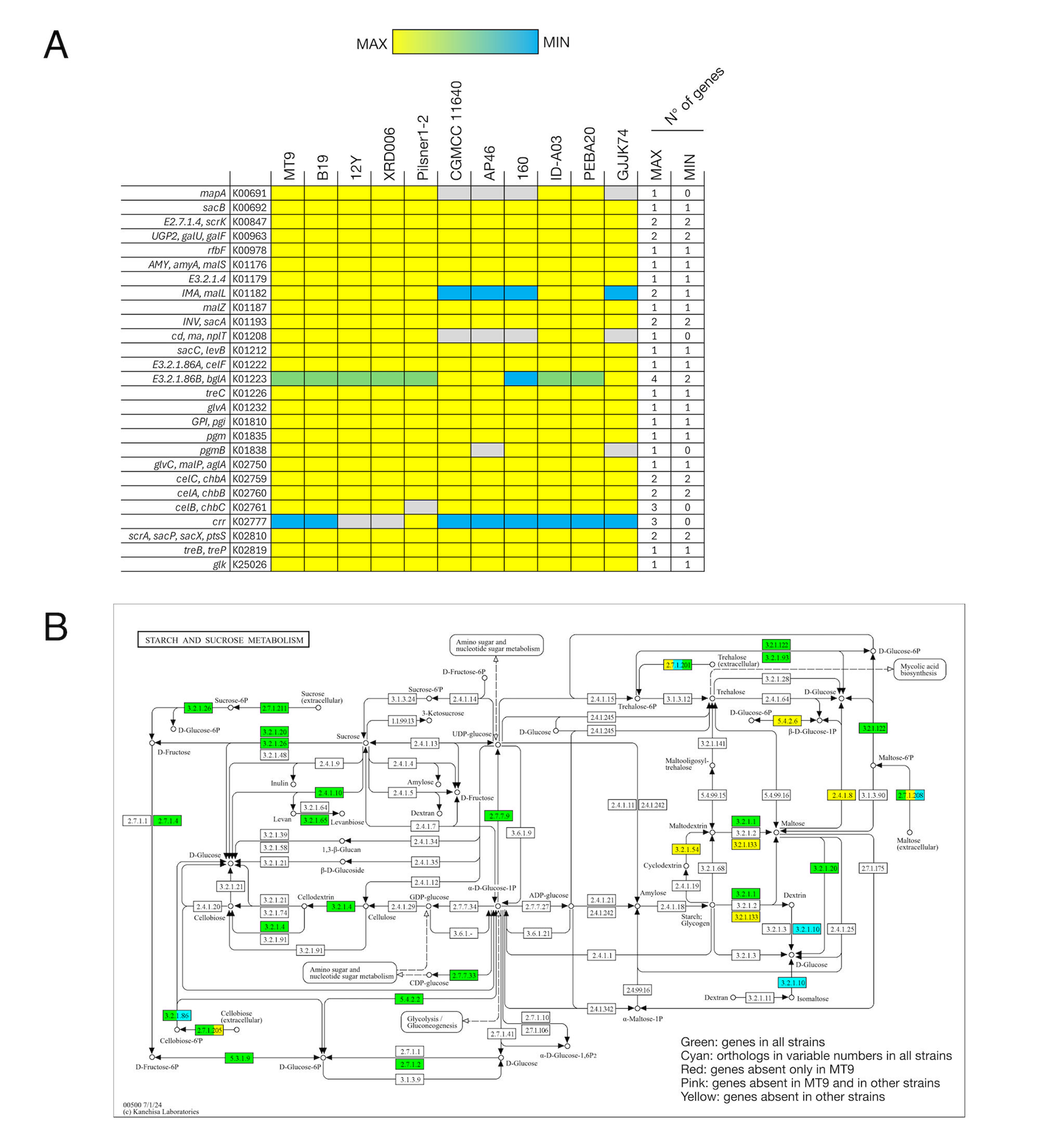

Supplement: Supplementary file 11 — (PNG 784 KB) [file 248_2025_2531_Fig16_ESM.png]

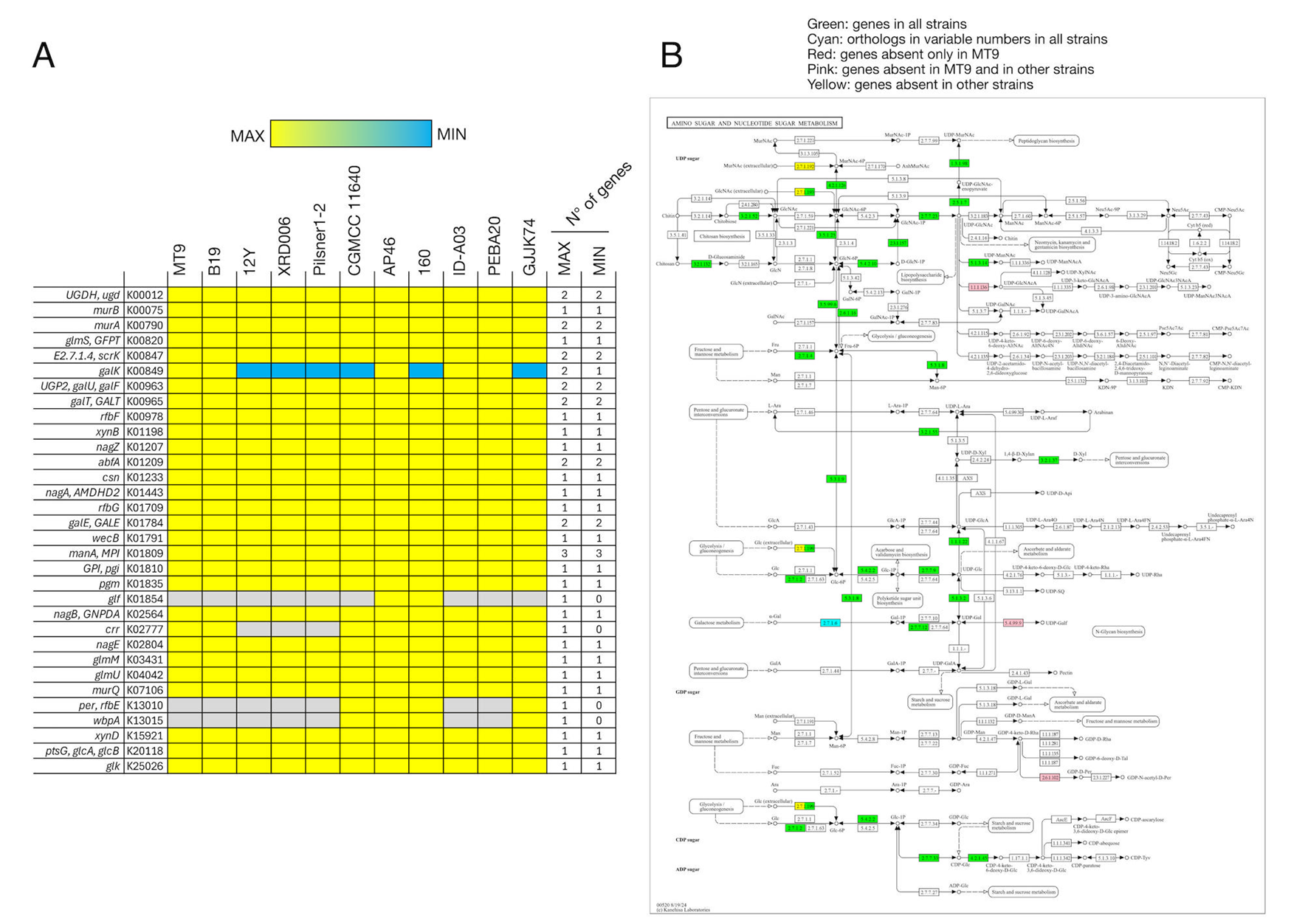

Supplement: Supplementary file 13 — (PNG 741 KB) [file 248_2025_2531_Fig17_ESM.png]

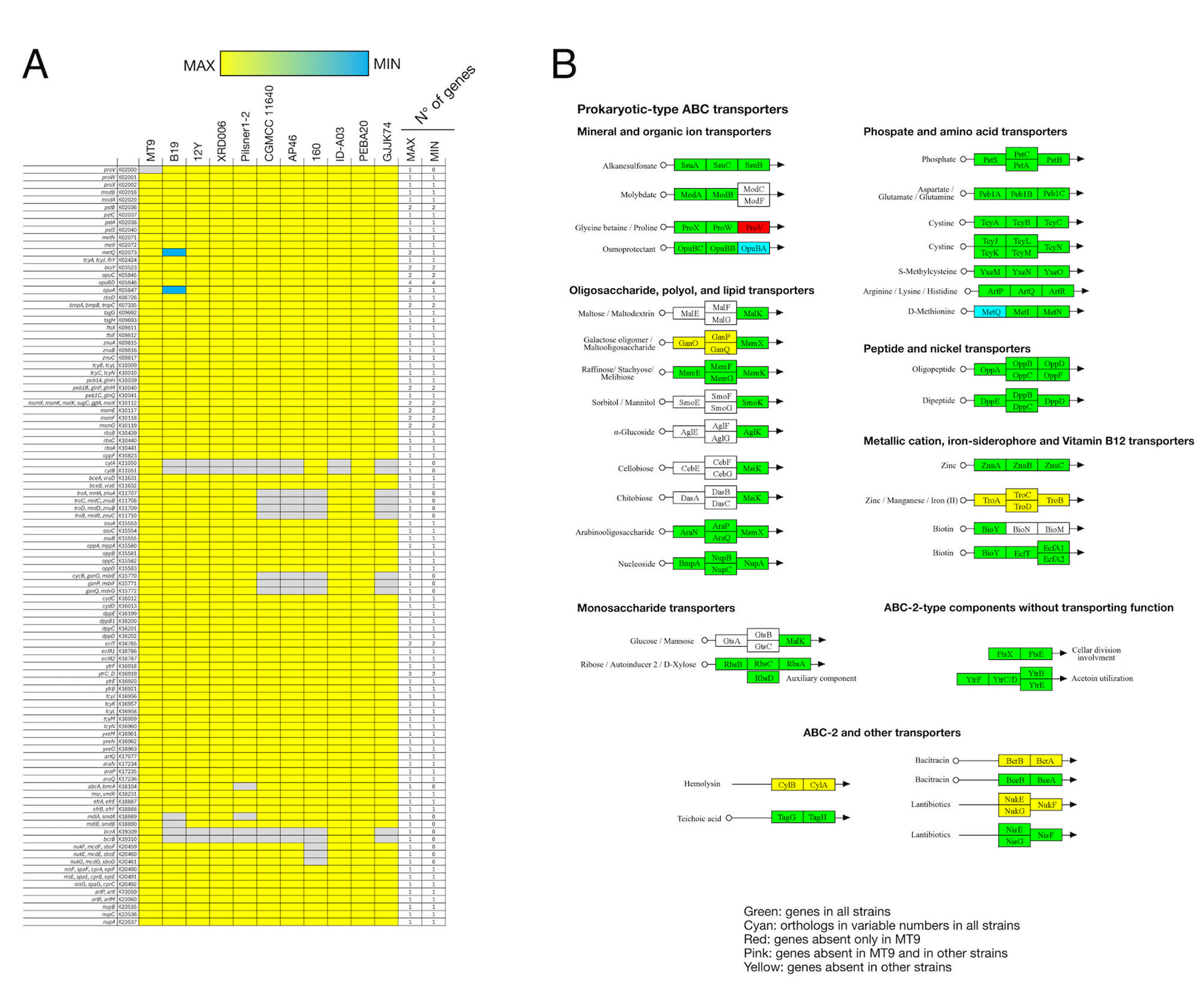

Supplement: Supplementary file 15 — (PNG 900 KB) [file 248_2025_2531_Fig18_ESM.png]

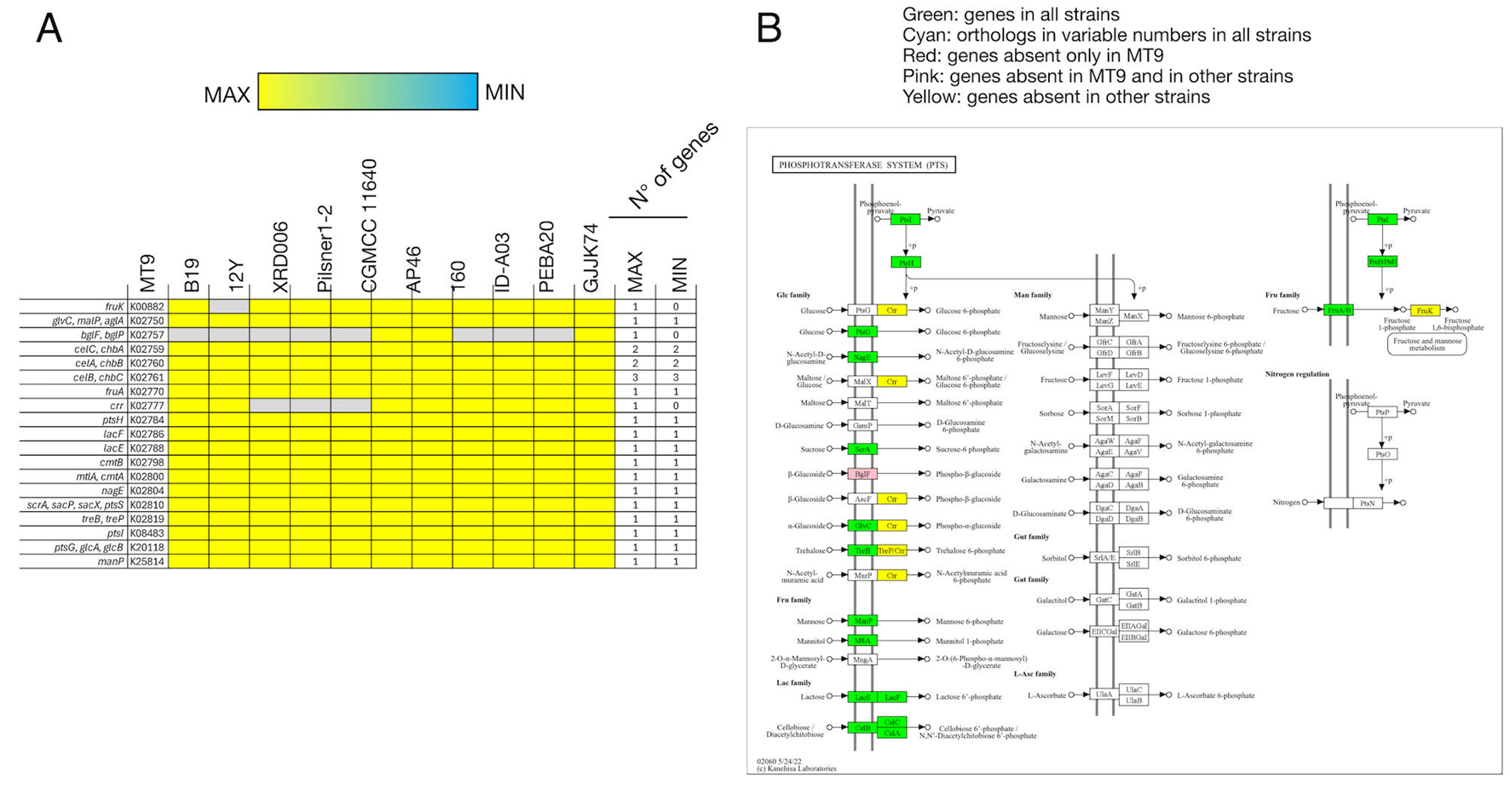

Supplement: Supplementary file 17 — (PNG 487 KB) [file 248_2025_2531_Fig19_ESM.png]

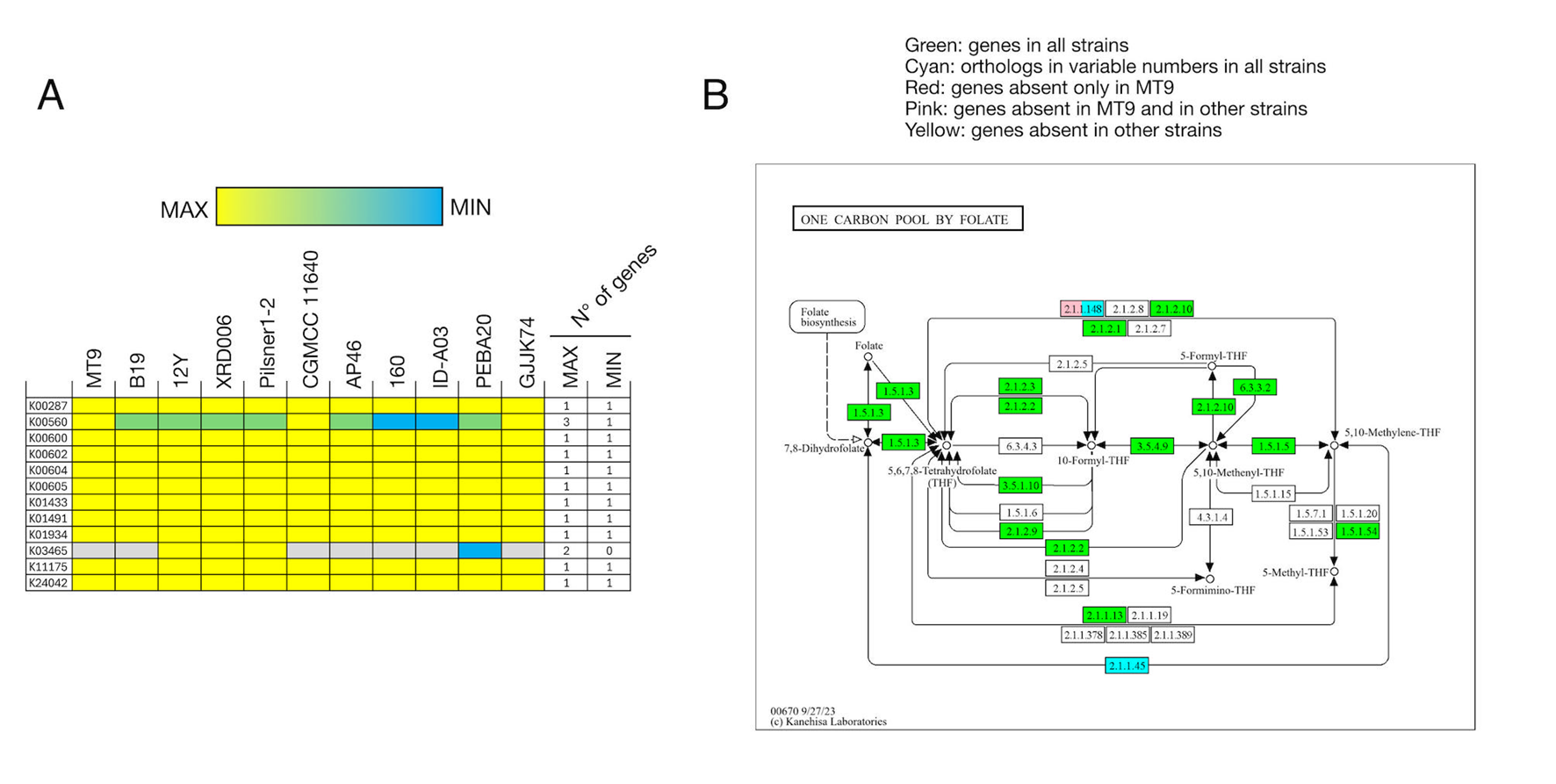

Supplement: Supplementary file 19 — (PNG 328 KB) [file 248_2025_2531_Fig20_ESM.png]

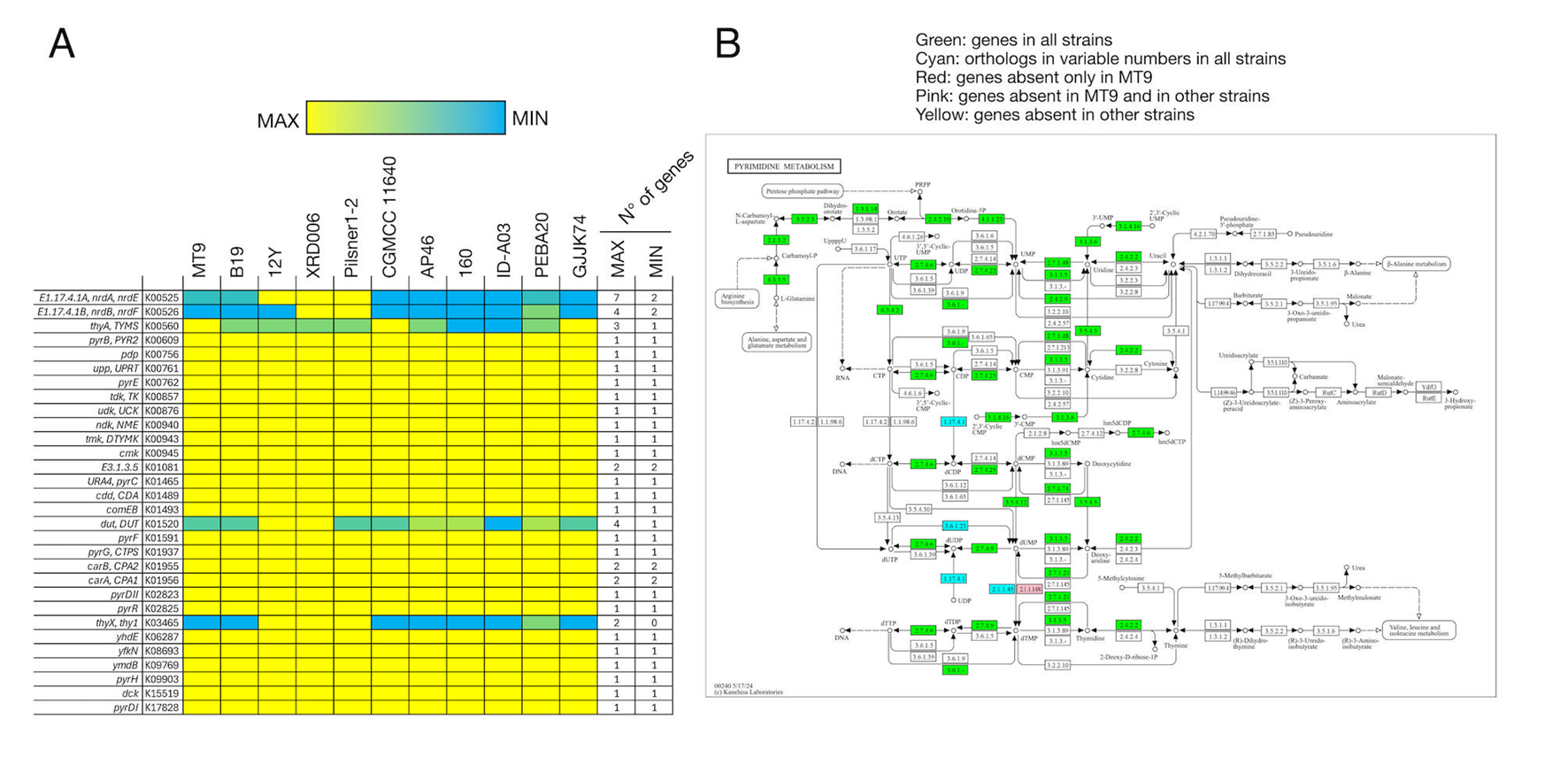

Supplement: Supplementary file 21 — (PNG 523 KB) [file 248_2025_2531_Fig21_ESM.png]

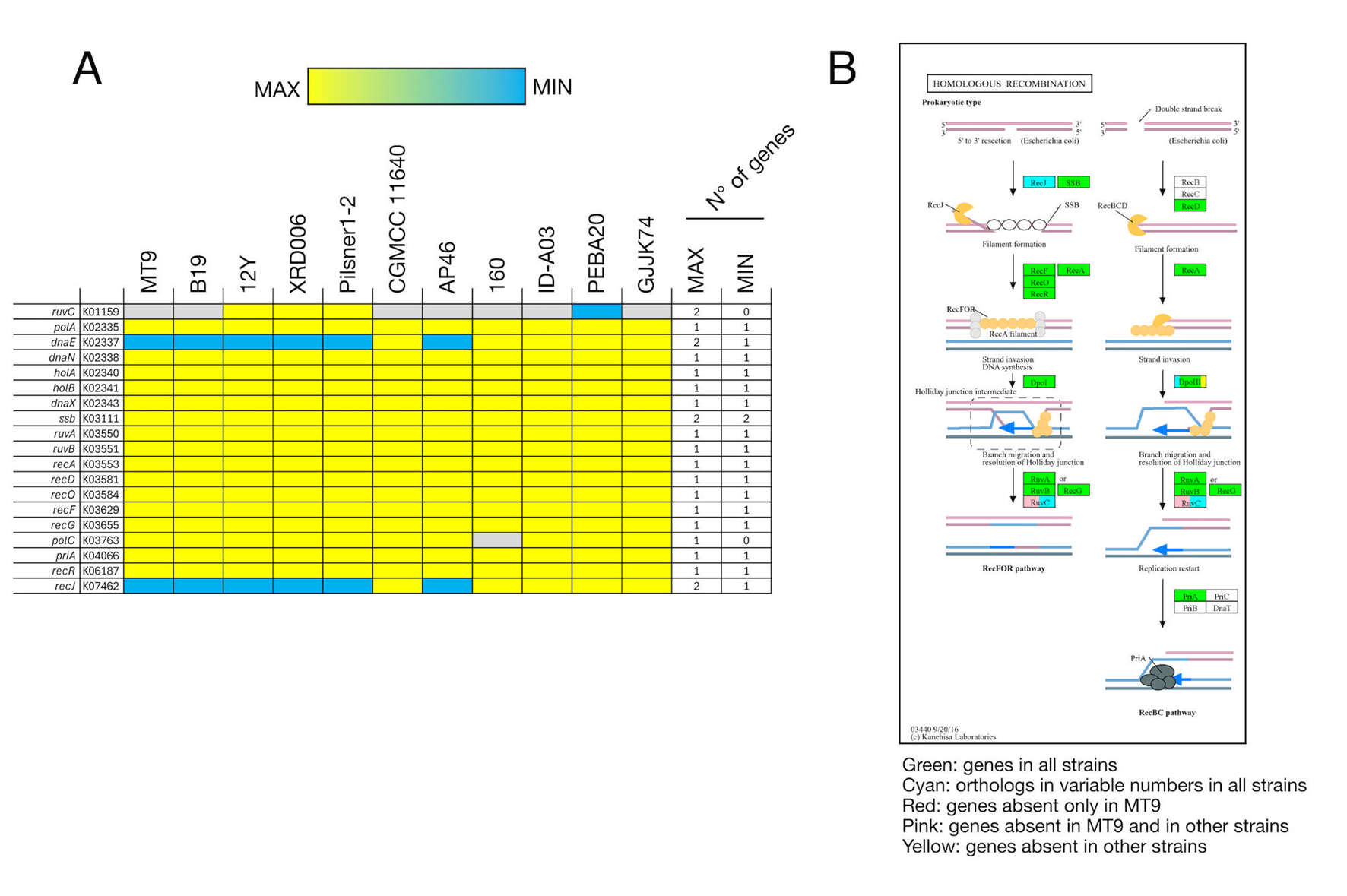

Supplement: Supplementary file 23 — (PNG 463 KB) [file 248_2025_2531_Fig22_ESM.png]

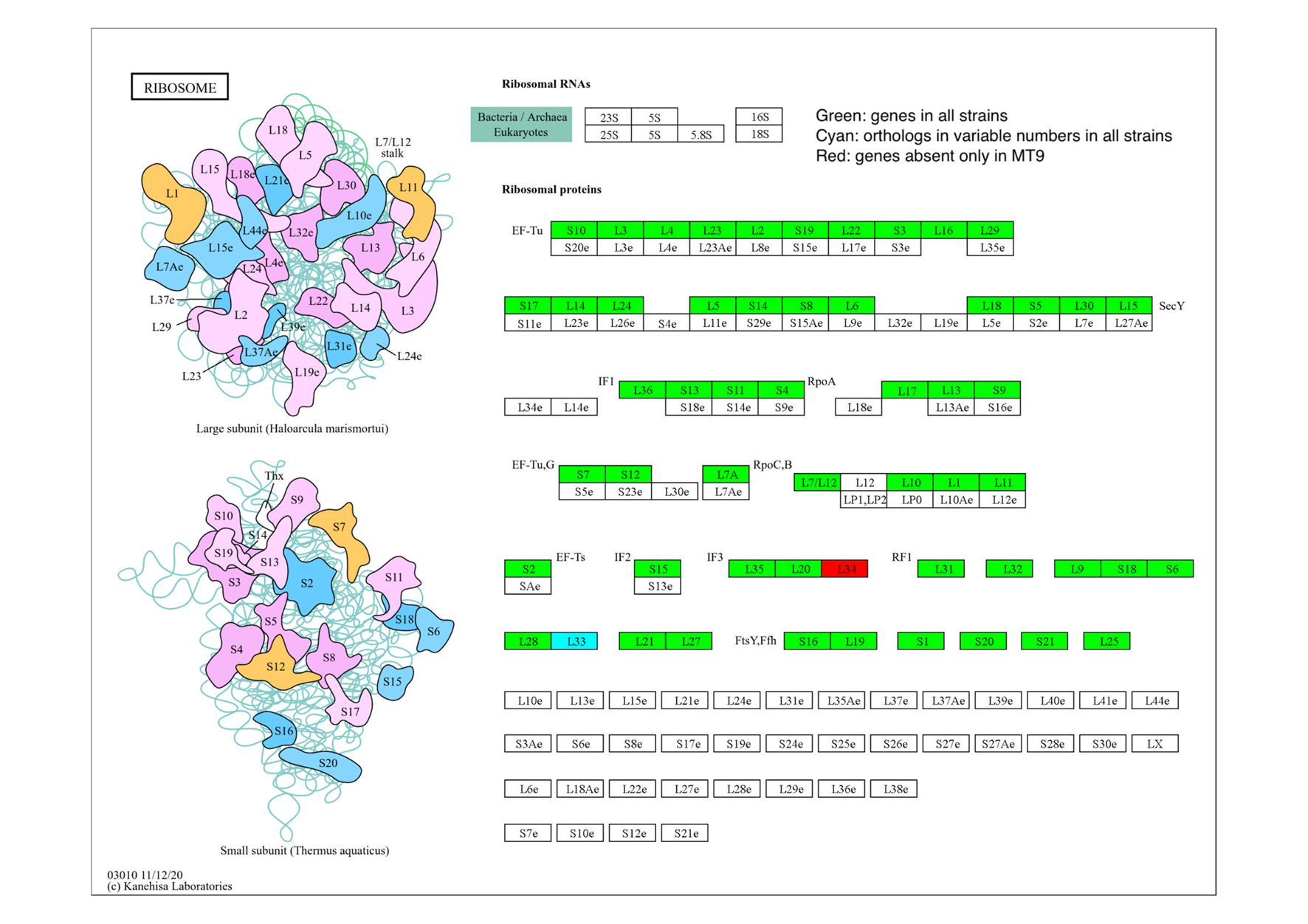

Supplement: Supplementary file 25 — (PNG 755 KB) [file 248_2025_2531_Fig23_ESM.png]

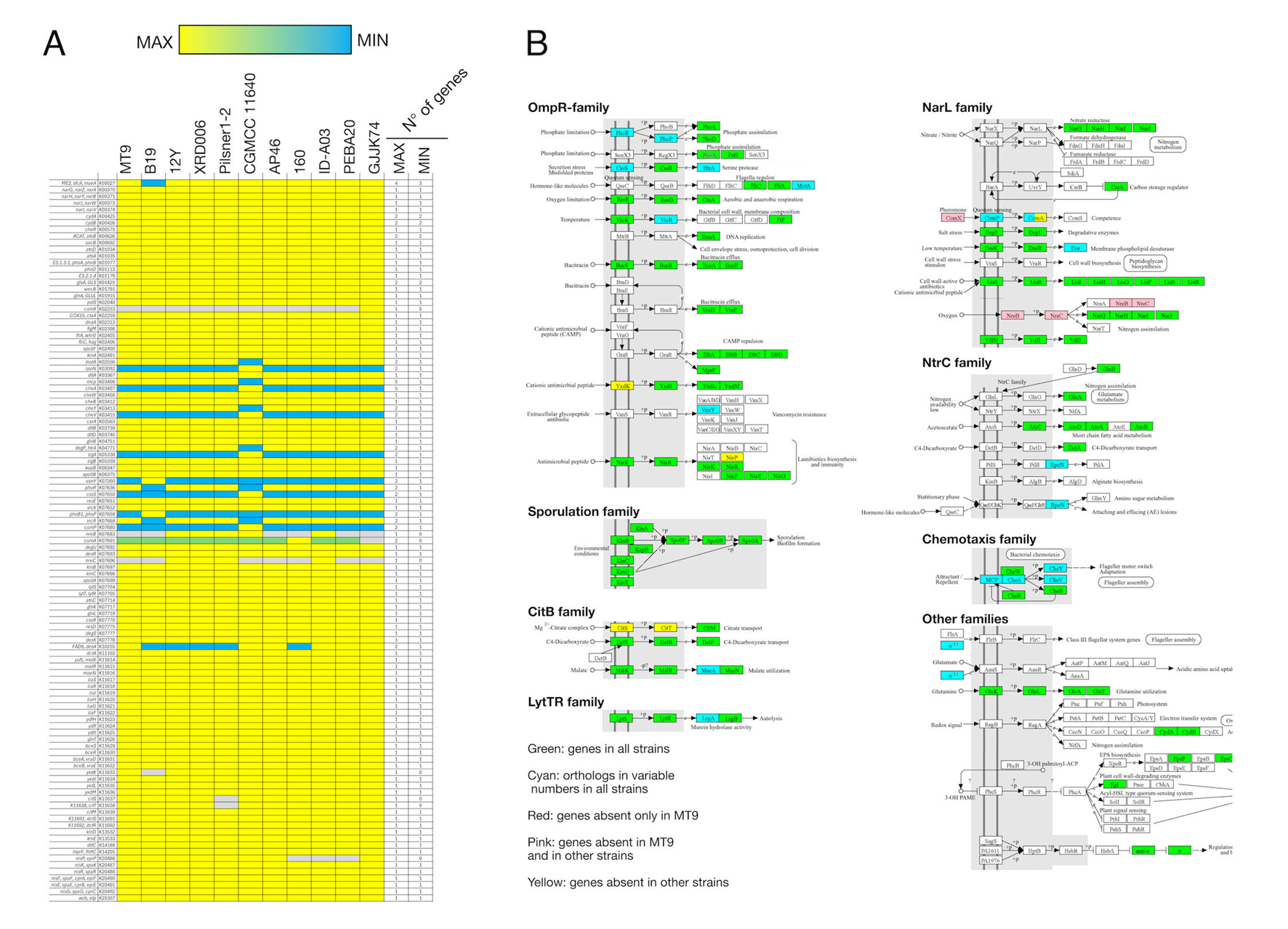

Supplement: Supplementary file 27 — (PNG 879 KB) [file 248_2025_2531_Fig24_ESM.png]

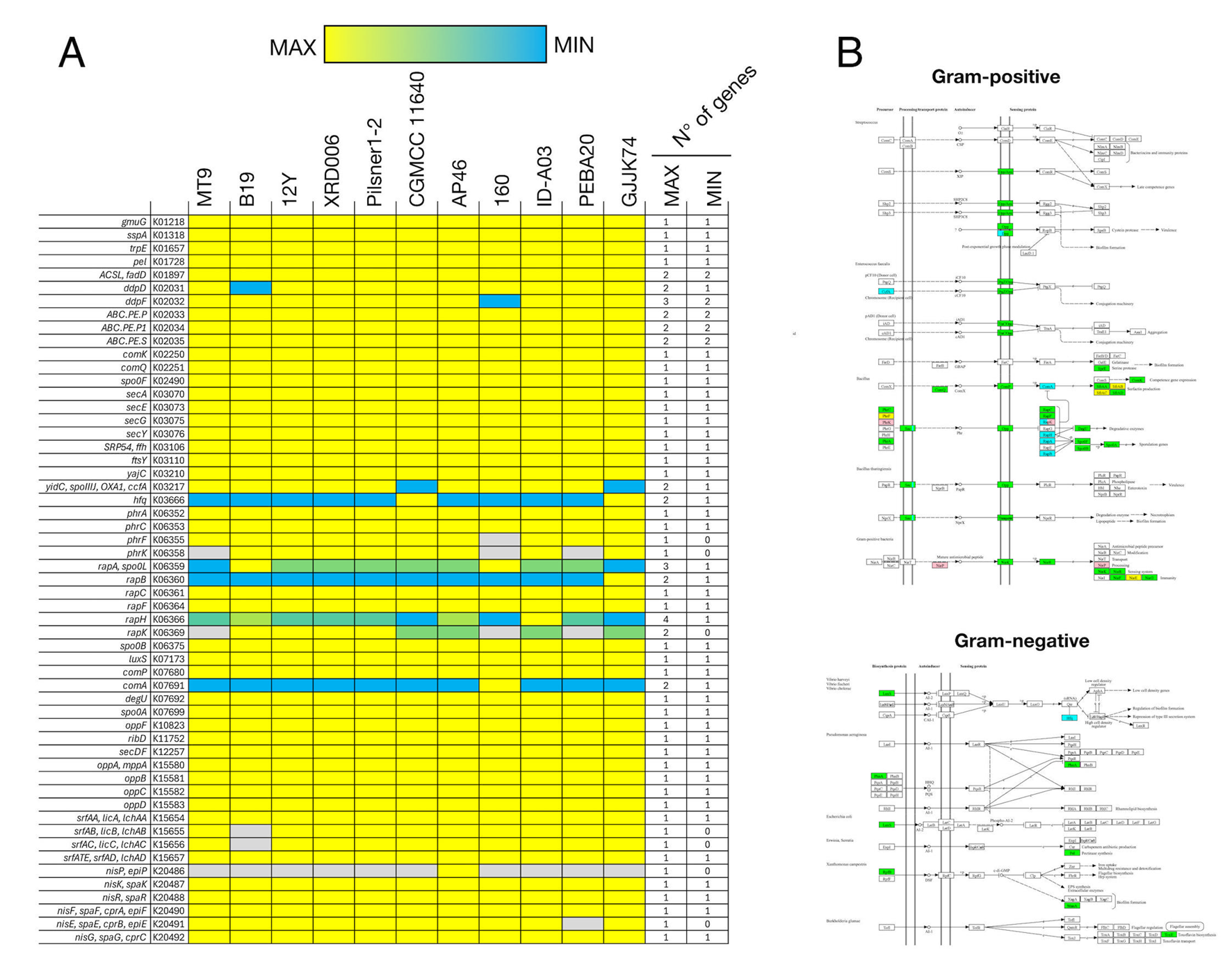

Supplement: Supplementary file 29 — (PNG 747 KB) [file 248_2025_2531_Fig25_ESM.png]
